# Scientists without borders: lessons from Ukraine

**DOI:** 10.1093/gigascience/giad045

**Published:** 2023-07-27

**Authors:** Walter Wolfsberger, Karishma Chhugani, Khrystyna Shchubelka, Alina Frolova, Yuriy Salyha, Oksana Zlenko, Mykhailo Arych, Dmytro Dziuba, Andrii Parkhomenko, Volodymyr Smolanka, Zeynep H Gümüş, Efe Sezgin, Alondra Diaz-Lameiro, Viktor R Toth, Megi Maci, Eric Bortz, Fyodor Kondrashov, Patricia M Morton, Paweł P Łabaj, Veronika Romero, Jakub Hlávka, Serghei Mangul, Taras K Oleksyk

**Affiliations:** Department of Biological Sciences, Oakland University, Rochester, MI 48309-4479, USA; Department of Clinical Pharmacy, USC Alfred E. Mann School of Pharmacy and Pharmaceutical Sciences, University of Southern California, Los Angeles, CA 90033, USA; Department of Biological Sciences, Oakland University, Rochester, MI 48309-4479, USA; Institute of Molecular Biology and Genetics of National Academy of Sciences of Ukraine, Kyiv Academic University, Kyiv 03143, Ukraine; Institute of Animal Biology, National Academy of Agrarian Sciences (NAAS) of Ukraine, Lviv 79034, Ukraine; National Scientific Center “Institute of Experimental and Clinical Veterinary Medicine,” Kharkiv 61023, Ukraine; Institute of Economics and Management, National University of Food Technologies (NUFT) of Ukraine, Kyiv 01601, Ukraine; Department of Anesthesiology and Intensive Care, P.L. Shpyk NUHC Ukraine, Kyiv 04112, Ukraine; Department of Finance and Business Economics, Marshall School of Business, University of Southern California, Los Angeles, CA 90089, USA; Department of Medicine, Uzhhorod National University, Uzhhorod 88000, Ukraine; Department of Genetics and Genomic Sciences, Icahn School of Medicine at Mount Sinai, New York, NY 10029, USA; Department of Food Engineering, Izmir Institute of Technology, Urla, Izmir 35430, Turkey; Department of Biology, University of Puerto Rico at Mayagüez, Mayagüez 00681, Puerto Rico; Aquatic Botany and Microbial Ecology Research Group, Balaton Limnological Research Institute, Tihany 8237, Hungary; Stritch School of Medicine, Loyola University Chicago, Maywood, IL 60153, USA; Department of Biological Sciences, University of Alaska, Anchorage, AK 99508, USA; Institute of Science and Technology Austria, Klosterneuburg 3400, Austria; Department of Sociology, Department of Public Health, Wayne State University, Detroit, MI 48202, USA; Małopolska Centre of Biotechnology, Jagiellonian University, Kraków 30-348, Poland; Department of Neurobiology, University of Utah, Salt Lake City, UT 84112, USA; Price School of Public Policy, University of Southern California, Los Angeles, CA 90089-3333, USA; Masaryk University, Brno 6017, Czech Republic; Department of Clinical Pharmacy, USC Alfred E. Mann School of Pharmacy and Pharmaceutical Sciences, University of Southern California, Los Angeles, CA 90033, USA; Department of Computational Biology, University of Southern California, Los Angeles, CA 90033, USA; Department of Biological Sciences, Oakland University, Rochester, MI 48309-4479, USA; Department of Biology, Uzhhorod National University, Uzhhorod 88000, Ukraine

**Keywords:** conflicts, scholars, Europe, Ukraine, Russia, science, funding, remote learning, scholarship opportunities, bioinformatics

## Abstract

Conflicts and natural disasters affect entire populations of the countries involved and, in addition to the thousands of lives destroyed, have a substantial negative impact on the scientific advances these countries provide. The unprovoked invasion of Ukraine by Russia, the devastating earthquake in Turkey and Syria, and the ongoing conflicts in the Middle East are just a few examples. Millions of people have been killed or displaced, their futures uncertain. These events have resulted in extensive infrastructure collapse, with loss of electricity, transportation, and access to services. Schools, universities, and research centers have been destroyed along with decades’ worth of data, samples, and findings. Scholars in disaster areas face short- and long-term problems in terms of what they can accomplish now for obtaining grants and for employment in the long run. In our interconnected world, conflicts and disasters are no longer a local problem but have wide-ranging impacts on the entire world, both now and in the future. Here, we focus on the current and ongoing impact of war on the scientific community within Ukraine and from this draw lessons that can be applied to all affected countries where scientists at risk are facing hardship. We present and classify examples of effective and feasible mechanisms used to support researchers in countries facing hardship and discuss how these can be implemented with help from the international scientific community and what more is desperately needed. Reaching out, providing accessible training opportunities, and developing collaborations should increase inclusion and connectivity, support scientific advancements within affected communities, and expedite postwar and disaster recovery.

## Background

Countries all over the world face a variety of hardships, from political upheaval, wars, economic crises, and natural disasters, where [1] millions of people's lives are lost or destroyed and require a huge amount of planning, aid, and resources to rebuild. For this review, we want to focus on how to aid scholars and students who have become displaced, disconnected from their professional communities, and face an unknown future. In the face of disaster, established researchers lose funding, workplaces, laboratories—including samples and data that may never be recovered, lines of communication, and collaboration. Students, undergraduate as well as graduate, have their careers interrupted, and entire generations of talented youth become disengaged, disconnect from educational opportunities, and drop out of their research careers. Groups facing such crises can turn for help to their colleagues in the international community, and with every success and failure of these efforts, we can learn how to face the next global challenge.

There is value in diverse scientific communities, and if we strive to have inclusion, equity, and diverse perspective in the scientific community, joining hands with scientists in crises is the first important step. Placing resources, time, and effort into our struggling colleagues follows a great scientific tradition of providing opportunities to capable minds, even in tough situations. Einstein, Tesla, and Curie, all of whom escaped war and/or persecution, are evidence that humanity can benefit in great ways from international scientific collaborations. With the scientific world becoming increasingly interconnected, the capability of the international community to aid researchers with hardships is potentially much easier than would have been possible before investment and engagement in worldwide projects. Collaborative cooperation, such as that seen for the Global Alliance for Genomics and Health [2], the International Brain Initiative [3], the Human Genome Project [4], and the Large Hadron Collider [5], emerges every day to address global scientific challenges. Landmark projects like these have been hugely successful because they allowed the exchange of resources, expertise, and data on a global scale. The combined knowledge of collaborative groups is widely shared, discussed, and disseminated through scientific conferences (in-person, hybrid, and remote) and open publishing. Online preprints, journals, and open-access publishing have revolutionized the distribution of knowledge by allowing easier information access. Global research networks have emerged across many fields, and initiatives like the open access and open data movements [6] have further promoted knowledge sharing. Finally, UNESCO's Recommendation on Open Science [7] is a valuable tool for supporting scientists in hardship areas that can be implemented by providing funding for open science projects, training and supporting scientists on open science tools and practices, and developing open science infrastructure to ensure they have the resources and support needed to carry out their work and contribute to scientific progress.

Global research networks are growing rapidly to help scientists connect and collaborate, and technology is playing a key role in this process. With the increasing availability of high-speed internet and digital tools, scientists from around the world are now able to connect and collaborate in real time, regardless of their location. Web-based technologies behind collaborative scientific projects require less in-person engagement than a decade ago. This is especially true in the emerging omics sciences (i.e., genomics, transcriptomics, proteomics, and bioinformatics) [8]. Early on, the biggest bottlenecks in the Human Genome Project and 1,000 Genomes Project were in data acquisition [9], where international collaborations required specific laboratory skills and on-site resources for sample collection and sequencing. Today, medical and other phenotypic data can be easily accumulated and shared between researchers and their institutions digitally, and loads of low-cost DNA, RNA, and protein sequencing data from research and high-throughput screening are becoming available and can be jointly analyzed by international groups engaged in genomics and bioinformatics research [10–13]. The major bottleneck facing these groups in the decades after the publication has been the lack of access to powerful hardware and software to analyze torrents of incoming data. Now, this issue can be resolved with advanced clusters, increased storage, and cloud computing. However, a collaboration that relies on building large international networks can only grow by maximizing global human engagement [14].

Online platforms and social media help create and connect networks that transcend international borders, where researchers can share ideas, collaborate on projects, and engage in discussions with colleagues from different countries and backgrounds, while the emerging cloud computing, data visualization, and artificial intelligence tools lead to more efficient research coordination and faster discoveries. These technologies are breaking down barriers and creating opportunities for scientists to connect in ways that were not possible just a few years ago and are helping to accelerate scientific progress around the world. We believe that providing opportunities to groups of scientists temporarily disconnected as a result of war or isolated as a result of political, economic, or ecological crisis is critical to global science. In this review, we tried to identify the most effective mechanisms to support researchers in countries facing hardship using a case study based on the activities carried out to help researchers during the year of conflict in Ukraine [1].

## The Global Response of the Scientific Community to the War in Ukraine

More than a year of war in Ukraine is only one example of the scientific community responding to a major crisis. Immediately after the start of the invasion on 24 February 2022, several online resources were created by volunteers who were concerned with the negative impact on the work of their colleagues. These were mostly open spreadsheets listing short- and longer-term positions, internships, and other types of support that were communicated [15] (Table 1). Some of these resources were spontaneous, originating from concerned individuals and groups. In one example, an ad hoc group from the University of Oregon posted a list of laboratories willing to support displaced Ukrainian scientists that quickly gained in popularity and, 40 days after its inception, featured more than 2,000 listings for positions in Europe and the United States (Labs Supporting Ukrainian Scientists, Table 1). A similar list was also created at Sam Houston University (Ukrainian Scholar Placement Database, Table 1). Following these, a larger collaborative initiative, #ScienceForUkraine [16], emerged just 2 days after the war began as an online collaboration of hundreds of international scholars volunteering to build a central database of international opportunities available to Ukrainian researchers. As the war progressed, several other initiatives emerged intending to support Ukrainian scholars, including the Ukrainian Scholar Placement Database developed by Sam Houston University (Table 1), which compiled a list of funding programs available to researchers in Ukraine. The activity of the emerging groups was not limited to research opportunities. Simultaneously, other communities such as Saving Ukrainian Cultural Heritage Online (SUCHO, Table 1) engaged in web-archiving, digitizing, and preserving heritage from Ukrainian cultural institutions (see the full list in Table 1). Appeals were made to support those who were already studying abroad but could no longer continue their programs [17].

**Table 1:** Links to resources for Ukrainian scientists that have been mentioned in this article. This list is not exhaustive as new opportunities are published every day. For a more complete representation, please search databases such as *#ScienceForUkraine* and others mentioned below.

Resource	Description	Location	Link
#ScienceForUkraine	A community group collecting and disseminating information about support opportunities.	International	https://scienceforukraine.eu
Council for At-Risk Academics (CARA)	A nongovernmental organization collaboration of UK universities to provide relief of suffering and the defense of learning and science	UK	https://www.cara.ngo
ERA4Ukraine	An EU initiative that supports researchers of Ukraine by providing them with an overview of all existing actions at European and national levels	EU	https://euraxess.ec.europa.eu/ukraine
European Federation of Academies of Sciences and Humanities (ALLEA)	European fund for displaced scientists	EU	https://allea.org/european-fund-for-displaced-scientists
ERC4Ukraine	European Research Council (ERC) initiative that provides temporary employment to refugee researchers and support staff	EU	https://erc.europa.eu/apply-grant/erc-ukraine
The Guild	The Guild of European Research-Intensive Universities Association support initiative	EU	https://www.the-guild.eu/resources/the-guild-s-universities-supporting-researchers-ac.html
IIE Scholar Rescue Fund	A global program that arranges funds and supports fellowships for threatened and displaced scholars	EU	https://www.scholarrescuefund.org
Labs Supporting Ukrainian Scientists	An online database to help find positions for displaced scholars from Ukraine at the University of Oregon	USA	https://tinyurl.com/yanb37ck
LeCollègede France	A fund featuring state-sponsored opportunities for displaced scientists	France	https://www.college-de-france.fr/
OEG Connect	An open education resource to connect, share, and work together to make learning accessible	International	https://connect.oeglobal.org/
Philipp Schwartz Initiative of the Humboldt Foundation	A program to help researchers subject to significant personal threat in their country to continue their work at German universities	Germany	https://www.humboldt-foundation.de
MSCA4Ukraine	A subsidiary of the EU's Marie Skłodowska-Curie Actions, to provide fellowship support to researchers from Ukraine	EU	https://sareurope.eu/msca4ukraine/
Saving Ukrainian Cultural Heritage Online (SUCHO)	A group of volunteers to web-archive digitize and preserve heritage from Ukrainian cultural institutions (no research support)	International	https://www.sucho.org
Scholars at Risk (SAR)	International network to protect threatened scholars and promote academic freedom around the world. Cooperating with several national science foundations in Europe	International	https://www.scholarsatrisk.org
Scientists and Engineers in Exile or Displaced (PAS/NAS SEED) program	A collaboration of national academies to award grants	USA and Poland	https://www.nationalacademies.org/our-work/scientists-and-engineers-in-exile-or-displaced-seed-program
Shevchenko Emergency Fund	A fund to support scholars by the Shevchenko Scientific Society, the largest and oldest Ukrainian scientific organization outside of Ukraine	USA	https://shevchenko.org
Swiss National Science Foundation	A state-sponsored organization that has opportunities for displaced scientists	Switzerland	https://www.snf.ch/en
The Association for Slavic, East European & Eurasian Studies	Resource database to help scholars from Ukraine	USA	https://www.aseees.org/resources/help-displaced-scholars-ukraine
The UK-Ukraine Twinning Initiative	An institution-to-institution collaboration model to lay the foundations of strong partnerships between the UK and Ukrainian universities supported by grants from the UK Research and Innovation (UKRI) funding agency.	UK and Ukraine	https://www.twinningukraine.com/
Ukrainian Global University Initiative	A consortium of leading Ukrainian educational institutions and organizations to support Ukrainian student scholars with the opportunities for quality education and research	Ukraine	https://uglobal.university
Ukrainian Scholar Placement Database	An online database to help find positions for displaced scholars from Ukraine at Sam Houston State University	USA	https://tinyurl.com/2f8csafa

Existing cooperation networks that were in place before the war were used to rapidly launch or amplify the support measures. A group of initiatives was rising from existing prewar structures, notably national societies and academies. For example, the Office of Policy and Global Affairs, a division of the US National Academies, previously engaged in science diplomacy among different nations, in collaboration with the Polish Academy of Sciences, quickly created the Scientists and Engineers in Exile or Displaced initiative to support several hundreds of Ukrainian scholars with short-term stipends for studies outside of Ukraine (The Polish Academy of Sciences and the U.S. National Academy of Sciences (PAS-NAS) SEED initiative, Table   1). The publicly funded Shevchenko Scientific Society (the largest and the oldest Ukrainian scientific organization outside of Ukraine founded in 1873) started to create opportunities for the displaced scientists supported by its US- and Canada-based members. Most of the national academies in the EU block issued supportive statements to Ukraine, and within weeks, the European Federation of Academies of Sciences and Humanities (Table 1), using a donation from the Breakthrough Prize Foundation, started hosting scholars displaced by the war. After millions of refugees crossed the EU border and settled in the various European countries, more opportunities were created by national organizations such as the Swiss National Science Foundation, the Polish Academy of Sciences, and Le Collège de France. The European Commission opened the European Research Area portal for Ukraine, and Horizon Europe, with its complementary Euratom Research and Training Programme, offered free participation to support the Ukrainian scientific community. The MSCA4Ukraine consortium, funded under the EU's Marie Skłodowska-Curie Actions, provides fellowship support for hundreds of displaced researchers from Ukraine.

However, national academies were only 1 source of support. More active were global research networks and discipline-specific international associations. International organizations, such as Scholars at Risk (Table 1), formed as far back as 1999 to protect threatened scholars and promote academic freedom around the world, and others (IIE Scholar Rescue Fund, Philipp Schwartz Initiative of the Humboldt Foundation in Germany, Council for At-Risk Academics in the United Kingdom, and others; Table 1), backed by governments, philanthropists, and large corporate sponsors, rapidly focused their efforts and started creating opportunities for the displaced Ukrainians as well. The UK-based twinning initiative, an institution-to-institution collaboration model, incorporated the UK-Ukraine Research and Innovation twinning grants from UKRI's Research England to enable twins to further their research and innovation collaborations. In a relatively short time, individual universities, research institutes, and other academic organizations from all across the globe started offering scholarships, paid positions, and other types of support on their own. Volunteer initiatives and the unwavering support received from an array of universities, academic institutions, scientific societies, publishers, and funding agencies worldwide were met with great gratitude from the Ukrainian scientific community [18]. This unprecedented widespread support will be crucial on the path to recover and mitigate the consequences of the war for academic and scientific communities and their institutions in the country. The networks established early at the start of the war laid a foundation for the further integration of Ukrainian scientists into the global community and will become grounds for the future success of more formal organizational initiatives involving Ukraine that are being developed at this time (i.e., Ukrainian Global University Initiative, Horizon Europe, etc.).

Despite these opportunities, so far, only a fraction of scholars could physically relocate abroad and take advantage of the opportunities offered to them by the global response; the rest remained in Ukraine. This is a complex issue. According to the rules of martial law in Ukraine, males between the ages of 18 and 60 cannot leave the country. Many academics joined the Territorial Defense or were drafted into the Ukrainian Armed Forces [19]. Some did not want to leave their families and homes in Ukraine for a short research opportunity abroad, or they needed to take care of their children and the elderly who could not come along. Even among those who could leave, many were discouraged by the bureaucratic process of obtaining long-term visas.

Those staying in Ukraine found themselves in the most unstable and vulnerable situation. Many universities, especially those in the east of the country, are either closed or relocated to safer areas. The budgets of the state-run universities were cut to save funds for the war effort. While the base salaries for teaching are usually maintained, these were traditionally already very low and commonly supplemented by income from funded research and overtime work, which are now dropped [20]. The national research programs have been plagued by the issues associated with the war, and the funding has mostly been cut off or frozen. Despite the dire situation, many researchers in Ukraine continued to maintain their academic and research activities.

The Ukrainian crisis has several distinct challenges, and it invoked a complex response with many underlying factors, including historical, cultural, economic, and political factors. The initial reaction of the scientific community was to provide opportunities for refugees fleeing the country. Some of the recent examples resulted in many scholars seeking refuge. Under the current political regime, about 2,000 highly skilled Venezuelan scientists responsible for the publication of a third of all scientific papers from Venezuela and representing 15% of the Venezuelan research community have left their academic posts and fled abroad [21]. The recent crisis associated with the withdrawal of US troops from Afghanistan forced many researchers to flee, and those who remained lost funding and faced a threat of persecution [22]. The Syrian civil war has been ongoing since 2011 and has caused significant humanitarian, economic, and political challenges, producing science refugees: academics and scientists who required help outside their country [23]. Initially, it seemed that the war in Ukraine would produce a similar result, and the research community immediately began preparing for the influx of researchers who needed a place to live and work outside of their country.

## Analysis of the Opportunities for Ukrainian Scientists Listed in the #ScienceForUkraine Database

To understand what types of opportunities have been offered to Ukrainian scientists during the first year of the war, we surveyed and classified the entries posted on the #ScienceForUkraine website 1 year after the start of the invasion (February 2022–February 2023). The goal of this initiative was to help provide support for the Ukrainian academic community and also streamline any setbacks for the Ukrainian scientific community [16]. While this database may be incomplete, and some entries have been deleted (personal communication), it is still the largest publicly available database that can be surveyed for the types of opportunities that have been offered.

**Figure 1:**
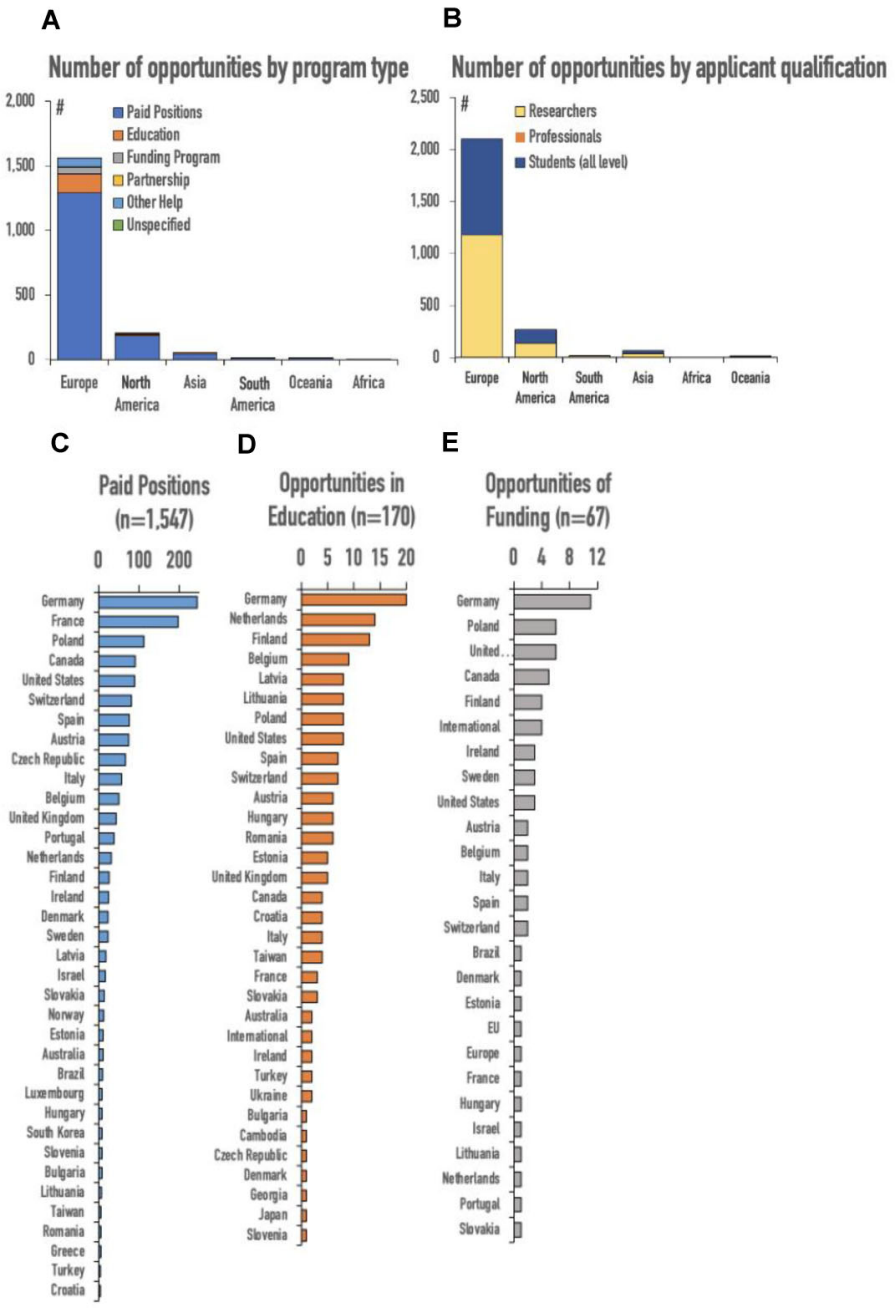
Locations of opportunities for Ukrainian scientists in the #ScienceForUkraine database in 1 year since the start of the conflict (February 2022–February 2023). (A) Number of opportunities by program type. The majority of the 1,871 listed opportunities can be classified as paid positions. These include research and postdoctoral fellowships, as well as some researcher jobs. Most of these are in Europe (1,293), with North America as a distant second (181). Educational opportunities and funding programs were distributed in similar proportions. (B) Number of opportunities by applicant qualification. Opportunities were distributed roughly equally between researchers and students of every level, with very few indicated as “professional.” (C–E) Distribution of different types of opportunities (listed in A) by country. The total number of opportunities per category is given in parentheses. Germany had the most opportunities in all categories.

A review of the #ScienceForUkraine database shows a clear trend toward in-person and a lack of remote opportunities (Table 2, Fig. 1). #ScienceForUkraine volunteers tried to achieve this by raising awareness among research communities across the world about the situation, posting about new opportunities for students and scholars to receive opportunities across the world, and working with various European funding organizations to help support the academic community. We used the default classification of this database into “paid positions,” “educational,” “funding programs,” and “partnerships” (Fig. 1A). Most of the 1,871 listed opportunities listed were “paid positions.” Opportunities labeled as “educational” and “funding programs” were distributed in similar proportions (Table 2, Fig. 1A).

**Table 2:** Locations of opportunities for Ukrainian scientists in the #ScienceForUkraine database

Type	All Available	Europe	North America	Asia	South America	Oceania	Africa
Paid positions	1,456 (83%)	1,293	181	44	11	12	2
Education programs	170 (9%)	145	12	8	0	2	0
Funding programs	67 (4%)	53	8	1	1	0	0
Partnerships	6 (<1%)	1	2	0	1	1	0
Other help	77 (4%)	68	5	0	0	0	0
Unspecified	5 (<5%)	3	0	1	1	0	0
Total		1,563	208	54	14	15	2

Overall, all classes of opportunities were distributed roughly equally between “researchers” and “students” of every level, with very few indicated as “professional” (Fig. 1B). It is difficult to classify these into distinct subcategories, but generally, they included research and postdoctoral fellowships, as well as some researcher jobs. Unsurprisingly, most of them were located in Europe (*n* = 1,293), with North America (the United States and Canada) as a distant second (*n* = 181). Germany had the most paid positions, followed by France, Poland, Canada, and the United States (Fig. 1C). Germany also had the most opportunities in education as well as funding programs (Fig. 1D, E).

Generally, since most of these initiatives were trying to deal with the refugee crisis at hand, their success relied largely on the readiness of the Ukrainian scientists to move abroad. The review of the #ScienceForUkraine database demonstrates that most entries require travel and shows the relative scarcity of remote opportunities for scholars who cannot leave the country during the time of war: only 52 positions (out of 1,871 available) specifically mentioned the word “remote,” and most of them suggest a possibility of relocation. Despite the vital importance of initiatives offering temporary or permanent positions, they target opportunities for only a fraction of the scientists who left or were able to leave the country. As a result, very often the existing opportunities may go unfulfilled, which would frustrate those who offer them, as well as those who could not use them.

## Effective and Feasible Mechanisms to Support Scientists and Students from Ukraine

We hope to use the ongoing struggle in Ukraine to initiate the conversation about effective and feasible mechanisms for the support of scholars and students at risk. There is a need for discussion on the feasibility and efficacy of various approaches to short-term and long-term strategies that help scholars during the active phase of conflicts and postwar reconstruction periods. Specifically, we want to bring attention of the international community to creating scalable mechanisms that can be offered to several thousands of scientists who have left their respective countries versus those who are remaining in the country. It would only make sense to complement the opportunities that require travel with the ones that can be more suitable for the scientists staying in Ukraine: offering a wider range of remote positions, engaging the scientific community in the global community, and offering effective training to address any existing knowledge gaps [1]. This combined strategy would help Ukrainian science to endure difficult times, secure their development, and help further integrate them into the international scientific community. This will also set an example for other scientific communities at risk. We use Ukraine as an example from our collaborations, but the recommendations herein can be used to support research initiatives of scientists in other countries.

The effective remote opportunities described here could be sorted into two groups (Table 2). In the first group, there are efforts that are directed toward and imply establishing contact with a specific scientist or a group of scientists in Ukraine (Table 3, A–D). In the second group, there are venues to help Ukrainian researchers indirectly, without knowing or contacting specific researchers or scientific groups (Table 3, E–H).

**Table 3:** Mechanisms to support scientists and students from countries experiencing hardship. Group 1 (A–D) includes efforts that are directed toward and imply establishing contact with a specific scientist or a group of scientists in Ukraine. In group 2 (E–H), there are venues to help Ukrainian researchers indirectly, without knowing or contacting specific researchers or scientific groups..

	#	Mechanism	Description
Group 1	A	Establish direct remote research collaborations	A scientist or researcher can collaborate remotely with Ukrainian scientists directly on common research projects.
	B	Donate funds and provide access to resources	Funds can be donated directly to research institutions to support their work.
	C	Share research opportunities	Inform colleagues about opportunities that could be missed because of the limited access or language barriers.
	D	Participate in online events inside the country	Take part in webinars, online conferences, and workshops put together by local organizers.
Group 2	E	Promote publications in the scientific journals	Encourage journals to support researchers by waving publication fees, provide translations and editorial services, and include versions of published papers in the authors’ native languages.
	F	Promote participation in international conferences	Encourage organizers of scientific conferences to provide free or discounted access to registration and memberships.
	G	Enable opportunities for remote education and training	Provide and encourage other groups to give online training to equip individuals with adequate competitive skills needed for the wide variety of positions available within the global scientific community.
	H	Encourage funding agencies to provide funding opportunities for the research communities facing hardship	Encourage funding agencies, nonprofits, government foundations, and academic institutions to establish new research funding opportunities for international collaborations, prioritizing joint research and academic projects and programs.

Remote research collaborations might involve sharing data, coauthoring papers, or providing expertise in a particular field (Table 3, group 1, A–D). These strategies can help support scientific research in their country and provide opportunities for researchers to work together and share expertise. Due to the rapid advancements in computer technology, researchers from various fields can be involved in multi-institutional research and contribute remotely, which has proven to be effective [24]. The most straightforward way to do it is for the individual scientists to look up and invite colleagues from their field who work in the country under attack to engage in research or initiate new scientist projects that can be submitted to various international journals (Table 3, group 1, A). This would have an immediate and personal impact, and the connectivity of science in Ukraine will increase from submitting joint peer-reviewed publications in English to the press and providing a much-needed endorsement and strengthening of local science.

If there are no common interests in collaboration, there are still other venues to help support science. For example, if funds are available, they can be donated directly to research institutions to support their work inside the country (Table 3, group 1, B). This might include science organizations, public and private universities, research institutes, and nongovernmental organizations that support science. If there are no monetary funds, sharing resources can also be effective. For example, sharing access to scientific journals, databases, online tools, or software can prove essential for research activities in regions of crisis. Help can also be provided by sharing news and opportunities related to their research on social media or other online platforms such as #ScienceForUkraine and others (Table 3, group 1, C) that can help find potential collaborators, mentors, and students; raise awareness of their work; and connect them with potential funding opportunities.

Finally, the global scientific community is encouraged to be more proactive in registering and participating in events that are held inside Ukraine (Table 3, group 1, D). Some scientific organizations and universities in the country have been putting together webinars, online conferences, and workshops that encourage remote participation by international colleagues. For example, soon after the invasion, the Institute of Molecular Biology and Genetics, at the National Academy of Science (NAS) of Ukraine of Ukraine, organized an “All-Ukrainian Conference on Molecular and Cell Biology with International Participation” (June 2022).

Online events like this provide a great opportunity to learn about current research and connect with potential collaborators in the country and provide a much-needed endorsement and strengthening of local science. Online conferences have many advantages, such as being more accessible to a wider range of participants and reducing travel costs. At the same, there have been several issues, including the loss of informal networking opportunities, the difficulty in maintaining attention during online presentations, and the potential for technical problems [25]. These can be avoided in hybrid-style conferences but must be addressed to maximize the positive impact on scientists at risk who can only attend virtually.

Researchers at risk can also be helped indirectly by modifying the policies of scientific publishers, research foundations, and academic institutions (Table 3, group 2, E–H). For example, scientific journals could support Ukrainian researchers by waiving publication fees and helping with translation and editing. Inviting Ukrainian colleagues onto editorial boards has the effect of increasing the engagement of scientists in Ukraine (Table 3, group 2, E). Some journals have offered a special issue based on the works of Ukrainian members [26]. Journals could allow multilingual support by adding translated versions of their publications in the native languages of the authors [27]. PubMed Central already has a policy enabling acceptance of non-English articles and/or English articles with non-English parts based on the agreements between the publisher and the National Library of Medicine (NLM) at the National Institutes of Health [28].

Organizations and societies that hold scientific conferences can waive fees for scientists participating virtually to allow researchers who cannot leave their country to participate. Many scientific conferences are already hosted in a mixed mode, allowing in-person as well as virtual attendance. This helps break administrative barriers and is well suited for scientists not able to leave their country [29]. Scientific societies can help by waiving the registration fee for academics and researchers so they can participate in international scientific life without leaving the country (Table 3, group 2, F). While writing this article, we approached organizers of several international conferences to ease access for the Ukrainian scientists this year. As a result, the European Society of Human Genetics has decided to waive the 2023 membership fee for all Ukrainian geneticists wishing to join the European Society of Human Genetics who have a professional life focus in Ukraine or have been forced to leave Ukraine due to the war. The Society of Molecular Biology and Evolution developed a hybrid arrangement to encourage scientists who cannot attend the conference in person to apply and will consider waiving the registration fee (in person or online) to scientists unable to pay it. The International Society of Computational Biology is offering a reduced registration fee to the lowest level for online participation in the joint Annual International Conference on Intelligent Systems for Molecular Biology and European Conference on Computational Biology (ISMB/ECCB) 2023 conference to Ukrainian scientists who remain in Ukraine or have been forced to leave Ukraine due to the war.

Training (group 2, G) is another valuable tool to ensure scientific communities in the affected countries are more engaged [30]. There are groups in Ukraine that are eager to participate and be incorporated in remote scientific research collaborations with minimal training. For instance, because of its well-developed education system, Ukraine has a significant number of computer science undergraduates. These students and recent graduates are well suited for remote work and computational research, but normally, after graduating college, they would skip graduate school to pursue the well-paid outsourced jobs in the various industries attracted by significant financial incentives offered by the recruiting companies. In the new reality created by the ongoing invasion, these skilled informaticians are facing limited options in the industry. However, since graduate school provides a waiver of military mobilization, students have started flocking to graduate careers. This has generated a pool of potential researchers who cannot leave the country, have the required skills, and are willing to collaborate on scientific projects remotely. Training this particular group could, for instance, enable the creation of a bioinformatics research community that can be later engaged in international collaborations [30]. However, training requires funding, and we urge funding agencies to enable funding initiatives to train scientists in bioinformatics skills that will allow them to remotely engage in large international consortiums and establish collaboration with the global community. Lack of language skills can erect additional barriers, as not all scientists are proficient in English.

Funding needs to be allocated to encourage and educate scholars who require these abilities to participate in international collaborative opportunities (Table 3, group 2, H). The most flexible and scalable mechanism is to offer remote training and paid remote opportunities for the scientists as well as find appropriate funding to support such opportunities. Online databases have addressed some of these issues, but many remaining issues are hindering their effectiveness. Particularly, because many of these positions are general advertisements for positions available and not specifically geared toward Ukrainian scholars at risk, candidates must compete with other applicants who are not disadvantaged by the war. Even if students and researchers are interested in these opportunities, they need to navigate the specifics of education systems in different respective countries. Due to the combination of political, economic, historical, and cultural reasons, many of the Ukrainian researchers who could benefit from these opportunities cannot compete for them, as they lack the skills necessary to find jobs in science outside their country and often simply do not satisfy the requirements of available job openings. Unless the jobs are specifically created, they would need additional training just to be competitive. To address these issues, we suggest that a platform needs to be developed that could match scientists and computer experts with laboratories and faculties willing to offer remote mentorships or employment [31].

During the COVID-19 pandemic, we all learned that remote learning is possible and effective if the educators are creative and the students are willing. Educators who have developed online resources could team up with educators in crisis zones to co-teach courses assisted by technology. The session could meet synchronously or asynchronously (recording sessions to account for power outages or internet service lags), and both teachers could teach different portions of the course. Students could even collaborate on projects, work could be divided based on the availability of resources, and while some students could do lab work, others might focus on data analysis. Together they could write reports or scientific articles, fostering the next generation of international collaborations. Training also requires teaching materials, and some initiatives like OER (Open Education Resources) can provide them. Historically, OER has been more aimed at younger students, but their community has been working to expand the current limitations (Table 1, OEG Connect).

The global scientific community and decision-making authorities urgently need to implement a coordinated assistance plan targeted to scientists at risk who remain in the invaded country. Some of the authors of this publication created an interdisciplinary group consisting of researchers from countries, including Ukraine, the United States, the United Kingdom, and Germany, and others who share concerns about the fragile situation of the scientific community in these wartime countries and argue about the need to develop effective and feasible mechanisms to support researchers and students at risk along with voices around the world. Governments and the private sector need to establish new research funding opportunities for international collaborations, including joint research and academic projects and programs [31]. An early example of such efforts includes Action Steps for Rebuilding Ukraine's Science, Research, and Innovation by the Academy of US Poland and Ukraine [32], which mentioned the need for remote opportunities but did not provide details on how to enable such opportunities. Another example of a decision-making authority is the US Agency for International Development and the United Nations Educational, Scientific and Cultural Organization, which provide civilian aid and promote social and economic development and international cooperation. But despite these organizations being present worldwide, and even though many individual labs and faculty across the globe are willing to mentor and involve scientists in existing projects, they lack the financial means and mechanisms to support and engage them.

## Effective Mechanisms of Remote Support for Scientists at Risk

As science in the world is becoming more interconnected, scientists in countries at risk from war, political strife, natural disasters, ecological crisis, or economic hardship can continue functioning as part of the international community and be incorporated into the research process remotely. Thanks to the new capabilities in collaborations, they can continue working together across borders and disciplines and help global efforts to tackle global challenges and advance our understanding of the world.

The lessons learned from the current crisis in Ukraine precipitated by the unprovoked Russian invasion can be readily applied to other similar situations. Using Ukraine as an example, we have established several possible venues of assistance to scientific communities facing hardship. This, of course, is not limited to Ukraine but can be applied to other situations. Unfortunately, there are too many examples of similar situations, some resulting from the war in Ukraine; others from persecution in Venezuela, Afghanistan, and Syria; and natural disasters, including Hurricanes Maria and Fiona in Puerto Rico and the recent earthquakes in Turkey and Syria. In this article, we outlined several effective and feasible possible venues to support scientists and students in countries facing hardship or disadvantaged by a military, a political-economic, or an ecological crisis (Table 2). The list of opportunities is by no means exhaustive and is meant to begin, not conclude, the conversation on this issue.

Creating online resources like #ScienceForUkraine was an immediate and successful example of what can be applicable and immediately adapted to political strife and war zones and to areas where natural disasters have occurred. Many opportunities listed on #ScienceForUkraine and similar boards are not specifically created to recruit Ukrainians; these platforms are used to incorporate Ukrainian audiences to recruit for existing positions. With that caveat, its overall effect was positive, because researchers who are looking for opportunities are exposed to a wide variety of options. However, the best scenario is to establish new research funding opportunities for international collaborations, including joint research and academic projects and programs.

There is an urgent need to launch a discussion about the feasible long-term plan to rebuild science and create research opportunities. Programs and mechanisms should be created to allow scientists who cannot leave their home countries to still acquire novel skills remotely, and these skills can then be disseminated to the local scientific community of their designated country. For the researchers who remain in the country, transitioning to computational data-driven research can be a good solution, as many skills can be taught remotely and accessed from anywhere in the world. Systematic training that is needed in state-of-the-art analytical skills can be achieved by developing plans that can include establishing collaboration with world-leading institutions. Remote work is used not only for data-driven research familiar to the reader of this journal but also for the most of humanities and social sciences as well. Remote mechanisms should be created, allowing world-class specialists to teach and train undergraduate and graduate students [32]. The engagement of university leadership to make remote arrangements possible is important, but this is still not widely adopted, and funding agencies should provide specific funding initiatives to support these areas. Integrating new domains of research, which are not relying on physical infrastructure but instead are based on computational research, can result in a shorter recovery time for rebuilding Ukrainian science compared to the restoration of physical infrastructure.

## Supplementary Material

giad045_GIGA-D-23-00120_Original_Submission

giad045_GIGA-D-23-00120_Revision_1

giad045_GIGA-D-23-00120_Revision_2

giad045_Response_to_Reviewer_Comments_Original_Submission

giad045_Response_to_Reviewer_Comments_Revision_1

giad045_Reviewer_1_Report_Original_Submission Anita Bandrowski -- 5/29/2023 Reviewed

giad045_Ukrainian_translation_Scientists_without_borders

## Data Availability

Opportunities for Ukrainian scientists in 1 year since the start of the conflict (February 2022–February 2023) are publicly available in the #ScienceForUkraine database (https://scienceforukraine.eu/listings)..

## Additional File

**giad045_Ukrainian_translation_Scientists_without_borders:** Ukrainian translation of the final manuscript.

## Editor's Note

To make this Review more accessible to readers, the authors have translated the final version of this manuscript into Ukrainian. This file is available as a Supplemental File, giad045_Ukrainian_translation_Scientists_without_borders.
