# Correction to: Scientists without borders: lessons from Ukraine

**DOI:** 10.1093/gigascience/giad066

**Published:** 2023-08-09

This is a correction to: Walter Wolfsberger and others, Scientists without borders: lessons from Ukraine, *GigaScience*, Volume 12, 2023, giad045, https://doi.org/10.1093/gigascience/giad045.

In the originally published version of this manuscript, affiliations for authors Serghei Mangul and Taras K. Oleksyk were inadvertently swapped.

The correct affiliations for Serghei Mangul are:

- Department of Clinical Pharmacy, USC Alfred E. Mann School of Pharmacy and Pharmaceutical Sciences, University of Southern California, Los Angeles, CA 90033, USA
- Department of Computational Biology, University of Southern California, Los Angeles, CA 90033, USA

The correct affiliations for Taras K. Oleksyk are:

- Department of Biological Sciences, Oakland University, Rochester, MI 48309-4479, USA
- Department of Biology, Uzhhorod National University, Uzhhorod 88000, Ukraine

In addition to these changes, the Supplementary file name for the Ukrainian translation of the article has been clarified and references updated in the text.

These errors, for which the publisher apologizes, have been corrected.

