# Supplementary material for: Scientists without borders: lessons from Ukraine
Source: Gigascience. 2023 Jul 27;12:giad045. doi: 10.1093/gigascience/giad045 (PMC10372202; doi:10.1093/gigascience/giad045)
Supplement: giad045_GIGA-D-23-00120_Revision_2 [file giad045_giga-d-23-00120_revision_2.pdf]

GigaScience

Scientists without Borders: Lessons from Ukraine

--Manuscript Draft--

**Manuscript Number:**	GIGA-D-23-00120R2
**Full Title:**	Scientists without Borders: Lessons from Ukraine
**Article Type:**	Review
**Funding Information:**	
**Abstract:**	Conflicts and natural disasters affect entire populations of the countries involved, and, in addition to the thousands of lives destroyed, have a substantial negative impact on the scientific advances these countries provide. The unprovoked invasion of Ukraine by Russia, the devastating earthquake in Turkey and Syria, and the ongoing conflicts in the Middle East are just a few examples. Millions of people have been killed or displaced; their futures uncertain. These events have resulted in extensive infrastructure collapse, with loss of electricity, transportation, and access to services. Schools, universities, and research centers have been destroyed along with decades worth of data, samples, and findings. Scholars in disaster areas face short- and long-term problems in terms of what they can accomplish now for obtaining grants, and for employment in the long run. In our interconnected world, conflicts and disasters are no longer a local problem, but have wide-ranging impacts on the entire world, both now and in the future. Here, we focus on the current and ongoing impact of war on the scientific community within Ukraine, and from this draw lessons that can be applied to all affected countries where scientists at risk are facing hardship. We present and classify examples of effective and feasible mechanisms used to support researchers in countries facing hardship and discuss how these can be implemented with help from the international scientific community and what more is desperately needed. Reaching out, providing accessible training opportunities, and developing collaborations, should increase inclusion and connectivity, support scientific advancements within affected communities, and expedite postwar and disaster recovery.
**Corresponding Author:**	Taras K Oleksyk, Ph.D.<br>Oakland University<br>Rochester, MI UNITED STATES
**Corresponding Author Secondary Information:**	
**Corresponding Author's Institution:**	Oakland University
**Corresponding Author's Secondary Institution:**	
**First Author:**	Walter W Wolfsberger
**First Author Secondary Information:**	
**Order of Authors:**	Walter W Wolfsberger
	Karishma Chhugani
	Khrystyna K Shchubelka, MD/PhD
	Alina Frolova
	Yuriy Salyha, Ph.D.
	Oksana Zlenko, Ph.D.
	Mykhailo Arych
	Dmytro Dzyuba, MD/PhD
	Andrii Parkhomenko
	Volodymyr Smolanka, MD/PhD
	Zeynep H Gümüş, Ph.D.

Powered by Editorial Manager® and ProduXion Manager® from Aries Systems Corporation

	Efe Sezgin, Ph.D.
	Alondra Diaz-Lameiro, Ph.D.
	Viktor Toth, Ph.D.
	Patricia Morton
	Megi Maci
	Eric Bortz
	Fyodor Kondrashov, Ph.D.
	Paweł Łabaj, Ph.D.
	Veronica Romero
	Jakub Hlávka, Ph.D.
	Serghei Mangul, Ph.D.
	Taras K Oleksyk, Ph.D.
**Order of Authors Secondary Information:**	
**Response to Reviewers:**	Dear Dr. Nogoy<br><br>Thank you for accepting our manuscript.<br><br>These sections have been added directly into the new draft of the manuscript.<br><br>Declarations<br><br>Data Availability: Opportunities for Ukrainian scientists in one year since the start of the conflict (February 2022-February 2023) are publicly available in the #ScienceForUkraine database.<br><br>Competing interests: The authors declare that they have no competing interests.<br><br>Funding: All authors volunteered their time. No funding supported work on this article.<br><br>New Software: There was no new software application associated with this article.<br><br>Author contributions: TKO, SM, KC, WWW and YS came up with the conceptual idea; WWW and KC performed the data curation; WWW and KC performed the Formal analysis; TKO, SM KC and WWW developed methodology; TKO and SM coordinated and supervised the collaboration; WWW and TKO visualized the data; All authors contributed to the original draft, review and editing. All authors read and approved the final manuscript.<br><br>I hope you will find these answers satisfactory<br><br>Taras Oleksyk
**Additional Information:**	
**Question**	**Response**
Are you submitting this manuscript to a special series or article collection?	No
**Experimental design and statistics**<br><br>Full details of the experimental design and statistical methods used should be given in the Methods section, as detailed in our	Yes

Powered by Editorial Manager® and ProduXion Manager® from Aries Systems Corporation

Minimum Standards Reporting Checklist. Information essential to interpreting the data presented should be made available in the figure legends.<br><br>Have you included all the information requested in your manuscript?	
**Resources**<br><br>A description of all resources used, including antibodies, cell lines, animals and software tools, with enough information to allow them to be uniquely identified, should be included in the Methods section. Authors are strongly encouraged to cite Research Resource Identifiers (RRIDs) for antibodies, model organisms and tools, where possible.<br><br>Have you included the information requested as detailed in our Minimum Standards Reporting Checklist?	No
If not, please give reasons for any omissions below.<br><br>as follow-up to "**Resources**<br><br>A description of all resources used, including antibodies, cell lines, animals and software tools, with enough information to allow them to be uniquely identified, should be included in the Methods section. Authors are strongly encouraged to cite Research Resource Identifiers (RRIDs) for antibodies, model organisms and tools, where possible.<br><br>Have you included the information requested as detailed in our Minimum Standards Reporting Checklist?<br><br>"	This is a review articles. There is no Methods section. However, we present descriptive data and explain the summary statistics in the text.
**Availability of data and materials**	Yes

All datasets and code on which the conclusions of the paper rely must be either included in your submission or deposited in publicly available repositories (where available and ethically appropriate), referencing such data using a unique identifier in the references and in the "Availability of Data and Materials" section of your manuscript.<br><br>Have you have met the above requirement as detailed in our Minimum Standards Reporting Checklist?	

R2 Revised Manuscript

Click here to access/download;Manuscript;Ukraine Manuscript.2023.TKO22.docx

Confidential: do not distribute.

Review

Scientists without Borders: Lessons from Ukraine

Walter Wolfsberger+
Department of Biological Sciences, Oakland University, Dodge Hall Rm 367, 118 Library Dr Rochester, MI 48309-4479
wwolfsberger@oakland.edu
https://orcid.org/0000-0003-0980-645X

Karishma Chhugani+
Department of Pharmacology and Pharmaceutical Sciences, School of Pharmacy, University of Southern California, Los Angeles, CA, USA
chhugani@usc.edu
https://orcid.org/0000-0001-8764-9352

Khrystyna Shchubelka
Department of Biological Sciences, Oakland University, Dodge Hall Rm 367, 118 Library Dr Rochester, MI 48309-4479, USA
kshchubelka@oakland.edu
https://orcid.org/0000-0002-7630-3257

Alina Frolova
Institute of Molecular Biology and Genetics of National Academy of Sciences of Ukraine, Kyiv Academic University, Kyiv, Ukraine
fshodan@gmail.com
https://orcid.org/0000-0001-5469-7366

Yuriy Salyha
Institute of animal biology NAAS, Vasyl Stus street, 38,
Lviv, 79034, Ukraine
yursalyha@yahoo.com
https://orcid.org/0000-0002-5731-7936

Oksana Zlenko
National Scientific Center "Institute of Experimental and Clinical Veterinary Medicine", Kharkiv, Ukraine
oksana.ceratium@gmail.com
https://orcid.org/0000-0002-4357-0762

Mykhailo Arych
Institute of Economics and Management,
National University of Food Technologies
68, Volodymyrska St., 01601
Kyiv, Ukraine
mykhailo.arych@gmail.com

https://orcid.org/0000-0002-0910-2332

Dmytro Dziuba
MD, Professor, Department of Anesthesiology and Intensive Care,
P.L. Shpyk NUHC Ukraine, Dorogozhytska Str.,
Kyiv Ukraine.
dr_dzuba@ukr.net
https://orcid.org/0000-0002-9979-8889

Andrii Parkhomenko
University of Southern California, Marshall School of Business, Department of Finance and Business Economics,
Los Angeles, CA 90089
parkhome@usc.edu
https://orcid.org/0000-0002-6699-6790

Volodymyr Smolanka
Uzhhorod National University, 32, Voloshyna Street,
Uzhhorod Ukraine 88000
v.smolanka@uzhnu.edu.ua
https://orcid.org/0000-0001-7296-8297

Zeynep H. Gümüş
Department of Genetics and Genomic Sciences
Icahn School of Medicine at Mount Sinai
New York, NY 10029, USA
zeynep.gumus@mssm.edu

Efe Sezgin
Department of Food Engineering,
Izmir Institute of Technology,
Urla, Izmir, Turkey
efeszgn0@gmail.com
https://orcid.org/0000-0002-8000-7485

Alondra Diaz-Lameiro
Department of Biology,
University of Puerto Rico at Mayaguez
Mayaguez, Puerto Rico 00681
alondra.diaz@upr.edu
https://orcid.org/0000-0001-7842-0855

Viktor R. Toth
Aquatic Botany and Microbial Ecology Research Group
Balaton Limnological Research Institute,
Klebelsberg Kuno utca 3.

Tihany, Hungary 8237
toth.viktor@blki.hu
https://orcid.org/0000-0002-6182-2759

Megi Maci
Loyola University Chicago,
Stritch School of Medicine, 2160 S 1st Ave,
Maywood, IL 60153
mmaci1130@gmail.com
https://orcid.org/0000-0003-2907-0793

Eric Bortz
Department of Biological Sciences, University of Alaska Anchorage,
3211 Providence Dr,
Anchorage AK 99508
ebortz@alaska.edu
orcid.org/0000-0002-1376-0012

Fyodor Kondrashov
Institute of Science and Technology Austria,
1 Am Campus,
Klosterneuburg, 3400, Austria
fyodor.kondrashov@ist.ac.at
https://orcid.org/0000-0001-8243-4694

Patricia Morton
Department of Sociology, Department of Public Health
Wayne State University,
Detroit MI, USA
mortonp@wayne.edu
https://orcid.org/0000-0003-1437-7063

Paweł P. Łabaj
Małopolska Centre of Biotechnology,
Jagiellonian University, Gronostajowa 7A,
Kraków, Poland
pawel.labaj@gmail.com
https://orcid.org/0000-0002-4994-0234

Veronika Romero
Department of Neurobiology,
University of Utah, 20 S 2030 E, BPRB 456,
Salt Lake City, UT 84112, USA
nika.biph@gmail.com
https://orcid.org/0000-0001-6720-8592

Jakub Hlávka
University of Southern California, Price School of Public Policy, 635 Downey Way, Verna & Peter Dauterive Hall (VPD),
Los Angeles, CA 90089-3333, USA
jakub.hlavka@usc.edu
https://orcid.org/0000-0002-2766-390X

Serghei Mangul*
Department of Clinical Pharmacy, School of Pharmacy, University of Southern California, 1540 Alcazar Street,
Department of Quantitative and Computational Biology, USC Dornsife College of Letters, Arts and Sciences University of Southern California
Los Angeles, CA 90033, USA
serghei.mangul@gmail.com
https://orcid.org/0000-0003-4770-3443

Taras K. Oleksyk*
Department of Biological Sciences, Oakland University, Dodge Hall Rm 367, 118 Library Dr Rochester, MI 48309-4479, USA
Department of Biology,
Uzhhorod National University, 32, Voloshyna Street,
Uzhhorod, 88000 Ukraine
oleksyk@oakland.edu
https://orcid.org/0000-0002-8148-3918
[+] These authors contributed equally to this work.
[*] These authors jointly supervised this work.

Corresponding authors:

Taras K. Oleksyk,
Department of Biological Sciences, Oakland University
Dodge Hall Rm 367, 118 Library Dr., Rochester, MI 48309-4479
Oleksyk@oakland.edu

Serghei Mangul*
Department of Clinical Pharmacy, School of Pharmacy, University of Southern California, 1540 Alcazar Street, Los Angeles, CA 90033, USA
Department of Quantitative and Computational Biology,
USC Dornsife College of Letters, Arts and Sciences University of Southern California
serghei.mangul@gmail.com

Abstract

Conflicts and natural disasters affect entire populations of the countries involved, and, in addition to the thousands of lives destroyed, have a substantial negative impact on the scientific advances these countries provide. The unprovoked invasion of Ukraine by Russia, the devastating earthquake in Turkey and Syria, and the ongoing conflicts in the Middle East are just a few examples. Millions of people have been killed or displaced; their futures uncertain. These events have resulted in extensive infrastructure collapse, with loss of electricity, transportation, and access to services. Schools, universities, and research centers have been destroyed along with decades worth of data, samples, and findings. Scholars in disaster areas face short- and long-term problems in terms of what they can accomplish now for obtaining grants, and for employment in the long run. In our interconnected world, conflicts and disasters are no longer a local problem, but have wide-ranging impacts on the entire world, both now and in the future. Here, we focus on the current and ongoing impact of war on the scientific community within Ukraine, and from this draw lessons that can be applied to all affected countries where scientists at risk are facing hardship. We present and classify examples of effective and feasible mechanisms used to support researchers in countries facing hardship and discuss how these can be implemented with help from the international scientific community and what more is desperately needed. Reaching out, providing accessible training opportunities, and developing collaborations, should increase inclusion and connectivity, support scientific advancements within affected communities, and expedite postwar and disaster recovery.

Keywords

Conflicts; Scholars; Europe; Ukraine; Russia; Science; Funding; Remote learning; Scholarship opportunities; Bioinformatics;

Background

Countries all over the world face a variety of hardships, from political upheaval, wars, economic crises, and natural disasters, where [1]millions of people's lives are lost or destroyed and require a huge amount of planning, aid, and resources to rebuild. For this review, we want to focus on how to aid scholars and students who have become displaced, disconnected from their professional communities, and face an unknown future. In the face of disaster, established researchers lose funding, workplaces, laboratories—including samples and data which may never be recovered, lines of communication, and collaboration. Students, undergraduate as well as graduate, have their careers interrupted, and entire generations of talented youth become disengaged, disconnect from educational opportunities, and drop out of their research careers. Groups facing such crises can turn for help to their colleagues in the international community, and with every success and failure of these efforts, we can learn how to face the next global challenge.

There is value in diverse scientific communities, if we strive to have inclusion, equity, and diverse perspective in the scientific community, joining hands with scientists in crises is the first important step. Placing resources, time, and effort into our struggling colleagues, follows a great scientific tradition of providing opportunities to capable minds, even in tough situations. Stories such as Einstein's, Tesla's, and Curie's, all of which escaped war and/or persecution, are evidence that humanity can benefit in great ways from international scientific collaborations. With the scientific world has become increasingly interconnected, the capability of the international community to aid researchers with hardships is potentially much easier than would have been possible before investment and engagement in worldwide projects. Collaborative cooperation, such as that seen for the Global Alliance for Genomics and Health [2]**,** the International Brain Initiative [3], the Human Genome Project [4], and the Large Hadron Collider [5] emerge every day to address global scientific challenges. Landmark projects like these have been hugely successful because they allowed the exchange of resources, expertise, and data on a global scale. The combined knowledge of collaborative groups is widely shared, discussed, and disseminated through scientific conferences (in-person, hybrid, and remote) and open publishing. Online preprints, journals, and open-access publishing have revolutionized the distribution of knowledge by allowing easier information access. Global research networks emerged across many fields, and initiatives like the Open Access and Open Data movements [6] further promoted knowledge sharing. Finally, UNESCO's Recommendation on Open Science [7] is a valuable tool for supporting scientists in hardship areas, that can be implemented by providing funding for open science projects, training and support for scientists on open science tools and practices, and developing open science infrastructure to ensure they have the resources and support needed to carry out their work and contribute to scientific progress.

Global research networks are growing rapidly to help scientists connect and collaborate, and technology is playing a key role in this process. With the increasing availability of high-speed internet and digital tools, scientists from around the world are now able to connect and collaborate in real time, regardless of their location. Web-based technologies behind collaborative scientific projects require less in-person engagement than a decade ago. This is especially true in the emerging omics sciences (i.e. genomics, transcriptomics, proteomics, and bioinformatics) [8]. If early on, the biggest bottlenecks in the Human Genome Project and 1,000 Genomes Project were in data acquisition [9], where international collaborations required specific laboratory skills and on-site resources for sample collection and sequencing. Today, medical and other phenotypic data

can be easily accumulated and shared between researchers and their institutions digitally, and loads of low-cost DNA, RNA, and protein sequencing data from research and high-throughput screening are becoming available, and can be jointly analyzed by international groups engaged in genomics and bioinformatics research [10–13]**).** The major bottleneck facing these groups in the decades after the publication has been the lack of access to powerful hardware and software to analyze torrents of incoming data. Now, this issue can be resolved with advanced clusters, increased storage, and cloud computing. However, a collaboration that relies on building large international networks can only grow by maximizing global human engagement [14].

Online platforms and social media help create and connect networks that transcend international borders, where researchers can share ideas, collaborate on projects, and engage in discussions with colleagues from different countries and backgrounds, while the emerging cloud computing, data visualization, and artificial intelligence tools lead to more efficient research coordination and faster discoveries. These technologies are breaking down barriers and creating opportunities for scientists to connect in ways that were not possible just a few years ago and are helping to accelerate scientific progress around the world. We believe that providing opportunities to groups of scientists temporarily disconnected as a result of war, or isolated as a result of political, economic, or ecological crisis is critical to global science. In this review, we tried to identify the most effective mechanisms to support researchers in countries facing hardship using a case study based on the activities carried out to help researchers during the year of conflict in Ukraine [1]

The global response of the scientific community to the war in Ukraine

More than a year of war in Ukraine is only one example of the scientific community responding to a major crisis. Immediately after the start of the invasion on February 24, 2022, several online resources were created by volunteers who were concerned with the negative impact on the work of their colleagues. These were mostly open spreadsheets listing short and longer-term positions, internships, and other types of support that were communicated [15] (**Table 1**). Some of these resources were spontaneous, originating from concerned individuals and groups. In one example, an *ad hoc* group from the University of Oregon posted a list of laboratories willing to support displaced Ukrainian scientists, quickly gained in popularity, and 40 days after its inception featured more than 2,000 listings for positions in Europe and the USA (Labs Supporting Ukrainian Scientists, **Table 1**). A similar list was also created at Sam Houston University (Ukrainian Scholar Placement Database, **Table 1**). Following these, a larger collaborative initiative, *#ScienceForUkraine* [16] emerged just two days after the war began as an online collaboration of hundreds of international scholars volunteering to build a central database of international opportunities available to Ukrainian researchers. As the war progressed, several other initiatives emerged intending to support Ukrainian scholars, including the Ukrainian Scholar Placement Database developed by Sam Houston University (**Table 1**), which compiled a list of funding programs available to researchers in Ukraine. The activity of the emerging groups was not limited to research opportunities. Simultaneously, other communities such as *Saving Ukrainian Cultural Heritage Online* (SUCHO, **Table 1**), engaged in web-archiving, digitizing, and preserving heritage from Ukrainian cultural institutions (See the full list in **Table 1**). Appeals were made to support those who were already studying abroad but could no longer continue their programs [17].

Table 1. Links to resources for Ukrainian scientists that have been mentioned in this article. This list is not exhaustive as new opportunities are published every day. For a more complete representation, please search databases such as *#ScienceforUkraine* and others mentioned below.

Resource	Description	Location	Link
#ScienceforUkraine	A community group collecting and disseminating information about support opportunities.	Inter-national	https://scienceforukraine.eu
Council for At-Risk Academics (CARA)	An NGO collaboration of UK universities to provide relief of suffering and the defense of learning and science	The UK	https://www.cara.ngo
ERA4Ukraine	An EU initiative support researchers of Ukraine by providing them with an overview of all existing actions at European and national levels	The EU	https://euraxess.ec.europa.eu/ukraine
European Federation of Academies of Sciences and Humanities (ALLEA)	European fund for displaced scientists	The EU	https://allea.org/european-fund-for-displaced-scientists
ERC4Ukraine	European Research Council (ERC) initiative provide temporary employment to refugee researchers and support staff	The EU	https://erc.europa.eu/apply-grant/erc-ukraine
The Guild	The Guild of European Research-Intensive Universities Association support initiative	The EU	https://www.the-guild.eu/resources/the-guild-s-universities-supporting-researchers-ac.html
IIE Scholar Rescue Fund	A global program that arranges funds and supports fellowships for threatened and displaced scholars	The EU	https://www.scholarrescuefund.org
Labs Supporting Ukrainian Scientists	An online database to help find positions for displaced scholars from Ukraine at the University of Oregon	USA	https://tinyurl.com/yanb37ck
Le Collège de France	A fund featuring state-sponsored opportunities for displaced scientists	France	https://www.college-de-france.fr/
OEG Connect	An open education resource to connect, share, and work together to make learning accessible	Inter-national	https://connect.oeglobal.org/
Philipp Schwartz Initiative of the Humboldt Foundation	A program to help researchers subject to significant personal threat in their country to continue their work at German universities	Germany	https://www.humboldt-foundation.de
MSCA4Ukraine	A subsidiary of the EU's Marie Skłodowska-Curie Actions, to provide fellowship support to researchers from Ukraine	The EU	https://sareurope.eu/msca4ukraine/

Saving Ukrainian Cultural Heritage Online (SUCHO)	A group of volunteers to web-archive digitize and preserve heritage from Ukrainian cultural institutions (no research support)	Inter-national	https://www.sucho.org
Scholars at Risk (SAR)	International network to protect threatened scholars and promote academic freedom around the world. Cooperating with several national science foundations in Europe	Inter-national	https://www.scholarsatrisk.org
Scientists and Engineers in Exile or Displaced (SEED) program	A collaboration of National Academies to award grants	USA and Poland	https://www.nationalacademies.org/our-work/scientists-and-engineers-in-exile-or-displaced-seed-program
Shevchenko Emergency Fund	A fund to support scholars by Shevchenko Scientific Society the largest and the oldest Ukrainian scientific organization outside of Ukraine	USA	https://shevchenko.org
Swiss National Science Foundation	A state-sponsored opportunities for displaced scientists	Switzer land	https://www.snf.ch/en
The Association for Slavic, East European & Eurasian Studies	Resource database to help scholars from Ukraine	USA	https://www.aseees.org/resources/help-displaced-scholars-ukraine
The UK-Ukraine Twinning Initiative	An institution-to-institution collaboration model to lay the foundations of strong partnerships between the UK and Ukrainian universities supported by grants from UKRI's Research England	UK and Ukraine	https://www.twinningukraine.com/
Ukrainian Global University Initiative	A consortium of leading Ukrainian educational institutions and organizations to support Ukrainian students scholars with the opportunities for quality education and research	Ukraine	https://uglobal.university
Ukrainian Scholar Placement Database	An online database to help find positions for displaced scholars from Ukraine at Sam Houston State University	USA	https://tinyurl.com/2f8csafa

Existing cooperation networks that were in place before the war were used to rapidly launch or amplify the support measures. A group of initiatives was rising from existing pre-war structures, notably national societies, and academies. For example, the Office of Policy and Global Affairs, a Division of the US National Academies previously engaged in science diplomacy among different nations, in collaboration with the Polish Academy of Sciences quickly created the *Scientists and Engineers in Exile or Displaced initiative* to support several hundreds of Ukrainian scholars with short-term stipends for studies outside of Ukraine (PAS-NAS SEED, **Table 1**). The publicly-funded *Shevchenko Scientific Society* (the largest and the oldest Ukrainian scientific organization outside of Ukraine founded in 1873) started to create opportunities for the displaced scientists supported by its US and Canada-based members. Most of the national academies in the EU block issued supportive statements to Ukraine, and within weeks, the *European Federation of Academies of Sciences and Humanities* (ALLEA, **Table 1**) using a donation from the Breakthrough Prize Foundation started hosting scholars displaced by the war. After millions of

refugees crossed the EU border and settled in the various European countries, more opportunities were created by national organizations such as the *Swiss National Science Foundation, the Polish Academy of Sciences, and Le Collège de France*. The European Commission opened the *European Research Area portal* for Ukraine, and *Horizon Europe*, with its complementary *Euratom Research and Training Programme*, offered free participation to support the Ukrainian scientific community. The *MSCA4Ukraine* consortium funded under the EU's Marie Skłodowska-Curie Actions, provides fellowship support for hundreds of displaced researchers from Ukraine.

However, national academies were only one source of support. More active were global research networks, and discipline-specific international associations. International organizations, such as *Scholars at Risk* (SAR, **Table 1**), formed as far back as 1999 to protect threatened scholars and promote academic freedom around the world, and others (*IIE Scholar Rescue Fund, Philipp Schwartz Initiative of the Humboldt Foundation in Germany, Council for At-Risk Academics in the UK*, and others (**Table 1**)) backed by governments, philanthropists and large corporate sponsors rapidly focused their efforts and started creating opportunities for the displaced Ukrainians as well. The UK-based twinning initiative, an institution-to-institution collaboration model, incorporated the UK-Ukraine Research and Innovation twinning grants from UKRI's Research England to enable twins to further their research and innovation collaborations. In a relatively short time, individual universities, research institutes, and other academic organizations from all across the globe started offering scholarships, paid positions, and other types of support on their own. Volunteer initiatives and the unwavering support received from an array of universities, academic institutions, scientific societies, publishers, and funding agencies worldwide were met with great gratitude from the Ukrainian scientific community [18]. This unprecedented widespread support will be crucial on the path to recovery and mitigate the consequences of the war for academic and scientific communities and their institutions in the country. The networks established early at the start of the war laid a foundation for the further integration of Ukrainian scientists into the global community and will become grounds for the future success of more formal organizational initiatives involving Ukraine that are being developed at this time (i.e. *Ukrainian Global University Initiative, Horizon Europe*, etc.).

Despite these opportunities, so far only a fraction of scholars could physically relocate abroad and take advantage of the opportunities offered to them by the global response, the rest remained in Ukraine. This is a complex issue. According to the rules of martial law in Ukraine, males between the ages of 18 and 60 cannot leave the country. Many academics joined the Territorial Defense or were drafted into the Ukrainian Armed Forces [19]. Some did not want to leave their families and homes in Ukraine, for a short research opportunity abroad, or needed to take care of their children and the elderly who could not come along. Even among those who could leave, many were discouraged by the bureaucratic process of obtaining long-term visas.

Those staying in Ukraine found themselves in the most unstable and vulnerable situation. Many universities, especially those in the east of the country are either closed or relocated to safer areas. The budgets of the state-run universities were cut to save funds for the war effort. While the base salaries for teaching are usually maintained, these were traditionally already very low and commonly supplemented by income from funded research and overtime work, which are now dropped [20]. The national research programs have been plagued by the issues associated with the war, and the funding has mostly been cut off or frozen. Despite the dire situation, many researchers in Ukraine continued to maintain their academic and research activities.

The Ukrainian crisis has several distinct challenges, and it invoked a complex response with many underlying factors, including historical, cultural, economic, and political factors. The initial reaction of the scientific community was to provide opportunities for refugees fleeing the country. Some of the recent examples resulted in many scholars seeking refuge. Under the current political regime, about 2,000 highly skilled Venezuelan scientists responsible for the publication of a third of all scientific papers from Venezuela and representing 15 percent of the Venezuelan research community, have left their academic posts and fled abroad [21]. The recent crisis associated with the withdrawal of US troops from Afghanistan forced many researchers fleeing and those who remained lost funding and faced a threat of persecution [22]. The Syrian civil war has been ongoing since 2011 and has caused significant humanitarian, economic, and political challenges producing science refugees: academics and scientists that required help outside their country [23]. Initially, it seemed that the war in Ukraine would produce a similar result, and the research community immediately began preparing for the influx of researchers who needed a place to live and work outside of their country.

Analysis of the opportunities for Ukrainian scientists listed in the *#ScienceForUkraine* database

To understand what types of opportunities have been offered to Ukrainian scientists during the first year of the war, we surveyed and classified the entries posted on the *#ScienceForUkraine* website one year after the start of the invasion (February 2022 - February 2023). The goal of this initiative was to help provide support for the Ukrainian academic community and also streamline any setbacks for the Ukrainian scientific community[16]. While this database may be incomplete, and some entries have been deleted (personal communication), it is still the largest publicly available database that can be surveyed for the types of opportunities that have been offered.

A review of the *#ScienceForUkraine* database shows a clear trend towards in-person and a lack of remote opportunities (**Table 2, Figure 1**). *#ScienceForUkraine* volunteers tried to achieve this by raising awareness among research communities across the world about the situation, posting about new opportunities for students and scholars to receive opportunities across the world, and working with various European funding organizations to help support the academic community. We used the default classification of this database into "*paid positions*", "*educational*", "*funding programs*" and "*partnerships*" **(Figure 1A)**. The vast majority of the 1,871 listed opportunities listed were "*paid positions*". Opportunities labeled as "*educational*" and "*funding programs*" were distributed in similar proportions (**Table 2, Figure 1A**).

Overall, all classes of opportunities were distributed roughly equally between "*researchers*" and "*students*" of every level, with very few indicated as "*professional*" (**Figure 1.B**). It is difficult to classify these into distinct sub-categories, but generally, they included research and postdoctoral fellowships, as well as some researcher jobs. Unsurprisingly, most of them were located in Europe (n=1,293), with North America (the US and Canada) as a distant second (n=181). Germany had the most paid positions, followed by France, Poland, Canada, and the United States (**Figure 1.C**). Germany also had the most opportunities in education as well as funding programs (**Figure 1. D,E**).

Generally, since most of these initiatives were trying to deal with the refugee crisis at hand, their success relied largely on the readiness of the Ukrainian scientists to move abroad. The review of the #ScienceForUkraine database demonstrates that most entries require travel and shows the relative scarcity of remote opportunities for scholars that cannot leave the country during the time

Confidential: do not distribute.

of war: only 52 positions (out of 1,871 available), specifically mentioned the word "remote" and most of them suggest a possibility of relocation. Despite the vital importance of initiatives offering temporary or permanent positions, they target opportunities for only a fraction of the scientists who left or were able to leave the country. As a result, very often the existing opportunities may go unfulfilled which would frustrate those who offer them, as well as those who could not use them.

Table 2. Locations of opportunities for Ukrainian scientists in the *#ScienceForUkraine* database.

Type	All Available	Europe	North America	Asia	South America	Oceania	Africa
**Paid Positions**	1,456 (83%)	1,293	181	44	11	12	2
**Education Programs**	170 (9%)	145	12	8	0	2	0
**Funding Programs**	67 (4%)	53	8	1	1	0	0
**Partnerships**	6 (<1%)	1	2	0	1	1	0
**Other Help**	77 (4%)	68	5	0	0	0	0
**Unspecified**	5 (<5%)	3	0	1	1	0	0
**Total**		1,563	208	54	14	15	2

Figure 1. Locations of opportunities for Ukrainian scientists in the *#ScienceForUkraine* database in one year since the start of the conflict (February 2022-February 2023).

A. Number of opportunities by program type. The majority of the 1,871 listed opportunities can be classified as paid positions. These include research and postdoctoral fellowships, as well as some researcher jobs. Most of these are in Europe (1,293), with North America as a distant second (181). Educational opportunities and funding programs were distributed in similar proportions.
B. Number of opportunities by applicant qualification. Opportunities were distributed roughly equally between researchers and students of every level, with very few indicated as "*professional*".
C, D, and E. Distribution of different types of opportunities (listed in **Fig. 1.A**) by country. The total number of opportunities per category is given in parentheses. Germany had the most opportunities in all categories.

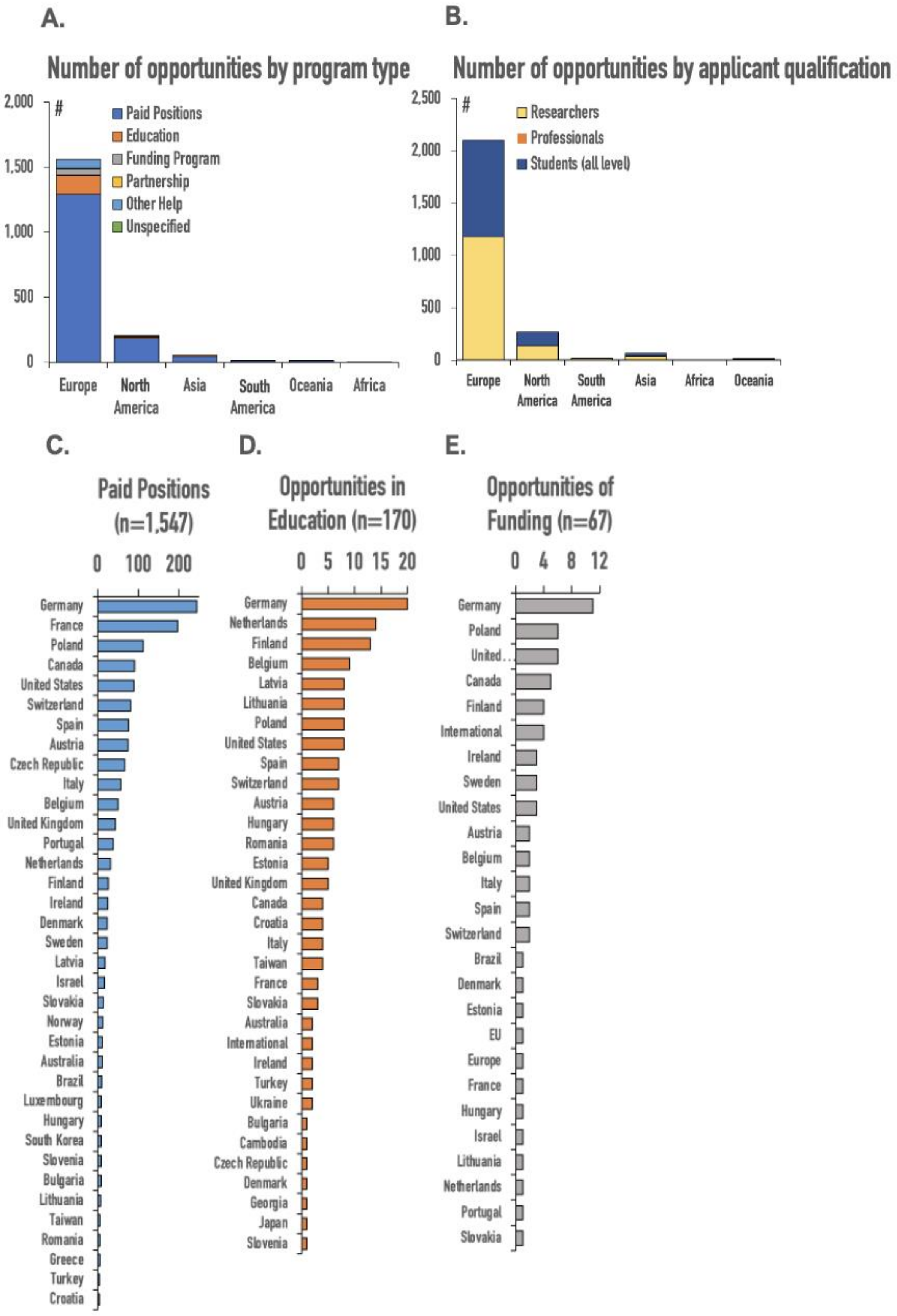
A.
Number of opportunities by program type
#
Paid Positions
Education
Funding Program
Partnership
Other Help
Unspecified
0
500
1,000
1,500
2,000
Europe
North America
Asia
South America
Oceania
Africa
B.
Number of opportunities by applicant qualification
#
Researchers
Professionals
Students (all level)
0
500
1,000
1,500
2,000
2,500
Europe
North America
South America
Asia
Africa
Oceania
C.
Paid Positions (n=1,547)
0 100 200
Germany
France
Poland
Canada
United States
Switzerland
Spain
Austria
Czech Republic
Italy
Belgium
United Kingdom
Portugal
Netherlands
Finland
Ireland
Denmark
Sweden
Latvia
Israel
Slovakia
Norway
Estonia
Australia
Brazil
Luxembourg
Hungary
South Korea
Slovenia
Bulgaria
Lithuania
Taiwan
Romania
Greece
Turkey
Croatia
D.
Opportunities in Education (n=170)
0 5 10 15 20
Germany
Netherlands
Finland
Belgium
Latvia
Lithuania
Poland
United States
Spain
Switzerland
Austria
Hungary
Romania
Estonia
United Kingdom
Canada
Croatia
Italy
Taiwan
France
Slovakia
Australia
International
Ireland
Turkey
Ukraine
Bulgaria
Cambodia
Czech Republic
Denmark
Georgia
Japan
Slovenia
E.
Opportunities of Funding (n=67)
0 4 8 12
Germany
Poland
United...
Canada
Finland
International
Ireland
Sweden
United States
Austria
Belgium
Italy
Spain
Switzerland
Brazil
Denmark
Estonia
EU
Europe
France
Hungary
Israel
Lithuania
Netherlands
Portugal
Slovakia

Confidential: do not distribute.

Effective and feasible mechanisms to support scientists and students from Ukraine

We hope to use the ongoing struggle in Ukraine to initiate the conversation about effective and feasible mechanisms for the support of scholars and students at risk. There is a need for discussion on the feasibility and efficacy of various approaches to short-term and long-term strategies that help scholars during the active phase of conflicts and post-war reconstruction periods. Specifically, we want to bring the attention of the international community to creating scalable mechanisms that can be offered to several thousands of scientists who have left their respective countries, versus those who are remaining in the country. It would only make sense to complement the opportunities that require travel with the ones that can be more suitable for the scientists staying in Ukraine: offering a wider range of remote positions, engaging the scientific community in the global community, and offering effective training to address any existing knowledge gaps [1]. This combined strategy would help Ukrainian science to endure difficult times, secure their development, and help further integrate them into the international scientific community. This will also set an example for other scientific communities at risk. We use Ukraine as an example from our collaborations, but the recommendations herein can be used to support research initiatives of scientists in other countries.

The effective remote opportunities described here could be sorted into two groups (**Table 2**). In the first group, there are efforts that are directed towards and imply establishing contact with a specific scientist or a group of scientists in Ukraine (**Table 2, A-D**). In the second group, there are venues to help Ukrainian researchers indirectly, without knowing or contacting specific researchers or scientific groups (**Table 2, E-H**).

Table 2. Mechanisms to support scientists and students from countries experiencing hardship. Group 1 (A-D) includes efforts that are directed towards and imply establishing contact with a specific scientist or a group of scientists in Ukraine. Group 2 (E-H) In the second group, there are venues to help Ukrainian researchers indirectly, without knowing or contacting specific researchers or scientific groups.

	#	Mechanism	Description
**Group 1**	**A**	**Establish direct remote research collaborations**	A scientist or researcher in can collaborate remotely with Ukrainian scientists directly on common research projects.
	**B**	**Donate funds and provide access to resources**	Funds can be donated directly to research institutions to support their work
	**C**	**Share research opportunities**	Informing colleagues about opportunities that could be missed because of the limited access or language barriers.
	**D**	**Participate in online events inside the country**	Taking part in webinars, online conferences, and workshops put together by local organizers
**Group 2**	**E**	**Promote publications in the scientific journals**	Encouraging journals to support researchers by waving publication fees, and providing translations and editorial services, and including versions of published papers in authors native languages.
	**F**	**Promote participation in international conferences**	Encourage organizers of scientific conferences to provide free or discounted access to registration and memberships.
	**G**	**Enabling opportunities for remote education and training**	Provide and encourage other groups to provide online training to provide have adequate competitive for the multitude of the positions offered by the global scientific community.
	**H**	**Encourage funding agencies to provide funding opportunities for the** research **communities facing hardship**	Encourage funding agencies, non-profits, foundations governmental, and academic institutions to establish new research funding opportunities for international collaborations, prioritizing joint research and academic projects and programs.

Confidential: do not distribute.

Remote research collaborations might involve sharing data, co-authoring papers, or providing expertise in a particular field (**Table 2, Group 1, A-D**). These strategies can help support scientific research in their country and provide opportunities for researchers to work together and share expertise. Due to the rapid advancements in computer technology, researchers from various fields can be involved in multi-institutional research and contribute remotely which has proven to be effective [24]. The most straightforward way to do it is for the individual scientists to look up and invite colleagues from their field who work in the country under attack to engage in research or initiate new scientist projects which can be submitted to various international journals (**Table 2, Group 1, A**). This would have an immediate and personal impact, and the connectivity of science in Ukraine will increase from submitting joint peer-reviewed publications in English to the press and providing a much-needed endorsement and strengthening of local science.

If there are no common interests in collaboration, there are still other venues to help support science. For example, if funds are available, they can be donated directly to research institutions to support their work inside the country (**Table 2, Group 1, B**). This might include science organizations, public and private universities, research institutes, and non-governmental organizations that support science. If there are no monetary funds, sharing resources can also be an effective help. For example, sharing access to scientific journals, databases, online tools, or software can prove essential for research activities in regions of crisis. Help can also be provided by sharing news and opportunities related to their research on social media or other online platforms such as *#ScienceForUkraine* and others (**Table 2, Group 1, C**) that can help find potential collaborators, mentors, and students, raise awareness of their work and connect them with potential funding opportunities.

Finally, the global scientific community is encouraged to be more proactive in registering and participating in events that are held inside Ukraine (**Table 2, Group 1, D**). Some scientific organizations and universities in the country have been putting together webinars, online conferences, and workshops that encouraged remote participation by international colleagues. For example, soon after the invasion, the Institute of Molecular Biology and Genetics, at the NAS of Ukraine organized an "*All-Ukrainian Conference on Molecular and Cell Biology with international participation*" (June 2022).

Online events like this provide a great opportunity to learn about current research and connect with potential collaborators in the country and provide a much-needed endorsement and strengthening of local science. Online conferences have many advantages, such as being more accessible to a wider range of participants and reducing travel costs. At the same, there were several issues, including the loss of informal networking opportunities, the difficulty in maintaining attention during online presentations, and the potential for technical problems [25]. These can be avoided in hybrid-style conferences but must be addressed to maximize the positive impact on scientists at risk that can only attend virtually.

Researchers at risk can also be helped indirectly by modifying the policies of scientific publishers, research foundations, and academic institutions (**Table 2, Group 2, E-H**). For example, scientific journals could support Ukrainian researchers by waiving publication fees and helping with translation and editing. Inviting Ukrainian colleagues onto editorial boards has the effect of increasing the engagement of scientists in Ukraine (**Table 2, Group 2, E**). Some journals have

offered a special issue based on the works of Ukrainian members [26]. Journals could allow multilingual support by adding translated versions of their publications in the native languages of the authors [27]. PubMed Central already has a policy enabling accept non-English articles and/or English articles with non-English parts based on the agreements between the publisher and NLM [28].

Organizations and societies that hold scientific conferences can waive fees for scientists participating virtually to allow researchers that cannot leave their country to participate. Many scientific conferences are already hosted in a mixed mode allowing in-person as well as virtual attendance. This helps break administrative barriers and this is well suited for scientists not able to leave their country [29]. Scientific societies can help by waiving the registration fee for academics and researchers so they can participate in international scientific life without leaving the country (**Table 2, Group 2, F**). While writing this article we approached organizers of several international conferences to ease access for the Ukrainian scientists this year. As a result, *The European Society of Human Genetics* (ESHG) has decided to waive the 2023 membership fee for all Ukrainian geneticists wishing to join ESHG that have a professional life focus in Ukraine or have been forced to leave Ukraine due to the war. *The Society of Molecular Biology and Evolutio*n (SMBE) developed a hybrid arrangement to encourage scientists who cannot attend the conference in person to apply and will consider waiving registration fees (in person or online) to scientists because of their inability to pay it. *The International Society of Computational Biology* (ISCB) is offering a reduced registration fee to the lowest level for online participation in ISMB/ECCB 2023 Conference, to Ukrainian scientists that remain in Ukraine or have been forced to leave Ukraine due to the war.

Training (**Group 2, G**) is another valuable tool to ensure scientific communities in the affected countries are more engaged. [30]There are groups in Ukraine that are eager to participate and be incorporated in remote scientific research collaborations with minimal training. For instance, because of its well-developed education system, Ukraine has a significant number of Computer Science undergraduates. These students and recent graduates are well suited for remote work and computational research but normally, after graduating college, they would skip graduate school to pursue the well-paid outsourced jobs in the various industries attracted by significant financial incentives offered by the recruiting companies. In the new reality created by the ongoing invasion, these skilled informaticians are facing limited options in the industry. However, since graduate school provides a waiver of military mobilization, they started flocking to graduate careers. This generated a pool of potential researchers who cannot leave the country, have the required skills, and are willing to collaborate on scientific projects remotely. Training this particular group could for instance enable the creation of a bioinformatics research community that can be later engaged in international collaborations[30]. However, training requires funding, and we urge funding agencies to enable funding initiatives to train scientists in bioinformatics skills which will allow them to remotely engage in large international consortiums and establish collaboration with the global community. Lack of language skills can erect additional barriers, as not all scientists are proficient in English.

Funding needs to be allocated to encourage and educate scholars who require these abilities to participate in international collaborative opportunities (**Table 2, Group 2, H**). The most flexible and scalable mechanism is to offer remote training and paid remote opportunities for the scientists as well as find appropriate funding to support such opportunities. Online databases addressed

some of these issues, but many remaining issues are hindering their effectiveness. Particularly, because many of these positions are general advertisements for positions available, and not specifically geared toward Ukrainian scholars at risk, candidates must compete with other applicants that are not disadvantaged by the war. Even if students and researchers are interested in these opportunities, they need to navigate the specifics of education systems in different respective countries. Due to the combination of political, economic, historical, and cultural reasons, many of the Ukrainian researchers that could benefit from these opportunities, cannot compete for them, as they lack the skills necessary to find jobs in science outside their country, and often simply do not satisfy the requirements of available job openings. Unless the jobs are specifically created, they would need additional training just to be competitive. To address these issues, we suggest that a platform needs to be developed that could match scientists and computer experts with laboratories and faculties willing to offer remote mentorships or employment. [31]

During the COVID-19 pandemic, we all learned that remote learning is possible and effective if the educators are creative and the students are willing. Educators that have developed online resources, could team up with educators in crisis zones to co-teach courses assisted by technology. The session could meet synchronously or asynchronously (recording sessions to account for power outages or internet service lags), and both teachers could teach different portions of the course. Students could even collaborate on projects, work could be divided based on the availability of resources, and while some students could do lab work, others might focus on data analysis. Together they could write reports or scientific articles, fostering the next generation of international collaborations. Training also requires teaching materials, and some initiatives like OER (Open Education Resources) can provide them. Historically, OER has been more aimed at younger students but their community has been working to expand the current limitations (**Table 1,** *OEG Connect)*.

The global scientific community and decision-making authorities urgently need to implement a coordinated assistance plan targeted to scientists at risk that are remaining in the invaded country. Some of the authors of this publication created an interdisciplinary group consisting of researchers from countries, including Ukraine, the United States, the UK, Germany, and others who share concerns about the fragile situation of the scientific community in these wartime countries and argue about the need to develop effective and feasible mechanisms to support researchers and students at risk along with voices around the world. Governments and the private sector need to establish new research funding opportunities for international collaborations, including joint research and academic projects and programs [31]. An early example of such efforts include Action Steps for Rebuilding Ukraine's Science, Research, and Innovation by the Academy of US Poland and Ukraine [32] and they mentioned the need for remote opportunities but did not provide details on how to enable such opportunities. Another example of a decision-making authority is the United States Agency for International Development (USAID) or The United Nations Educational, Scientific and Cultural Organization (UNESCO) which provide civilian aid and promote social and economic development and international cooperation. But despite these organizations being present worldwide, and even though many individual labs and faculty across the globe are willing to mentor and involve scientists in existing projects, they lack the financial means and mechanisms to support and engage them.

Effective Mechanisms of remote support scientists at risk

As science in the world is becoming more interconnected, scientists in countries at risk from war, political strife, natural disasters, ecological crisis, or economic hardship can continue functioning as part of the international community and can be incorporated into the research process remotely. Thanks to the new capabilities in collaborations, they can continue working together across borders and disciplines and help global efforts to tackle global challenges and advance our understanding of the world.

The lessons learned from the current crisis in Ukraine precipitated by the unprovoked Russian invasion can be readily applied to other similar situations. Using Ukraine as an example, we have established several possible venues of assistance to scientific communities facing hardship. This, of course, is not limited to Ukraine but can be applied to other situations. Unfortunately, there are too many examples of similar situations, some resulting from the war in Ukraine, others from persecution in Venezuela, Afghanistan, and Syria, and natural disasters starting from Hurricanes Maria and Fiona in Puerto Rico to the recent earthquakes in Turkey and Syria. In this paper, we outlined several effective and feasible possible venues to support scientists and students in countries facing hardship, or disadvantaged by the military, political economic, or ecological crisis (**Table 2**). The list of opportunities is by no means exhaustive and is meant to begin, not conclude the conversation on this issue.

Creating online resources like *#SceinceForUkraine* was an immediate and successful example of what can be applicable and immediately adapted to political strife and war zones, and to areas where natural disasters have occurred. Many opportunities listed on *#ScienceForUkraine* and similar boards are not specifically created to recruit Ukrainians, these platforms are used to incorporate Ukrainian audiences to recruit for existing positions. With that caveat, its overall effect was positive, because researchers that are looking for opportunities are exposed to a wide variety of options. However, the best scenario is to establish new research funding opportunities for international collaborations, including joint research and academic projects and programs.

There is an urgent need to launch a discussion about the feasible long-term plan to rebuild science and create research opportunities. Programs and mechanisms should be created to allow scientists that cannot leave their home countries can still acquire novel skills remotely, and these skills can then be disseminated to the local scientific community of their designated country. For the researchers that remain in the country, transitioning to computational data-driven research can be a good solution, as many skills can be taught remotely and accessed from anywhere in the world. Systematic training that is needed in state-of-the-art analytical skills can be achieved by developing plans which can include establishing collaboration with world-leading institutions. Remote work is used not only for data-driven research familiar to the reader of this journal but also for the most of humanities and social sciences as well. Remote mechanisms should be created allowing world-class specialists to teach and train undergraduate and graduate students [32]. The engagement of university leadership to make remote arrangements possible is important, however, this is still not widely adopted, and funding agencies should provide specific funding initiatives to support these areas. Integrating new domains of research, which are not relying on physical infrastructure but instead are based on computational research, can result in a shorter recovery time for rebuilding Ukrainian science compared to the restoration of physical infrastructure.

Declarations

Data Availability: Opportunities for Ukrainian scientists in one year since the start of the conflict (February 2022-February 2023) are publicly available in the #ScienceForUkraine database.

Competing interests: The authors declare that they have no competing interests.

Funding: All authors volunteered their time. No funding supported work on this article.

New Software: There was no new software application associated with this article.

Author contributions: TKO, SM, KC, WWW and YS came up with the conceptual idea; WWW and KC performed the data curation; WWW and KC performed the Formal analysis; TKO, SM KC and WWW developed methodology; TKO and SM coordinated and supervised the collaboration; WWW and TKO visualized the data; All authors contributed to the original draft, review and editing. All authors read and approved the final manuscript.

Acknowledgments: Our paper is dedicated to all freedom-loving people around the world, and to the people of Ukraine who fight for our freedom. Special thanks to Anita Bandrowski, Oleksandra V. Ivashchenko and Sanita Reinsone for the helpful review, valuable criticism, and useful suggestions while preparing this manuscript, and to Tetiana Yes'kova for helping with Ukrainian translation.

References

[1] K. Chhugani, K., A. Frolova, Y. Salyha, A. Fiscutean, O. Zlenko, S. Reinsone, W.W. Wolfsberger, O. v. Ivashchenko, M. Maci, D. Dziuba, A. Parkhomenko, E. Bortz, F. Kondrashov, P.P. Łabaj, V. Romero, J. Hlávka, T.K. Oleksyk, S. Mangul, Remote opportunities for scholars in Ukraine, Science (1979). 378 (2022) 1285–1286. https://doi.org/10.1126/science.adg0797.

[2] THE GLOBAL ALLIANCE FOR GENOMICS AND HEALTH, A federated ecosystem for sharing genomic, clinical data, Science (1979). 352 (2016) 1278–1280. https://doi.org/10.1126/science.aaf6162.

[3] R. Yuste, C. Bargmann, Toward a Global BRAIN Initiative, Cell. 168 (2017) 956–959. https://doi.org/10.1016/j.cell.2017.02.023.

[4] R.A. Gibbs, The Human Genome Project changed everything, Nat Rev Genet. 21 (2020) 575–576. https://doi.org/10.1038/s41576-020-0275-3.

[5] L. Horyn, The Large Hadron Collider, in A Search for Displaced Leptons in the ATLAS Detector, Springer Nature Switzerland AG, 2022: pp. 21–29. https://doi.org/10.1007/978-3-030-91672-5_3.

[6] F. Miedema, Open Science: the Very Idea, Springer Netherlands, Dordrecht, 2022. https://doi.org/10.1007/978-94-024-2115-6.

[7] UNESCO, UNESCO Recommendation on Open Science, UNESCO Headquarters (Paris) - UNESCO Archives (Paris), Paris, 2021.

[8] D. Cirillo, A. Valencia, Big data analytics for personalized medicine, Curr Opin Biotechnol. 58 (2019) 161–167. https://doi.org/10.1016/j.copbio.2019.03.004.

[9] J. Li, H. Chen, Y. Wang, M.-J.M. Chen, H. Liang, Next-Generation Analytics for Omics Data, Cancer Cell. 39 (2021) 3–6. https://doi.org/10.1016/j.ccell.2020.09.002.
[10] P.S. Hosmani, T. Shippy, S. Miller, J.B. Benoit, M. Munoz-Torres, M. Flores-Gonzalez, L.A. Mueller, H. Wiersma-Koch, T. D'Elia, S.J. Brown, S. Saha, A quick guide for student-driven community genome annotation, PLoS Comput Biol. 15 (2019) e1006682. https://doi.org/10.1371/journal.pcbi.1006682.
[11] E.F. Ryder, W.R. Morgan, M. Sierk, S.S. Donovan, S.D. Robertson, H.C. Orndorf, A.G. Rosenwald, E.W. Triplett, E. Dinsdale, M.A. Pauley, W.E. Tapprich, Incubators: Building community networks and developing open educational resources to integrate bioinformatics into life science education, Biochemistry, and Molecular Biology Education. 48 (2020) 381–390. https://doi.org/10.1002/bmb.21387.
[12] G. Cantelli, G. Cochrane, C. Brooksbank, E. McDonagh, P. Flicek, J. McEntyre, E. Birney, R. Apweiler, The European Bioinformatics Institute: empowering cooperation in response to a global health crisis, Nucleic Acids Res. 49 (2021) D29–D37. https://doi.org/10.1093/nar/gkaa1077.
[13] T.K. Oleksyk, W.W. Wolfsberger, K. Shchubelka, S. Mangul, S.J. O'Brien, The Pioneer Advantage: Filling the blank spots on the map of genome diversity in Europe, Gigascience. 11 (2022). https://doi.org/10.1093/gigascience/giac081.
[14] N. v. Fedoroff, Science Diplomacy in the 21st Century, Cell. 136 (2009) 9–11. https://doi.org/10.1016/j.cell.2008.12.030.
[15] M. Maryl, O. v. Ivashchenko, M. Reinfelds, S. Reinsone, M.E. Rose, Addressing the needs of Ukrainian scholars at risk, Nat Hum Behav. 6 (2022) 746–747. https://doi.org/10.1038/s41562-022-01387-7.
[16] M. Rose, S. Reinsone, M. Andriushchenko, M. Bartosiak, A. Bobak, L. Drury, M. Düring, I. Figueira, E. Gailīte, I. Gozhyk, L.G. Abreu, I. Gutierrez, O. Ivashchenko, K. van Heuckelom, J. Jaudzema, K. Jurikova, A. Klos, J. Knörzer, E. Kutafina, M. Kwaśnicki, H. Lane, I. Ļaksa-Timinska, B. Laschowski, A. Lattu, M. Maci, K. Mäkinen-Rostedt, M. Maryl, M. van Meerbeek, O. Morin, V. Mosienko, A. Palou Vilar, K. de Pauw, M. Pelepets, M. Reinfelds, C. Rujan, Z. Santybayeva, A. Skatova, M. Vita, I. Weaver, M. Wnuk, R. Beckett, #ScienceForUkraine: an Initiative to Support the Ukrainian Academic Community. "3 Months Since Russia's Invasion in Ukraine", February 26 – May 31, 2022, SSRN Electronic Journal. (2022). https://doi.org/10.2139/ssrn.4139263.
[17] A. Heyman, OU's Ukrainian community ask administration for financial assistance, Oakland Post. (2022). https://oaklandpostonline.com/41529/campus/ous-ukrainian-community-ask-administration-for-financial-assistance/ (accessed March 1, 2023).
[18] A. Klimash, Ukraine's Chemists Persevere Through A Year Of War, Chemistry World. (2023). https://www.chemistryworld.com/opinion/ukraines-chemists-persevere-through-a-year-of-war/4017035.article (accessed April 11, 2023).
[19] N. Gaind, How three Ukrainian scientists are surviving Russia's brutal war, Nature. 605 (2022) 414–416. https://doi.org/10.1038/d41586-022-01272-3.
[20] M. Maryl, M. Jaroszewicz, I. Degtyarova, Y. Polishchuk, M. Pachocka, M. Wnuk, Beyond Resilience: Professional Challenges, Preferences, and Plans of Ukrainian Researchers Abroad, Zenodo. (2022) 4–30. https://doi.org/10.5281/zenodo.7380509 (accessed April 11, 2023).
[21] N. Casey, Hungry Venezuelans Flee in Boats to Escape Economic Collapse, New York Times. (2016). https://www.nytimes.com/2016/11/25/world/americas/hungry-

venezuelans-flee-in-boats-to-escape-economic-collapse.html?smid=url-share (accessed March 1, 2023).
[22] S. Mallapaty, Afghanistan's terrified scientists predict huge research losses, Nature. 597 (2021) 15–16. https://doi.org/10.1038/d41586-021-02353-5.
[23] T.M. Stoepler, J.E. Creswell, N.J. Anthis, F. Chaverneff, R.R. Parkhurst, The Role of Science Diplomacy in International Crises: Syria as a Case Study, Sci Dipl. 6 (2017). https://www.sciencediplomacy.org/article/2017/role-science-diplomacy-in-international-crises-syria-case-study (accessed March 1, 2023).
[24] P. Hunter, Remote working in research, EMBO Rep. 20 (2019). https://doi.org/10.15252/embr.201847435.
[25] E. Levitis, C.D.G. van Praag, R. Gau, S. Heunis, E. DuPre, G. Kiar, K.L. Bottenhorn, T. Glatard, A. Nikolaidis, K.J. Whitaker, M. Mancini, G. Niso, S. Afyouni, E. Alonso-Ortiz, S. Appelhoff, A. Arnatkeviciute, S.M. Atay, T. Auer, G. Baracchini, J.M.M. Bayer, M.J.S. Beauvais, J.D. Bijsterbosch, I.P. Bilgin, S. Bollmann, S. Bollmann, R. Botvinik-Nezer, M.G. Bright, V.D. Calhoun, X. Chen, S. Chopra, H. Chuan-Peng, T.G. Close, S.L. Cookson, R.C. Craddock, A. De La Vega, B. De Leener, D. V Demeter, P. Di Maio, E.W. Dickie, S.B. Eickhoff, O. Esteban, K. Finc, M. Frigo, S. Ganesan, M. Ganz, K.G. Garner, E.A. Garza-Villarreal, G. Gonzalez-Escamilla, R. Goswami, J.D. Griffiths, T. Grootswagers, S. Guay, O. Guest, D.A. Handwerker, P. Herholz, K. Heuer, D.C. Huijser, V. Iacovella, M.J.E. Joseph, A. Karakuzu, D.B. Keator, X. Kobeleva, M. Kumar, A.R. Laird, L.J. Larson-Prior, A. Lautarescu, A. Lazari, J.H. Legarreta, X.-Y. Li, J. Lv, S. Mansour L., D. Meunier, D. Moraczewski, T. Nandi, S.A. Nastase, M. Nau, S. Noble, M. Norgaard, J. Obungoloch, R. Oostenveld, E.R. Orchard, A.L. Pinho, R.A. Poldrack, A. Qiu, P.R. Raamana, A. Rokem, S. Rutherford, M. Sharan, T.B. Shaw, W.T. Syeda, M.M. Testerman, R. Toro, S.L. Valk, S. Van Den Bossche, G. Varoquaux, F. Váša, M. Veldsman, J. Vohryzek, A.S. Wagner, R.J. Walsh, T. White, F.-T. Wong, X. Xie, C.-G. Yan, Y.-F. Yang, Y. Yee, G.E. Zanitti, A.E. Van Gulick, E. Duff, C. Maumet, Centering inclusivity in the design of online conferences—An OHBM–Open Science perspective, Gigascience. 10 (2021). https://doi.org/10.1093/gigascience/giab051.
[26] A.O. Cherninskyi, Global Excellence in Cellular Neuropathology: Ukraine, Frontiers in Cellular Neuroscience, 2023. https://www.frontiersin.org/research-topics/46571/global-excellence-in-cellular-neuropathology-ukraine (accessed March 1, 2023).
[27] A.J. Majeske, A.J. Mercado Capote, A. Komissarov, A. Bogdanova, N. v. Schizas, S.O. Castro Márquez, K. Hilkert, W. Wolfsberger, T.K. Oleksyk, The first complete mitochondrial genome of *Diadema antillarum* (Diadematoida, Diadematidae), GigaByte. 2022 (2022) 1–12. https://doi.org/10.46471/gigabyte.73.
[28] PMC, PubMed Central Tagging Guidelines, NCBI. (2023). https://www.ncbi.nlm.nih.gov/pmc/pmcdoc/tagging-guidelines/article/style.html (accessed April 12, 2023).
[29] J. Wu, A. Rajesh, Y.-N. Huang, K. Chhugani, R. Acharya, K. Peng, R.D. Johnson, A. Fiscutean, C.D. Robles-Espinoza, F.M. de La Vega, R. Bao, S. Mangul, Virtual meetings promise to eliminate geographical and administrative barriers and increase accessibility, diversity and inclusivity, Nat Biotechnol. 40 (2022) 133–137. https://doi.org/10.1038/s41587-021-01176-z.
[30] S. Mangul, L.S. Martin, B. Langmead, J.E. Sanchez-Galan, I. Toma, F. Hormozdiari, P. Pevzner, E. Eskin, How bioinformatics and open data can boost basic science in countries

and universities with limited resources, Nat Biotechnol. 37 (2019) 324–326. https://doi.org/10.1038/s41587-019-0053-y.
[31] International Science Council, Amplifying the voices of at-risk, displaced and refugee scientists, (2021).
[32] National Academies of Sciences Engineering and Medicine, Action Steps for Rebuilding Ukraine’s Science, Research, and Innovation, (2022). https://www.nationalacademies.org/news/2022/06/action-steps-for-rebuilding-ukraines-science-research-and-innovation (accessed March 1, 2023).

OAKLAND UNIVERSITY
Biological Sciences

May 31, 2023

To: Nicole Nogoy, Ph.D
GigaScience Editorial Office
www.gigasciencejournal.com

Dear Dr. Nogoy,

Thank you for considering our manuscript "*Scientists without Borders: Lessons from Ukraine*" (GIGA-D-23-00120) for publication in GigaScience. We appreciate the time and effort the reviewers have taken to evaluate our work, and we are grateful for the constructive feedback we have received.

We are pleased to hear that the manuscript is potentially acceptable for publication following some essential revisions. In this revised submission, we included a point-by-point response to the reviewers' comments, detailing the modifications we have made, and attached them at the end of this letter.

In addition, we tried to ensure that our revised manuscript adheres to the journal's style. We have also gone through the manuscript to further correct all the inconsistencies and attached an additional copy where the changes are tracked between the earlier and the revised version. We are confident that these changes will strengthen the manuscript.

Thank you once again for the opportunity to improve our manuscript. We believe that the revisions we are making will substantially enhance the quality and impact of our work.

Sincerely yours

Taras K. Oleksyk, Ph.D.
Assistant Professor
Department of Biological Sciences
Oakland University
oleksyk@oakland.edu

OAKLAND UNIVERSITY

Biological Sciences

Reviewer 1:
(Revisions are in blue)

Page 9
Polish Academy of Sciences quickly created the Scientists and
Engineers in Exile or Displaced initiative to support several hundred Ukrainian scholars with short term stipends for studies outside of Ukraine (PAS-NAS SEED, Table 1).

Corrected: Polish Academy of Sciences quickly created the Scientists and Engineers in Exile or Displaced initiative to support several hundred Ukrainian scholars with short-term stipends for studies outside of Ukraine (PAS-NAS SEED, Table 1).

Page 15
At the same time, there several issues, including the loss of informal networking opportunities, the difficulty of maintaining attention during online presentations, and the potential for technical problems [25].

Corrected: At the same time, there are several issues, including the loss of informal networking opportunities, the difficulty of maintaining attention during online presentations, and the potential for technical problems [25].

Page 16
Particularly, because these positions are not really geared toward Ukrainian scholars, candidates must compete with other applicants that are not disadvantaged by the war.

Corrected: Particularly, because many of these positions are general advertisements for positions available, and not specifically geared toward Ukrainian scholars at risk, candidates must compete with other applicants that are not disadvantaged by the war.

Page 17
we suggest that a platform needs to be developed that could match scientists and computer experts with laboratories and faculty

Corrected: we suggest that a platform needs to be developed that could match scientists and computer experts with laboratories and faculties willing to offer remote mentorships or employment.

Page 17
work could be divided based on availability of resources, some students could do lab work, while others might focus on data analysis

Corrected: work could be divided based on the availability of resources, some students could do lab work, while others might focus on data analysis

Page 17
created an interdisciplinary group consisting of researchers

College of Arts and Sciences

375 Dodge Hall | 118 Library Drive, Rochester, Michigan 48309-4479
(248) 370-3550 | Fax: (248) 370-4225 | oakland.edu/biology

from countries, including Ukraine, the United States, UK, Germany, and others

Corrected: created an interdisciplinary group consisting of researchers
from countries, including Ukraine, the United States, the UK, Germany, and others

Page 17
Another example of a decision making authority is the United States Agency for International Development (USAID)

Corrected: Another example of a decision-making authority is the United States Agency for International Development (USAID)

375 Dodge Hall | 118 Library Drive, Rochester, Michigan 48309-4479
(248) 370-3550 | Fax: (248) 370-4225 | oakland.edu/biology

Manuscript with Tracked Changes
Click here to access/download;Personal Cover;Ukraine Manuscript.2023.TKO20.trax.docx
Confidential: do not distribute.

Review

Scientists without Borders: Lessons from Ukraine

Walter Wolfsberger+
Department of Biological Sciences, Oakland University, Dodge Hall Rm 367, 118 Library Dr
Rochester, MI 48309-4479
wwolfsberger@oakland.edu
https://orcid.org/0000-0003-0980-645X

Karishma Chhugani+
Department of Pharmacology and Pharmaceutical Sciences, School of Pharmacy, University of Southern California, Los Angeles, CA, USA
chhugani@usc.edu
https://orcid.org/0000-0001-8764-9352

Khrystyna Shchubelka
Department of Biological Sciences, Oakland University, Dodge Hall Rm 367, 118 Library Dr
Rochester, MI 48309-4479, USA
kshchubelka@oakland.edu
https://orcid.org/0000-0002-7630-3257

Alina Frolova
Institute of Molecular Biology and Genetics of National Academy of Sciences of Ukraine,
Kyiv Academic University, Kyiv, Ukraine
fshodan@gmail.com
https://orcid.org/0000-0001-5469-7366

Yuriy Salyha
Institute of animal biology NAAS, Vasyl Stus street, 38,
Lviv, 79034, Ukraine
yursalyha@yahoo.com
https://orcid.org/0000-0002-5731-7936

Oksana Zlenko
National Scientific Center "Institute of Experimental and Clinical Veterinary Medicine",
Kharkiv, Ukraine
oksana.ceratium@gmail.com
https://orcid.org/0000-0002-4357-0762

Mykhailo Arych
Institute of Economics and Management,
National University of Food Technologies
68, Volodymyrska St., 01601
Kyiv, Ukraine
mykhailo.arych@gmail.com

https://orcid.org/0000-0002-0910-2332

Dmytro Dziuba
MD, Professor, Department of Anesthesiology and Intensive Care,
P.L. Shpyk NUHC Ukraine, Dorogozhytska Str.,
Kyiv Ukraine.
dr_dzuba@ukr.net
https://orcid.org/0000-0002-9979-8889

Andrii Parkhomenko
University of Southern California, Marshall School of Business, Department of Finance and Business Economics,
Los Angeles, CA 90089
parkhome@usc.edu
https://orcid.org/0000-0002-6699-6790

Volodymyr Smolanka
Uzhhorod National University, 32, Voloshyna Street,
Uzhhorod Ukraine 88000
v.smolanka@uzhnu.edu.ua
https://orcid.org/0000-0001-7296-8297

Zeynep H. Gümüş
Department of Genetics and Genomic Sciences
Icahn School of Medicine at Mount Sinai
New York, NY 10029, USA
zeynep.gumus@mssm.edu

Efe Sezgin
Department of Food Engineering,
Izmir Institute of Technology,
Urla, Izmir, Turkey
efeszgn0@gmail.com
https://orcid.org/0000-0002-8000-7485

Alondra Diaz-Lameiro
Department of Biology,
University of Puerto Rico at Mayaguez
Mayaguez, Puerto Rico 00681
alondra.diaz@upr.edu
https://orcid.org/0000-0001-7842-0855

Viktor R. Toth
Aquatic Botany and Microbial Ecology Research Group
Balaton Limnological Research Institute,
Klebelsberg Kuno utca 3.

Tihany, Hungary 8237
toth.viktor@blki.hu
https://orcid.org/0000-0002-6182-2759

Megi Maci
Loyola University Chicago,
Stritch School of Medicine, 2160 S 1st Ave,
Maywood, IL 60153
mmaci1130@gmail.com
https://orcid.org/0000-0003-2907-0793

Eric Bortz
Department of Biological Sciences, University of Alaska Anchorage,
3211 Providence Dr,
Anchorage AK 99508
ebortz@alaska.edu
orcid.org/0000-0002-1376-0012

Fyodor Kondrashov
Institute of Science and Technology Austria,
1 Am Campus,
Klosterneuburg, 3400, Austria
fyodor.kondrashov@ist.ac.at
https://orcid.org/0000-0001-8243-4694

Patricia Morton
Department of Sociology, Department of Public Health
Wayne State University,
Detroit MI, USA
mortonp@wayne.edu
https://orcid.org/0000-0003-1437-7063

Paweł P. Łabaj
Małopolska Centre of Biotechnology,
Jagiellonian University, Gronostajowa 7A,
Kraków, Poland
pawel.labaj@gmail.com
https://orcid.org/0000-0002-4994-0234

Veronika Romero
Department of Neurobiology,
University of Utah, 20 S 2030 E, BPRB 456,
Salt Lake City, UT 84112, USA
nika.biph@gmail.com
https://orcid.org/0000-0001-6720-8592

Jakub Hlávka
University of Southern California, Price School of Public Policy, 635 Downey Way, Verna & Peter Dauterive Hall (VPD),
Los Angeles, CA 90089-3333, USA
jakub.hlavka@usc.edu
https://orcid.org/0000-0002-2766-390X

Serghei Mangul*
Department of Clinical Pharmacy, School of Pharmacy, University of Southern California, 1540 Alcazar Street,
Department of Quantitative and Computational Biology, USC Dornsife College of Letters, Arts and Sciences University of Southern California
Los Angeles, CA 90033, USA
serghei.mangul@gmail.com
https://orcid.org/0000-0003-4770-3443

Taras K. Oleksyk*
Department of Biological Sciences, Oakland University, Dodge Hall Rm 367, 118 Library Dr
Rochester, MI 48309-4479, USA
Department of Biology,
Uzhhorod National University, 32, Voloshyna Street,
Uzhhorod, 88000 Ukraine
oleksyk@oakland.edu
https://orcid.org/0000-0002-8148-3918
[+] These authors contributed equally to this work.
[*] These authors jointly supervised this work.

Corresponding authors:

Taras K. Oleksyk,
Department of Biological Sciences, Oakland University
Dodge Hall Rm 367, 118 Library Dr., Rochester, MI 48309-4479
Oleksyk@oakland.edu

Serghei Mangul*
Department of Clinical Pharmacy, School of Pharmacy, University of Southern California, 1540 Alcazar Street, Los Angeles, CA 90033, USA
Department of Quantitative and Computational Biology,
USC Dornsife College of Letters, Arts and Sciences University of Southern California
serghei.mangul@gmail.com

Abstract

~~Recent~~ Conflicts and natural disasters ~~have affected~~affect entire populations of the countries involved, and, in addition to the thousands of lives destroyed, have a substantial negative impact on the scientific advances these countries provide. The unprovoked invasion of Ukraine by Russia ~~in only~~, the ~~most recent example of such an event.~~ devastating earthquake in Turkey and Syria, and the ongoing conflicts in the Middle East are just a few examples. Millions of people have been killed or displaced ~~from their homes~~; their futures uncertain. ~~The attack~~ These events have resulted in ~~an~~ extensive infrastructure collapse, ~~including~~with loss of electricity, transportation, and access to services ~~including~~. Schools, universities, and research centers~~. With universities~~ have been destroyed~~,~~ along with decades worth of data, samples, and findings~~,~~. Scholars in disaster areas ~~are facing~~face short- and long-term problems in terms of what they can accomplish now ~~as indication of scholarship for~~for obtaining grants, and for employment in the long run. ~~-~~In our interconnected world, conflicts and disasters are no longer a local problem, but have ~~a~~ wide-ranging ~~impact~~impacts on the entire ~~humanity~~world, both now and in the future. ~~In this review~~Here, we focus on the current and ongoing ~~impacts~~impact of war ~~conflict~~ on the scientific community within Ukraine, ~~but~~and from this draw lessons that can be applied to all affected countries where scientists at risk are facing hardship. ~~-~~We present and classify examples of ~~effective~~effective and feasible mechanisms used to support researchers in countries facing hardship and discuss how these can be implemented with help from the international scientific community~~.~~ and what more is desperately needed. Reaching out, providing accessible training opportunities, and developing collaborations, should increase inclusion and connectivity, support scientific advancements within ~~the~~ affected communities, and expedite postwar and disaster recovery.

Keywords

Conflicts; Scholars; Europe; Ukraine; Russia; Science; Funding; Remote learning; Scholarship opportunities; Bioinformatics;

Confidential: do not distribute.

Background

Countries all over the world face a variety of hardships, from political upheaval, wars, economic crises, and natural disasters, where [1]millions of people's lives are lost or destroyed and require a huge amount of planning, aid, and resources to rebuild. For~~ the purposes of~~ this review, we want to focus on how to aid scholars and students who have become displaced, disconnected from their professional communities, and face an unknown future. In the face of disaster, established researchers lose funding, workplaces, laboratories—including samples and data which may never be recovered, lines of communication, and collaboration. Students, undergraduate as well as graduate, have their careers interrupted, and entire generations of talented youth become disengaged, disconnect from educational opportunities, and drop out ~~from~~of their research careers. Groups facing such crises can turn for help to their colleagues in the international community, and with every success and failure of these efforts, we can learn how to face the next global challenge.

There is value in diverse scientific communities, if we strive to have inclusion, equity, and diverse perspective in the scientific community, joining hands with ~~scientist~~scientists in crises is ~~a~~the first important step. Placing resources, time, and effort into our struggling colleagues, follows a great scientific tradition of providing opportunities to capable minds, even in tough situations. Stories such as Einstein's, Tesla's, and Curie's, all of which escaped war and/or persecution, are evidence that humanity can benefit in great ways from international scientific collaborations. With the scientific world ~~having~~has become increasingly interconnected, the capability ~~for~~of the international community to aid researchers with hardships is potentially much easier than would have been possible ~~prior to~~before investment and engagement in worldwide projects. Collaborative cooperation, such as that seen for the Global Alliance for Genomics and Health [2]**,** the International Brain Initiative [3], the Human Genome Project [4~~],~~], and the Large Hadron Collider [5] emerge every day to address global scientific challenges. Landmark projects like these have been hugely successful because they allowed the exchange of resources, expertise, and data on a global scale. The combined knowledge of collaborative groups is widely shared, discussed, and disseminated through scientific conferences (in~~-~~-person, hybrid, and remote) and open publishing. Online preprints, journals, and open-access publishing have revolutionized the distribution of knowledge by allowing easier information access. Global research networks emerged across many fields, and initiatives like the Open Access and Open Data movements [6] further promoted knowledge sharing. Finally,~~ the~~ UNESCO's Recommendation on Open Science [7] is a valuable tool for supporting scientists in hardship areas, that can be implemented by providing funding for open science projects, training and support for scientists on open science tools and practices, and developing open science infrastructure to ensure they have the resources and support needed to carry out their work and contribute to scientific progress.

Global ~~connections~~research networks are growing rapidly to help scientists connect ~~with each other~~and collaborate, and technology is playing a key role in this process. With the increasing availability of high-speed internet and digital tools, scientists from around the world are now able to connect and collaborate ~~with each other~~ in real-~~ ~~time, regardless of their location. Web-based technologies behind~~ the~~ collaborative scientific projects require less in-person engagement than a decade ago. This is especially true in the emerging omics sciences (i.e. genomics, transcriptomics, proteomics, and bioinformatics) [8]. If early on, the biggest bottlenecks in the Human Genome Project and 1,000 Genomes Project were in data acquisition [9], where international collaborations required specific laboratory skills and on-site resources for sample collection and sequencing. Today,

medical and other phenotypic data can be easily accumulated and shared between researchers and their institutions digitally, and loads of low~~-~~-cost DNA, RNA, and protein sequencing data from research and high-throughput screening ~~is~~are becoming available, and can be jointly analyzed by international groups engaged in genomics and bioinformatics research [10–13]**)**. The major bottleneck facing these groups in the decades after the publication ~~of~~ has been the lack of access to powerful hardware and software to analyze torrents of incoming data. Now, this issue can be resolved with ~~the~~ advanced clusters, increased storage, and cloud computing. However, a collaboration that relies on building large international networks can only grow by maximizing ~~the~~ global human engagement [14].

Online platforms and social media help create and connect networks that transcend international borders, where researchers can share ideas, collaborate on projects, and engage in discussions with colleagues from different countries and backgrounds, while the emerging cloud computing, data visualization, and artificial intelligence tools lead to more efficient research coordination and faster discoveries. These technologies are breaking down barriers and creating opportunities for scientists to connect ~~with each other~~ in ways that were not possible just a few years ago~~,~~ and are helping to accelerate scientific progress around the world. We believe that providing opportunities to ~~the~~ groups of scientists temporarily disconnected as a result of war, or isolated as a result of political, economic, or ecological crisis is critical to global science. In this review, we tried to identify the most effective mechanisms to support researchers ~~and~~ in countries facing hardship using a case study ~~of~~ based on the activities carried out to help researchers during the year of conflict in Ukraine [1]

The global response of the scientific community to the war in Ukraine

More than a year of war in Ukraine is only one example of the scientific community responding to a major crisis. Immediately after the start of the invasion on February 24, 2022, several online resources were created by volunteers who were concerned with the negative impact on the work of their colleagues. These were mostly open spreadsheets listing short and longer-term positions, internships, and other types of support that were communicated [15] (**Table 1**). Some of these resources were spontaneous, originating from concerned individuals and groups. In one example, an *ad hoc* group from the University of Oregon posted a list of laboratories willing to support displaced Ukrainian scientists, quickly gained in popularity, and ~~in~~ 40 days ~~since~~after its inception featured more than 2,000 listings for positions in Europe and the USA (Labs Supporting Ukrainian Scientists, **Table 1**). A similar list was also created at Sam Houston University (Ukrainian Scholar Placement Database, **Table 1**). ~~—~~Following these, a larger collaborative initiative, *#ScienceForUkraine* [16] emerged just two days after the war began as an online collaboration of hundreds of international scholars volunteering to build a central database of international opportunities available to Ukrainian researchers. As the war progressed, several other initiatives emerged ~~with the aim of supporting~~intending to support Ukrainian scholars, including the Ukrainian Scholar Placement Database developed by Sam Houston University (**Table 1**), which compiled a list of funding programs available to researchers in Ukraine. The activity of the emerging groups ~~were~~was not limited to research opportunities. Simultaneously, other communities such as *Saving Ukrainian Cultural Heritage Online* (SUCHO, **Table 1**), engaged in web-archiving, digitizing, and preserving heritage from Ukrainian cultural institutions (See the full list in **Table 1**). Appeals were made to support those who were already studying abroad but could no longer continue their programs [17].

Table 1. Links to resources for Ukrainian scientists that have been mentioned in this article. This list is not exhaustive as new opportunities are published every day. For a more complete representation, please search databases such as *#ScienceforUkraine* and others mentioned below.

Resource	Description	Location	Link
#ScienceforUkraine	A community group collecting and disseminating information about support opportunities.	Inter-national	https://scienceforukraine.eu
Council for At-Risk Academics (CARA)	An NGO collaboration of UK universities to provide relief of suffering and the defense of learning and science	The UK	https://www.cara.ngo
ERA4Ukraine	An EU initiative support researchers of Ukraine by providing them with an overview of all existing actions at European and national levels	The EU	https://euraxess.ec.europa.eu/ukraine
European Federation of Academies of Sciences and Humanities (ALLEA)	European fund for displaced scientists	The EU	https://allea.org/european-fund-for-displaced-scientists
ERC4Ukraine	European Research Council (ERC) initiative provide temporary employment to refugee researchers and support staff	The EU	https://erc.europa.eu/apply-grant/erc-ukraine
The Guild	The Guild of European Research-Intensive Universities Association support initiative	The EU	https://www.the-guild.eu/resources/the-guild-s-universities-supporting-researchers-ac.html
IIE Scholar Rescue Fund	A global program that arranges funds and supports fellowships for threatened and displaced scholars	The EU	https://www.scholarrescuefund.org
Labs Supporting Ukrainian Scientists	An online database to help find positions for displaced scholars from Ukraine at the University of Oregon	USA	https://tinyurl.com/yanb37ck
Le Collège de France	A fund featuring state-sponsored opportunities for displaced scientists	France	https://www.college-de-france.fr/
OEG Connect	An open education resource to connect, share, and work together to make learning accessible	~~International~~ Inter-national	https://connect.oeglobal.org/
Philipp Schwartz Initiative of the Humboldt Foundation	A program to help researchers subject to significant personal threat in their country to continue their work at German universities	Germany	https://www.humboldt-foundation.de
MSCA4Ukraine	A subsidiary of the EU's Marie Skłodowska-Curie Actions, to provide fellowship support to researchers from Ukraine	The EU	https://sareurope.eu/msca4ukraine/

Saving Ukrainian Cultural Heritage Online (SUCHO)	A group of volunteers to web-archive digitize and preserve heritage from Ukrainian cultural institutions (no research support)	Inter-national	https://www.sucho.org
Scholars at Risk (SAR)	International network to protect threatened scholars and promote academic freedom around the world. Cooperating with several national science foundations in Europe	Inter-national	https://www.scholarsatrisk.org
Scientists and Engineers in Exile or Displaced (SEED) program	A collaboration of National Academies to award grants	USA and Poland	https://www.nationalacademies.org/our-work/scientists-and-engineers-in-exile-or-displaced-seed-program
Shevchenko Emergency Fund	A fund to support scholars by Shevchenko Scientific Society the largest and the oldest Ukrainian scientific organization outside of Ukraine	USA	https://shevchenko.org
Swiss National Science Foundation	A state-sponsored opportunities for displaced scientists	~~Switzerland~~Switzerland	https://www.snf.ch/en
The Association for Slavic, East European & Eurasian Studies	Resource database to help scholars from Ukraine	USA	https://www.aseees.org/resources/help-displaced-scholars-ukraine
The UK-Ukraine Twinning Initiative	An institution-to-institution collaboration model to lay the foundations of strong partnerships between the UK and Ukrainian universities supported by grants from UKRI's Research England	UK and Ukraine	https://www.twinningukraine.com/
Ukrainian Global University Initiative	A consortium of leading Ukrainian educational institutions and organizations to support Ukrainian students scholars with the opportunities for quality education and research	Ukraine	https://uglobal.university
Ukrainian Scholar Placement Database	An online database to help find positions for displaced scholars from Ukraine at Sam Houston State University	USA	https://tinyurl.com/2f8csafa

Existing cooperation networks that were in place before the war were used to rapidly launch or amplify the support measures. A group of initiatives was rising from existing pre-war structures, notably national societies, and academies. For example, the Office of Policy and Global Affairs, a Division of the US National Academies previously ~~engage~~engaged in science diplomacy among different nations, in collaboration with the Polish Academy of Sciences quickly created the *Scientists and Engineers in Exile or Displaced initiative* to support several hundreds of Ukrainian scholars with short~~-~~-term stipends for studies outside of Ukraine (PAS-NAS SEED, **Table 1**). The publicly-funded *Shevchenko Scientific Society* (the largest and the oldest Ukrainian scientific organization outside of Ukraine founded in 1873) started to create opportunities for the displaced scientists supported by its US and Canada~~-~~-based members. Most of the national academies in the EU block issued supportive statements to Ukraine, and within weeks, the *European Federation of Academies of Sciences and Humanities* (ALLEA, **Table 1**) using a donation from the Breakthrough Prize Foundation started hosting scholars displaced by the war. After millions of

refugees –crossed the EU border and settlingsettled in the various European countries, more opportunities were created by national organizations such as the *Swiss National Science Foundation*, *the Polish Academy of Sciences, and Le Collège de France*. The European Commission opened the *European Research Area portal* for Ukraine, and *Horizon Europe*, with its complementary *Euratom Research and Training Programme*, offered free participation to support the Ukrainian scientific community. The *MSCA4Ukraine* consortium funded under the EU's Marie Skłodowska-Curie Actions, provideprovides fellowship support for hundreds of displaced researchers from Ukraine.

However, national academies were only one source of support. More active were global research networks, and discipline-specific international associations. International organizations, such as *Scholars at Risk* (SAR, **Table 1**), formed as far back as in 1999 to protect threatened scholars and promote academic freedom around the world, and others (*IIE Scholar Rescue Fund, Philipp Schwartz Initiative of the Humboldt Foundation in Germany, Council for At-Risk Academics in the UK*, and others (**Table 1**)) backed by governments, philanthropists and large corporate sponsors rapidly focused their efforts and started creating opportunities for the displaced Ukrainians as well. The UK--based twinning initiative, an institution-to-institution collaboration model, incorporated the UK-Ukraine Research and Innovation twinning grants from UKRI's Research England to enable twins to further their research and innovation collaborations. In a relatively short time, individual universities, research institutes, and other academic organizations from all across the globe started offering scholarships, paid positions, and other types of support on their own. Volunteer initiatives, and the unwavering support received from an array of universities, academic institutions, scientific societies, publishers, and funding agencies worldwide were met with great gratitude from the Ukrainian scientific community [18]. This unprecedented widespread support will be crucial on the path to recoverrecovery and mitigate the consequences of the war for academic and scientific communities and their institutions in the country. The networks established early at the start of the war laid a foundation for the further integration of Ukrainian scientists into the global community, and will become grounds offor the future success of more formal organizational initiatives involving Ukraine that are being developed at this time (i.e. *Ukrainian Global University Initiative, Horizon Europe*, etc.).

Despite these opportunities, so far only a fraction of scholars could physically relocate abroad and take advantage of the opportunities offered to them by the global response, the rest remained in Ukraine. This is a complex issue. According to the rules of martial law in Ukraine, males between the ages of 18 and 60 cannot leave the country. Many academics joined the Territorial Defense or were drafted into the Ukrainian Armed Forces [19]. Some did not want to leave their families and homes in Ukraine, for a short research opportunity abroad, or needed to take care of their children and the elderly who could not come along. Even among those who could leave, many were discouraged by the bureaucratic process of obtaining long-term visas.

Those staying in Ukraine found themselves in the most unstable and vulnerable situation. Many universities, especially those in the east of the country are either closed or relocated to safer areas. The budgets of the state--run universities were cut to save funds for the war effort. While the base salaries for teaching are usually maintained, these were traditionally already very low and commonly supplemented by income from funded research and overtime work, which are now dropped [20]. The national research programs have been plagued by the issues associated with the

Confidential: do not distribute.

war, and the funding has mostly been cut off or frozen. Despite the dire situation, many researchers in Ukraine continued to maintain their academic and research activities.

The Ukrainian crisis has several distinct challenges, and it invoked a complex response with many underlying factors, including historical, cultural, economic, and political factors. The initial reaction of the scientific community was to provide opportunities for refugees fleeing the country. Some of the recent examples resulted in ~~a number of~~many scholars seeking refuge. Under the current political regime, about 2,000 highly skilled Venezuelan scientists responsible for the publication of a third of all scientific papers from Venezuela and ~~represent~~representing 15 percent of the Venezuelan research community, have left their academic posts and fled abroad [21]. The recent crisis associated with the withdrawal of US troops ~~in~~from Afghanistan forced many researchers fleeing and those who ~~remain face~~remained lost funding and ~~the~~faced a threat of persecution [22]. The Syrian civil war has been ongoing since 2011 and has caused significant humanitarian, economic, and political challenges producing science refugees: academics and scientists that required help outside their country [23]. Initially, it seemed that the war in Ukraine would produce a similar result, and the research community immediately began preparing for the influx of researchers who needed a place to live and work outside of their country.

Analysis of the opportunities for Ukrainian scientists listed in the *#ScienceForUkraine* database

To understand what types of opportunities have been offered to Ukrainian scientists during the first year of the war, we surveyed and classified the entries posted on the *#ScienceForUkraine* website one year ~~since~~after the start of the invasion (February 2022 - February 2023). The goal of this initiative was to help provide support for the Ukrainian academic community and also streamline any setbacks for the Ukrainian scientific community[16]. While this database may be incomplete, and some entries have been deleted (personal communication), it is still the largest publicly available database that can be surveyed for the types of opportunities that have been offered.

A review of the *#ScienceForUkraine* database shows a clear trend towards in-person and ~~the~~a lack of ~~the~~ remote opportunities (**Table 2, Figure 1**). *#ScienceForUkraine* volunteers tried to achieve this by raising awareness among research communities across the world about the situation, posting about new opportunities for students and scholars to receive opportunities across the world, and working with various European funding organizations to help support the ~~-~~academic community. We used the default classification of this database into "*paid positions*", "*educational*", "*funding programs*" and "*partnerships*" **(Figure 1A)**. The vast majority of the 1,871 listed opportunities listed were "*paid positions*". Opportunities labeled as "*educational*" and "*funding programs*" were distributed in ~~a~~ similar proportions (**Table 2, Figure 1A**).

Overall, all classes of opportunities were distributed roughly equally between "*researchers*" and "*students*" of every level, with very few indicated as "*professional*" (**Figure 1.B**). It is difficult to classify these into distinct sub-categories, but generally, they included research and postdoctoral fellowships, as well as some researcher jobs. Unsurprisingly, most of them were located in Europe (n=1,293), with North America (the US and Canada) as a distant second (n=181). Germany had the most paid positions, followed by France, Poland, Canada, and the United States (**Figure 1.C**). Germany also had the most opportunities in education as well as funding programs (**Figure 1. D,E**).

Confidential: do not distribute.

Generally, since most of these initiatives were trying to deal with the refugee crisis at hand, their success relied largely on the readiness of the Ukrainian scientists to move abroad. The review of the #ScienceForUkraine database demonstrates that most entries require travel, and shows the relative scarcity of remote opportunities for scholars that cannot leave the country during the time of war: only 52 positions (out of 1,871 available), specifically mentioned the word “remote” and most of them suggest a possibility of relocation. Despite the vital importance of initiatives offering temporary or permanent positions, they target opportunities for only a fraction of the scientists who left or were able to leave the country. As a result, very often the existing opportunities may go unfulfilled which would frustrate those who offer them, as well as those who could not use them.

Table 2. Locations of opportunities for Ukrainian scientists in the *#ScienceForUkraine* database.

Type	All Available	Europe	North America	Asia	South America	Oceania	Africa
**Paid Positions**	1,456 (83%)	1,293	181	44	11	12	2
**Education Programs**	170 (9%)	145	12	8	0	2	0
**Funding Programs**	67 (4%)	53	8	1	1	0	0
**Partnerships**	6 (<1%)	1	2	0	1	1	0
**Other Help**	77 (4%)	68	5	0	0	0	0
**Unspecified**	5 (<5%)	3	0	1	1	0	0
**Total**		1,563	208	54	14	15	2

Figure 1. Locations of opportunities for Ukrainian scientists in the *#ScienceForUkraine* database in one year since the start of the conflict (February 2022-February 2023).

A. Number of opportunities by program type. The majority of the 1,871 listed opportunities can be classified as paid positions. These include research and postdoctoral fellowships, as well as some researcher jobs. Most of these are in Europe (1,293), with North America as a distant second (181). Educational opportunities and funding programs were distributed in a similar proportions.
B. Number of opportunities by applicant qualification. Opportunities were distributed roughly equally between researchers and students of every level, with very few indicated as “*professional*.”.
C, D, and E. Distribution of different types of opportunities (listed in **Fig. 1.A**) by country. The total number of opportunities per category is given in parentheses. Germany had the most opportunities in all categories.

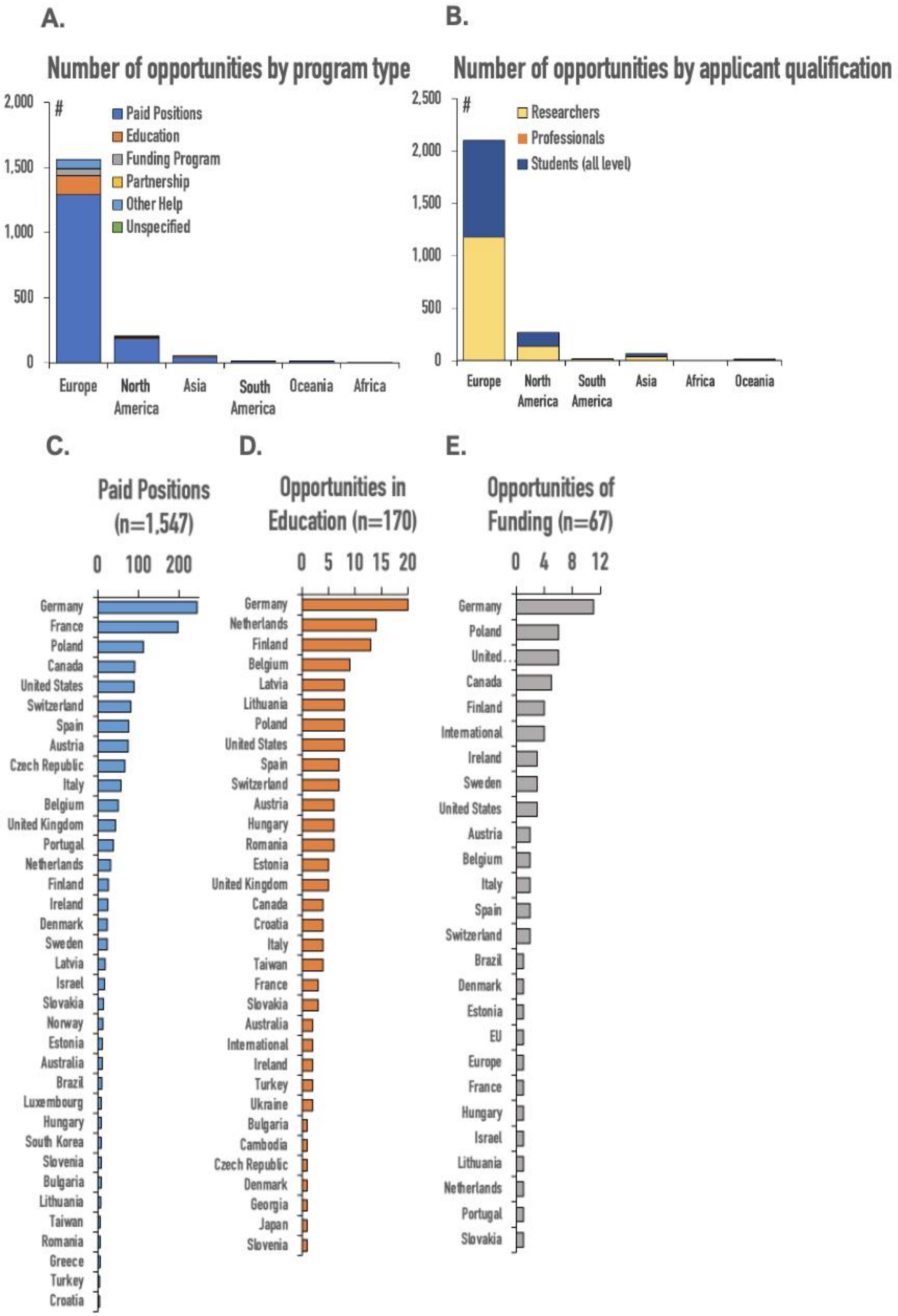
A.
Number of opportunities by program type
#
Paid Positions
Education
Funding Program
Partnership
Other Help
Unspecified
2,000
1,500
1,000
500
0
Europe
North America
Asia
South America
Oceania
Africa
B.
Number of opportunities by applicant qualification
#
Researchers
Professionals
Students (all level)
2,500
2,000
1,500
1,000
500
0
Europe
North America
South America
Asia
Africa
Oceania
C.
Paid Positions (n=1,547)
0 100 200
Germany
France
Poland
Canada
United States
Switzerland
Spain
Austria
Czech Republic
Italy
Belgium
United Kingdom
Portugal
Netherlands
Finland
Ireland
Denmark
Sweden
Latvia
Israel
Slovakia
Norway
Estonia
Australia
Brazil
Luxembourg
Hungary
South Korea
Slovenia
Bulgaria
Lithuania
Taiwan
Romania
Greece
Turkey
Croatia
D.
Opportunities in Education (n=170)
0 5 10 15 20
Germany
Netherlands
Finland
Belgium
Latvia
Lithuania
Poland
United States
Spain
Switzerland
Austria
Hungary
Romania
Estonia
United Kingdom
Canada
Croatia
Italy
Taiwan
France
Slovakia
Australia
International
Ireland
Turkey
Ukraine
Bulgaria
Cambodia
Czech Republic
Denmark
Georgia
Japan
Slovenia
E.
Opportunities of Funding (n=67)
0 4 8 12
Germany
Poland
United...
Canada
Finland
International
Ireland
Sweden
United States
Austria
Belgium
Italy
Spain
Switzerland
Brazil
Denmark
Estonia
EU
Europe
France
Hungary
Israel
Lithuania
Netherlands
Portugal
Slovakia

Confidential: do not distribute.

Effective and feasible mechanisms to support scientists and students from Ukraine

We hope to use the ongoing struggle in Ukraine to initiate the conversation about effective and feasible mechanisms for the support of scholars and students at risk. There is a need ~~of~~for discussion on the feasibility and efficacy of various approaches to short-term and long-term strategies that help scholars during the active phase of conflicts and ~~during~~ post-war reconstruction periods. Specifically, we want to bring the attention of the international community to creating scalable mechanisms that can be offered to several thousands of ~~the~~ scientists who have left their respective ~~country~~countries, versus ~~for~~ those who are ~~still~~ remaining in the country. It would only make sense to complement the opportunities that require travel with the ones that can be more suitable for the scientists staying in Ukraine: offering a wider range of remote positions, engaging the scientific community in the global community, and offering effective training to address any existing knowledge gaps [1]. This combined strategy would help Ukrainian science to endure ~~during the~~ difficult times, secure their development, and help further integrate them into the international scientific community. This will also set an example for other scientific communities at risk. We use Ukraine as an example from our ~~own~~ collaborations, but the recommendations herein can be used to support research initiatives of scientists in other countries.

The effective remote opportunities described here could be sorted into two groups (**Table 2**). In the first group, there are efforts that are directed towards and imply establishing ~~a~~ contact with a specific scientist or a group of scientists in Ukraine (**Table 2, A-D**). In the second group, there are venues to help Ukrainian researchers indirectly, without knowing or contacting specific researchers or scientific groups (**Table 2, E-H**).

Table 2. Mechanisms to support scientists and students from countries experiencing hardship. Group 1 (A-D) includes efforts that are directed towards and imply establishing ~~a~~ contact with a specific scientist or a group of scientists in Ukraine. Group 2 (E-H) In the second group, there are venues to help Ukrainian researchers indirectly, without knowing or contacting specific researchers or scientific groups.

	#	Mechanism	Description
Group 1	A	**Establish direct remote research collaborations**	A scientist or researcher in can collaborate remotely with Ukrainian scientists directly on common research projects.
	B	**Donate funds and provide access to resources**	Funds can be donated directly to research institutions to support their work
	C	**Share research opportunities**	Informing colleagues about opportunities that could be missed because of the limited access or language barriers.
	D	**Participate in online events inside the country**	Taking part in webinars, online conferences, and workshops put together by local organizers
Group 2	E	**Promote publications in the scientific journals**	Encouraging journals to support researchers by waving publication fees, and providing translations and editorial services, and including versions of published papers in authors native languages.
	F	**Promote participation in international conferences**	Encourage organizers of scientific conferences to provide free or discounted access to registration and memberships.
	G	**Enabling opportunities for remote education and training**	Provide and encourage other groups to provide online training to provide have adequate competitive for the multitude of the positions offered by the global scientific community.
	H	**Encourage funding agencies to provide funding opportunities for** the research **communities facing hardship**	Encourage funding agencies, non-profits, foundations governmental, and academic institutions to establish new research funding opportunities for international

	collaborations, prioritizing joint research and academic projects and programs.

Remote research collaborations might involve sharing data, co-authoring papers, or providing expertise in a particular field (**Table 2, Group 1, A-D**). These strategies can help support scientific research in their country and provide opportunities for researchers to work together and share expertise. Due to the rapid advancements in computer technology, researchers from various fields can be involved in multi-institutional research and contribute remotely which has proven to be effective [24]. The most straightforward way to do it is for the individual scientists to look up and invite colleagues from their field who work in the country under attack to engage in research or initiate new scientist projects which can be submitted to various international journals (**Table 2, Group 1, A**). This would have an immediate and personal impact, and the connectivity of science in Ukraine will increase from submitting joint peer-reviewed publications in English to the press and ~~provide~~providing a much-~~-~~needed endorsement and strengthening of local science.

If there are no common interests in collaboration, there are still other venues to help support science. For example, if funds are available, they can be donated directly to research institutions to support their work inside the country (**Table 2, Group 1, B**). This might include science organizations, public and private universities, research institutes, and non-governmental organizations that support science. If there are no monetary funds, sharing resources can also be an effective help. For example, sharing access to scientific journals, databases, online tools, or software can prove essential for research activities in ~~the~~ regions of crisis. Help can also be provided by sharing news and opportunities related to their research on social media or other online platforms such as *#ScienceForUkraine* and others (**Table 2, Group 1, C**) that can help find potential collaborators, mentors, and students, raise awareness of their work and connect them with potential funding opportunities.

Finally, the global scientific community is encouraged to be more proactive in registering and participating in events that are held inside Ukraine (**Table 2, Group 1, D**). Some scientific organizations and universities in the country have been putting together webinars, online conferences, and workshops that encouraged remote participation by ~~the~~ international colleagues. For example, soon after the invasion, the Institute of Molecular Biology and Genetics, at the NAS of Ukraine organized an "*All-Ukrainian Conference on Molecular and Cell Biology with international participation*" (June 2022).

Online events like this provide a great opportunity to learn about current research and connect with potential collaborators in the country and provide a much-~~-~~needed endorsement and strengthening of local science. Online conferences have ~~a number of~~many advantages, such as being more accessible to a wider range of participants and reducing travel costs. At the same, there were several issues, including the loss of informal networking opportunities, the difficulty ~~of~~in maintaining attention during online presentations, and the potential for technical problems [25]. These can be avoided in hybrid-~~-~~style conferences~~,~~ but must be addressed~~ in order~~ to maximize the positive impact on scientists at risk that can only attend virtually.

Researchers at risk can also be helped indirectly by modifying the policies of scientific publishers, research foundations, and academic ~~institution~~institutions (**Table 2, Group 2, E-H**). For example, scientific journals could support Ukrainian researchers by ~~waving~~waiving publication fees~~,~~ and

Confidential: do not distribute.

helping with translation and editing. Inviting Ukrainian colleagues onto editorial boards ~~an~~has the effect of increasing the engagement of scientists in Ukraine (**Table 2, Group 2, E**). Some journals ~~has~~have offered a special issue based on the works ~~from~~of Ukrainian members [26]. Journals could allow multilingual support by adding translated versions of their publications in the native languages of the authors [27]. PubMed Central already has a policy enabling accept non-English articles and/or English articles with non-English parts based on the agreements between the publisher and NLM [28].

Organizations and societies that hold scientific conferences can waive fees for scientists participating virtually to allow researchers that cannot leave their country to participate. Many scientific conferences are already hosted in a mixed~~-~~ mode allowing in-person as well as virtual attendance. This helps break administrative barriers and this is well suited for scientists not able to leave their country [29]. ~~–~~Scientific societies can help by waiving the registration fee for academics and researchers so they can participate in ~~the~~ international scientific life without leaving the country (**Table 2, Group 2, F**). While writing this article we approached organizers of several international conferences to ease access for the Ukrainian scientists this year. As a result, *The European Society ~~crisis~~of Human Genetics* (ESHG) has decided to waive the 2023 membership fee for all Ukrainian geneticists wishing to join ESHG that have a professional life focus in Ukraine or have been forced to leave Ukraine due to the war. *The Society of Molecular Biology and Evolution* (SMBE) developed a hybrid arrangement to encourage scientists who cannot attend the conference in person to apply~~,~~ and will consider waiving registration fees (in person or online) to scientists because of their inability to pay it. *The International Society of Computational Biology* (ISCB) is ~~offered~~offering a reduced registration fee to the lowest level for ~~the~~ online participation in ISMB/ECCB 2023 Conference, to Ukrainian scientists that remain in Ukraine or have been forced to leave Ukraine due to the war.

Training (**Group 2, G**) is another valuable tool to ensure scientific communities in the affected countries ~~to be~~are more engaged. [30]There are groups in Ukraine that are eager to participate and be incorporated in ~~the~~ remote scientific research collaborations with minimal training. For instance, because of its well-developed education system, Ukraine has a significant number of Computer Science undergraduates. These students and recent graduates are well suited for remote work and computational research but normally, after graduating college, they would skip graduate school to pursue the well-paid outsourced jobs in the various industries attracted by significant financial incentives offered by the recruiting companies. In the new reality created by the ongoing invasion, these skilled informaticians are facing limited options in the industry. However, since graduate school provides a waiver of military mobilization, they started flocking to graduate careers. This generated a pool of potential researchers who cannot leave the country, have the required skills~~,~~ and are willing to collaborate on scientific projects remotely. Training this particular group could for instance enable the creation of a bioinformatics research community that can be later engaged in international collaborations[30]. However, training requires funding, and we urge funding agencies to enable funding initiatives to train scientists in bioinformatics skills which will allow them to remotely engage in large international consortiums and establish collaboration with the global community. Lack of language skills can erect additional barriers, as not all scientists are proficient in English.

Funding needs to be allocated to encourage and educate scholars who require these abilities to participate in international collaborative opportunities (**Table 2, Group 2, H**). The most flexible

Confidential: do not distribute.

and scalable mechanism is to offer remote training and paid remote opportunities for the scientists as well as find appropriate funding to support such opportunities. Online databases addressed some of these issues, but ~~there are~~ many remaining issues are hindering their effectiveness. Particularly, because many of these ~~position~~positions are general advertisements for positions available, and not ~~really~~specifically geared toward Ukrainian scholars at risk, candidates must compete with other applicants that are not disadvantaged by the war. Even if students and researchers are interested in these opportunities, they need to navigate the specifics of education systems in different respective countries. Due to the combination of political, economic, historical, and cultural reasons, many of the Ukrainian researchers that could benefit from these opportunities, cannot compete for them, as they ~~the~~ lack the skills necessary to find jobs in science outside their country, and often simply do not satisfy the requirements of available job openings. Unless the jobs are specifically created, they would need additional training just to be competitive. To address these issues, we suggest that a platform needs to be developed that could ~~matches~~match scientists and computer experts with laboratories and faculties willing to offer remote mentorships or employment. [31]

During the COVID-19 pandemic, we all learned that remote learning is possible and effective if the educators are creative and the students are willing. Educators that have developed online resources, could team up with educators in crisis zones to co-teach courses assisted by technology. The session could meet synchronously or asynchronously (recording sessions to account for power outages or internet service lags), and both teachers could teach different portions of the course. Students could even collaborate ~~in~~on projects, work could be divided based on the availability of resources, and while some students could do lab work, ~~while other~~others might focus on data analysis. Together they could write reports or scientific articles, fostering the next generation of international collaborations. Training also requires teaching materials, and some initiatives like OER (Open Education Resources) can provide them. Historically, OER has been more aimed at younger students but their community has been working to expand the current limitations (**Table 1,** *OEG Connect)*.

The global scientific community and decision-making authorities urgently need to implement a coordinated assistance plan targeted to scientists at risk that are remaining in the invaded country. Some of the authors of this publication created an interdisciplinary group ~~consists~~consisting of researchers from countries, including Ukraine, the United States, the UK, Germany, and others who share concerns about the fragile situation of the scientific community in these wartime countries and argue about the need to develop effective and feasible mechanisms to support researchers and students at risk along with voices around the world. ~~It is vital for~~ Governments and the private sector need to establish new research funding opportunities for international collaborations, including joint research and academic projects and programs [31]. An early example of such efforts include Action Steps for Rebuilding Ukraine's Science, Research, and Innovation by the Academy of US Poland and Ukraine [32] and they mentioned the need for remote opportunities but did not provide details on how to enable such opportunities. Another ~~examples~~example of a decision-making authority is the United States Agency for International Development (USAID) or The United Nations Educational, Scientific and Cultural Organization (UNESCO) which provide civilian aid and promote social and economic development and international cooperation. But despite these organizations being present worldwide, and even though many individual labs and faculty across the globe are willing to

mentor and involve scientists in existing projects, they lack the financial means and mechanisms to support and engage them.

~~[32]~~Effective Mechanisms of remote support scientists at risk

As science in the world is becoming more interconnected, scientists in countries at risk from war, political strife, natural disasters, ecological crisis, or economic hardship ~~and~~ can continue functioning as part of the international community and can be incorporated ~~in~~into the research process remotely. Thanks to the new capabilities in collaborations, they can continue working together across borders and disciplines and help global efforts to tackle global challenges and advance our understanding of the world.

The lessons learned from the current crisis in Ukraine precipitated by the unprovoked Russian invasion can be readily applied to other similar situations. Using Ukraine as an example, we have established ~~a number of~~several possible venues of assistance to scientific communities facing hardship. This, of course, is not limited to Ukraine~~,~~ but can be applied to other situations. Unfortunately, there are too many examples of similar situations, some resulting from the war ~~as~~ in Ukraine, others from persecution ~~as~~ in Venezuela, Afghanistan, and Syria, and natural disasters starting from Hurricanes Maria and Fiona in Puerto Rico to the recent earthquakes in Turkey and Syria. In this paper, we outlined ~~a number of~~several effective and feasible possible venues to support scientists and students in countries facing hardship, or disadvantaged by the military, political ~~economical~~economic, or ecological crisis (**Table 2**). The list of opportunities is by no means exhaustive and is meant to begin, not ~~to~~ conclude the conversation on this issue.

Creating ~~the~~ online resources like *#SceinceForUkraine* was an immediate and successful example of what can be applicable and immediately adapted to political strife and war zones, and to areas where natural disasters have occurred. Many opportunities listed on *#ScienceForUkraine* and similar boards~~,~~ are not specifically created to recruit Ukrainians, these platforms are used to incorporate Ukrainian ~~audience~~audiences to recruit for existing positions. With that caveat, its overall effect was positive, because researchers that are looking for opportunities are exposed to a wide variety of options. However, the best scenario is to establish new research funding opportunities for international collaborations, including joint research and academic projects and programs.

There is an urgent need to launch a discussion about the feasible long-term plan to rebuild science and create research opportunities. Programs and mechanisms should be created to allow scientists that cannot leave their home countries can still acquire novel skills remotely, and these skills can then be disseminated to the local scientific community of their designated country. For the researchers that remain in the country, transitioning to computational data-driven research can be a good solution, as many skills can be taught remotely and accessed from anywhere in the world. Systematic training that is needed in ~~the~~ state-of-the-art analytical skills can be achieved by developing plans which can include establishing collaboration with world-leading institutions. ~~The~~ Remote work is used not only for data-driven research familiar to the reader of this journal~~,~~ but also for the most of humanities and social sciences as well. Remote mechanisms should be created allowing world~~-~~-class specialists to teach and train undergraduate and graduate students [32]. The engagement of university leadership to make remote arrangements possible is important, however, this is still not widely adopted, and funding agencies should provide specific funding

initiatives to support these areas. Integrating new domains of research, which are not relying on physical infrastructure but instead are based on computational research, can result in a shorter recovery time for rebuilding Ukrainian science compared to the restoration of physical infrastructure.

Acknowledgments

Our paper is dedicated to all freedom-loving people around the world, and to the people of Ukraine who fight for our freedom. Special thanks to Anita Bandrowski, Oleksandra V. Ivashchenko and Sanita Reinsone for the helpful review, valuable criticism, and useful suggestions while preparing this manuscript.

References

[1] K. Chhugani, A. Frolova, Y. Salyha, A. Fiscutean, O. Zlenko, S. Reinsone, W.W. Wolfsberger, O. v. Ivashchenko, M. Maci, D. Dziuba, A. Parkhomenko, E. Bortz, F. Kondrashov, P.P. Łabaj, V. Romero, J. Hlávka, T.K. Oleksyk, S. Mangul, Remote opportunities for scholars in Ukraine, Science (1979). 378 (2022) 1285–1286. https://doi.org/10.1126/science.adg0797.

[2] THE GLOBAL ALLIANCE FOR GENOMICS AND HEALTH, A federated ecosystem for sharing genomic, clinical data, Science (1979). 352 (2016) 1278–1280. https://doi.org/10.1126/science.aaf6162.

[3] R. Yuste, C. Bargmann, Toward a Global BRAIN Initiative, Cell. 168 (2017) 956–959. https://doi.org/10.1016/j.cell.2017.02.023.

[4] R.A. Gibbs, The Human Genome Project changed everything, Nat Rev Genet. 21 (2020) 575–576. https://doi.org/10.1038/s41576-020-0275-3.

[5] L. Horyn, The Large Hadron Collider, in: A Search for Displaced Leptons in the ATLAS Detector, Springer Nature Switzerland AG, 2022: pp. 21–29. https://doi.org/10.1007/978-3-030-91672-5_3.

[6] F. Miedema, Open Science: the Very Idea, Springer Netherlands, Dordrecht, 2022. https://doi.org/10.1007/978-94-024-2115-6.

[7] UNESCO, UNESCO Recommendation on Open Science, UNESCO Headquarters (Paris) - UNESCO Archives (Paris), Paris, 2021.

[8] D. Cirillo, A. Valencia, Big data analytics for personalized medicine, Curr Opin Biotechnol. 58 (2019) 161–167. https://doi.org/10.1016/j.copbio.2019.03.004.

[9] J. Li, H. Chen, Y. Wang, M.-J.M. Chen, H. Liang, Next-Generation Analytics for Omics Data, Cancer Cell. 39 (2021) 3–6. https://doi.org/10.1016/j.ccell.2020.09.002.

[10] P.S. Hosmani, T. Shippy, S. Miller, J.B. Benoit, M. Munoz-Torres, M. Flores-Gonzalez, L.A. Mueller, H. Wiersma-Koch, T. D'Elia, S.J. Brown, S. Saha, A quick guide for student-driven community genome annotation, PLoS Comput Biol. 15 (2019) e1006682. https://doi.org/10.1371/journal.pcbi.1006682.

[11] E.F. Ryder, W.R. Morgan, M. Sierk, S.S. Donovan, S.D. Robertson, H.C. Orndorf, A.G. Rosenwald, E.W. Triplett, E. Dinsdale, M.A. Pauley, W.E. Tapprich, Incubators: Building community networks and developing open educational resources to integrate bioinformatics into life science education, Biochemistry, and Molecular Biology Education. 48 (2020) 381–390. https://doi.org/10.1002/bmb.21387.

[12] G. Cantelli, G. Cochrane, C. Brooksbank, E. McDonagh, P. Flicek, J. McEntyre, E. Birney, R. Apweiler, The European Bioinformatics Institute: empowering cooperation in response to a global health crisis, Nucleic Acids Res. 49 (2021) D29–D37. https://doi.org/10.1093/nar/gkaa1077.

Confidential: do not distribute.

[13] T.K. Oleksyk, W.W. Wolfsberger, K. Shchubelka, S. Mangul, S.J. O'Brien, The Pioneer Advantage: Filling the blank spots on the map of genome diversity in Europe, Gigascience. 11 (2022). https://doi.org/10.1093/gigascience/giac081.
[14] N. v. Fedoroff, Science Diplomacy in the 21st Century, Cell. 136 (2009) 9–11. https://doi.org/10.1016/j.cell.2008.12.030.
[15] M. Maryl, O. v. Ivashchenko, M. Reinfelds, S. Reinsone, M.E. Rose, Addressing the needs of Ukrainian scholars at risk, Nat Hum Behav. 6 (2022) 746–747. https://doi.org/10.1038/s41562-022-01387-7.
[16] M. Rose, S. Reinsone, M. Andriushchenko, M. Bartosiak, A. Bobak, L. Drury, M. Düring, I. Figueira, E. Gailīte, I. Gozhyk, L.G. Abreu, I. Gutierrez, O. Ivashchenko, K. van Heuckelom, J. Jaudzema, K. Jurikova, A. Klos, J. Knörzer, E. Kutafina, M. Kwaśnicki, H. Lane, I. Ļaksa-Timinska, B. Laschowski, A. Lattu, M. Maci, K. Mäkinen-Rostedt, M. Maryl, M. van Meerbeek, O. Morin, V. Mosienko, A. Palou Vilar, K. de Pauw, M. Pelepets, M. Reinfelds, C. Rujan, Z. Santybayeva, A. Skatova, M. Vita, I. Weaver, M. Wnuk, R. Beckett, #ScienceForUkraine: an Initiative to Support the Ukrainian Academic Community. "3 Months Since Russia's Invasion in Ukraine", February 26 – May 31, 2022, SSRN Electronic Journal. (2022). https://doi.org/10.2139/ssrn.4139263.
[17] A. Heyman, OU's Ukrainian community ask administration for financial assistance, Oakland Post. (2022). https://oaklandpostonline.com/41529/campus/ous-ukrainian-community-ask-administration-for-financial-assistance/ (accessed March 1, 2023).
[18] A. Klimash, Ukraine's Chemists Persevere Through A Year Of War, Chemistry World. (2023). https://www.chemistryworld.com/opinion/ukraines-chemists-persevere-through-a-year-of-war/4017035.article (accessed April 11, 2023).
[19] N. Gaind, How three Ukrainian scientists are surviving Russia's brutal war, Nature. 605 (2022) 414–416. https://doi.org/10.1038/d41586-022-01272-3.
[20] M. Maryl, M. Jaroszewicz, I. Degtyarova, Y. Polishchuk, M. Pachocka, M. Wnuk, Beyond Resilience: Professional Challenges, Preferences, and Plans of Ukrainian Researchers Abroad, Zenodo. (2022) 4–30. https://doi.org/10.5281/zenodo.7380509 (accessed April 11, 2023).
[21] N. Casey, Hungry Venezuelans Flee in Boats to Escape Economic Collapse, New York Times. (2016). https://www.nytimes.com/2016/11/25/world/americas/hungry-venezuelans-flee-in-boats-to-escape-economic-collapse.html?smid=url-share (accessed March 1, 2023).
[22] S. Mallapaty, Afghanistan's terrified scientists predict huge research losses, Nature. 597 (2021) 15–16. https://doi.org/10.1038/d41586-021-02353-5.
[23] T.M. Stoepler, J.E. Creswell, N.J. Anthis, F. Chaverneff, R.R. Parkhurst, The Role of Science Diplomacy in International Crises: Syria as a Case Study, Sci Dipl. 6 (2017). https://www.sciencediplomacy.org/article/2017/role-science-diplomacy-in-international-crises-syria-case-study (accessed March 1, 2023).
[24] P. Hunter, Remote working in research, EMBO Rep. 20 (2019). https://doi.org/10.15252/embr.201847435.
[25] E. Levitis, C.D.G. van Praag, R. Gau, S. Heunis, E. DuPre, G. Kiar, K.L. Bottenhorn, T. Glatard, A. Nikolaidis, K.J. Whitaker, M. Mancini, G. Niso, S. Afyouni, E. Alonso-Ortiz, S. Appelhoff, A. Arnatkeviciute, S.M. Atay, T. Auer, G. Baracchini, J.M.M. Bayer, M.J.S. Beauvais, J.D. Bijsterbosch, I.P. Bilgin, S. Bollmann, S. Bollmann, R. Botvinik-Nezer, M.G. Bright, V.D. Calhoun, X. Chen, S. Chopra, H. Chuan-Peng, T.G. Close, S.L.

Cookson, R.C. Craddock, A. De La Vega, B. De Leener, D. V Demeter, P. Di Maio, E.W. Dickie, S.B. Eickhoff, O. Esteban, K. Finc, M. Frigo, S. Ganesan, M. Ganz, K.G. Garner, E.A. Garza-Villarreal, G. Gonzalez-Escamilla, R. Goswami, J.D. Griffiths, T. Grootswagers, S. Guay, O. Guest, D.A. Handwerker, P. Herholz, K. Heuer, D.C. Huijser, V. Iacovella, M.J.E. Joseph, A. Karakuzu, D.B. Keator, X. Kobeleva, M. Kumar, A.R. Laird, L.J. Larson-Prior, A. Lautarescu, A. Lazari, J.H. Legarreta, X.-Y. Li, J. Lv, S. Mansour L., D. Meunier, D. Moraczewski, T. Nandi, S.A. Nastase, M. Nau, S. Noble, M. Norgaard, J. Obungoloch, R. Oostenveld, E.R. Orchard, A.L. Pinho, R.A. Poldrack, A. Qiu, P.R. Raamana, A. Rokem, S. Rutherford, M. Sharan, T.B. Shaw, W.T. Syeda, M.M. Testerman, R. Toro, S.L. Valk, S. Van Den Bossche, G. Varoquaux, F. Váša, M. Veldsman, J. Vohryzek, A.S. Wagner, R.J. Walsh, T. White, F.-T. Wong, X. Xie, C.-G. Yan, Y.-F. Yang, Y. Yee, G.E. Zanitti, A.E. Van Gulick, E. Duff, C. Maumet, Centering inclusivity in the design of online conferences—An OHBM–Open Science perspective, Gigascience. 10 (2021). https://doi.org/10.1093/gigascience/giab051.

[26] A.O. Cherninskyi, Global Excellence in Cellular Neuropathology: Ukraine, Frontiers in Cellular Neuroscience, 2023. https://www.frontiersin.org/research-topics/46571/global-excellence-in-cellular-neuropathology-ukraine (accessed March 1, 2023).

[27] A.J. Majeske, A.J. Mercado Capote, A. Komissarov, A. Bogdanova, N. v. Schizas, S.O. Castro Márquez, K. Hilkert, W. Wolfsberger, T.K. Oleksyk, The first complete mitochondrial genome of *Diadema antillarum* (Diadematoida, Diadematidae), GigaByte. 2022 (2022) 1–12. https://doi.org/10.46471/gigabyte.73.

[28] PMC, PubMed Central Tagging Guidelines, NCBI. (2023). https://www.ncbi.nlm.nih.gov/pmc/pmcdoc/tagging-guidelines/article/style.html (accessed April 12, 2023).

[29] J. Wu, A. Rajesh, Y.-N. Huang, K. Chhugani, R. Acharya, K. Peng, R.D. Johnson, A. Fiscutean, C.D. Robles-Espinoza, F.M. de La Vega, R. Bao, S. Mangul, Virtual meetings promise to eliminate geographical and administrative barriers and increase accessibility, diversity and inclusivity, Nat Biotechnol. 40 (2022) 133–137. https://doi.org/10.1038/s41587-021-01176-z.

[30] S. Mangul, L.S. Martin, B. Langmead, J.E. Sanchez-Galan, I. Toma, F. Hormozdiari, P. Pevzner, E. Eskin, How bioinformatics and open data can boost basic science in countries and universities with limited resources, Nat Biotechnol. 37 (2019) 324–326. https://doi.org/10.1038/s41587-019-0053-y.

[31] International Science Council, Amplifying the voices of at-risk, displaced and refugee scientists, (2021).

[32] National Academies of Sciences Engineering and Medicine, Action Steps for Rebuilding Ukraine's Science, Research, and Innovation, (2022). https://www.nationalacademies.org/news/2022/06/action-steps-for-rebuilding-ukraines-science-research-and-innovation (accessed March 1, 2023).
