# Supplementary material for: Scientists without borders: lessons from Ukraine
Source: Gigascience. 2023 Jul 27;12:giad045. doi: 10.1093/gigascience/giad045 (PMC10372202; doi:10.1093/gigascience/giad045)
Supplement: giad045_Ukrainian_translation_Scientists_without_borders [file giad045_ukrainian_translation_scientists_without_borders.pdf]

OXFORD
(GIGA)n SCIENCE

GigaScience, 2023

Additional File S1. Author's Translation (Авторський переклад)

Вчені без кордонів: уроки з України

Валтер Вольфсбергер[1,+], Карішма Чхугані[2,+], Христина Щубелка[1], Аліна Фролова[3], Юрій Салига[4], Оксана Зленко[5], Михайло Арич[6], Дмитро Дзюба[7], Андрій Пархоменко[8], Володимир Смоланка[9], Зейнеп Г. Гюмюш[10], Ефе Сезгін[11], Алондра Діас-Ламейро[12], Віктор Р. Товт[13], Мегі Маці[14], Ерік Бортц[15], Федір Кондрашов[16], Патріція Мортон[17], Павел П. Лабай[18], Вероніка Ромеро[19], Якуб Главка[20,21], Сергій Мангул[2,22], * ,† і Тарас К. Олексик[1,23], *

[1]Department of Biological Sciences, Oakland University, Rochester, MI 48309-4479, USA
[2]Department of Clinical Pharmacy, USC Alfred E. Mann School of Pharmacy and Pharmaceutical Sciences, University of Southern California, Los Angeles, CA 90033, USA
[3]Institute of Molecular Biology and Genetics of National Academy of Sciences of Ukraine, Kyiv Academic University, Kyiv 03143, Ukraine
[4]Institute of Animal Biology NAAS, Lviv 79034, Ukraine
[5]National Scientific Center "Institute of Experimental and Clinical Veterinary Medicine," Kharkiv 61023, Ukraine
[6]Institute of Economics and Management, National University of Food Technologies, Kyiv 01601, Ukraine
[7]Department of Anesthesiology and Intensive Care, P.L. Shupyk NUHC Ukraine, Kyiv 04112, Ukraine.
[8]Department of Finance and Business Economics, Marshall School of Business, University of Southern California, Los Angeles, CA 90089, USA
[9]Department of Medicine, Uzhhorod National University, Uzhhorod 88000, Ukraine
[10]Department of Genetics and Genomic Sciences, Icahn School of Medicine at Mount Sinai, New York, NY 10029, USA
[11]Department of Food Engineering, Izmir Institute of Technology, Urla, Izmir 35330, Turkey
[12]Department of Biology, University of Puerto Rico at Mayaguez, Mayaguez 00681, Puerto Rico
[13]Aquatic Botany and Microbial Ecology Research Group, Balaton Limnological Research Institute, Tihany 8237, Hungary
[14]Stritch School of Medicine, Loyola University Chicago, Maywood, IL 60153, USA
[15]Department of Biological Sciences, University of Alaska, Anchorage, AK 99508, USA
[16]Institute of Science and Technology Austria, Klosterneuburg 3400, Austria
[17]Department of Sociology, Department of Public Health, Wayne State University, Detroit, MI 48292, USA
[18]Małopolska Centre of Biotechnology, Jagiellonian University, Kraków 30-348, Poland
[19]Department of Neurobiology, University of Utah, Salt Lake City, UT 84112, USA
[20]Price School of Public Policy, University of Southern California, Los Angeles, CA 90089-3333, USA
[21]Masaryk University, Brno, 60177 Czech Republic
[22]Department of Computational Biology, University of Southern California, Los Angeles, CA 90033, USA
[23]Department of Biology, Uzhhorod National University, Uzhhorod 88000, Ukraine
***Адреса для листування:** Taras K. Oleksyk, Department of Biological Sciences, Oakland University, Dodge Hall Rm 367, 118 Library Dr., Rochester, MI 48309-4479, USA; E-mail: oleksyk@oakland.edu; Serghei Mangul, Department of Clinical Pharmacy, School of Pharmacy, University of Southern California, 1540 Alcazar Street, Los Angeles, CA 90033, USA; E-mail: serghei.mangul@gmail.com
+Ці автори зробили однаковий внесок у цю роботу..
†Ці автори спільно керували цією роботою.
Примітка редактора: Щоб зробити цей огляд більш доступним для читачів, автори переклали фінальну версію цього рукопису українською мовою. Цей файл доступний як додатковий файл, S1.

Абстракт

Конфлікти та природні лиха впливають на все населення постраждалих держав. На додачу до тисяч зруйнованих і втрачених життів вони вкрай негативно позначаються й на науковому поступі всередині цих країн. Неспровоковане вторгнення росії в Україну, жахливий землетрус у Туреччині та Сирії, тривалі конфлікти на теренах Середнього Сходу — це лише деякі з сучасних прикладів. Мільйони людей загинули або були вимушено переміщені з невизначеним майбутнім. Ці події призвели до сильного руйнування інфраструктури, відсутності електроенергії, транспортних сполучень, доступу до послуг. Школи, університети та наукові центри зруйновані разом з даними, зразками та доробками десятиліть. Вчені з постраждалих регіонів стикнулися з коротко- та довгостроковими проблемами фінансування і постійного працевлаштування. У нинішньому взаємопов'язаному світі сутички та стихійні лиха більше не є виключно місцевими проблемами, бо вони призводять до майбутніх реальних наслідків на глобальному рівні. В цій статті, ми зосередилися на коротко- та довготривалому впливі війни на наукову спільноту України. Ми узагальнили уроки отримані під час цієї кризи, що можуть бути використані в усіх країнах, де вчені опиняються в небезпечних обставинах. Ми продемонстрували та систематизували приклади ефективних і дієвих механізмів, які можуть бути задіяні для підтримки дослідників, що опинилися в скрутному становищі, та обговорили способи застосування цих механізмів за підтримки міжнародного наукового товариства, а також інші критичні потреби. Встановлення контактів, надання можливостей для навчання та розвиток співпраці мають підвищити рівень включення та зв'язок між дослідниками, підтримати наукові досягнення в постраждалих спільнотах, а також прискорити відновлення науки після воєн і лих.

Ключові Слова: Конфлікти; Війна; Науковці; Європа; Україна; росія; Наука; Фінансування; Дистанційне навчання; Стипендіальні можливості; Біоінформатика;

Загальна Інформація

Час від часу, різні країни світу потрапляють у важкі обставини, спричинені політичними потрясіннями, війнами, економічними кризами та стихійними лихами [1]. Через це втрачаються та руйнуються мільйони життів, залучаються величезні обсяги допомоги, планування, та ресурсів на відновлення. В цьому огляді ми описали різні підходи та варіанти допомоги для науковців і студентів, що постраждали, були переміщені, або втратили зв'язок зі своїми професійними

спільнотами та зіштовхнулися з невизначеністю щодо майбутнього. В результаті обставин, професійні дослідники втрачають фінансування, робочі місця, зв'язки, налагоджене співробітництво та партнерів, а також цілі лабораторії разом зі зразками та базами даних, які можливо вже не вдасться відновити. Молоді студенти, як бакалаври, так і магістри, переривають або кидають наукові кар'єри. Як наслідок, цілі покоління талановитої молоді виключаються з навчального процесу, втрачають освітні можливості та сходять з наукового шляху. В таких ситуаціях, постраждалі наукові спільноти можуть звертатися за допомогою до колег з міжнародного співтовариства. Використовуючи як вдалі, так і невдалі прикладах таких зусиль, ми спробували окреслити підходи і шляхи протистояння майбутнім глобальним викликам.

Сила наукових спільнот в їхній різноманітності. Якщо ми прагнемо інтеграції, справедливості та різноманітності точок зору, об'єднання зусиль — це перший необхідний крок у скрутній ситуації. Спрямування ресурсів, часу та зусиль на користь колег, які переживають складні часи, — це данина давній науковій традиції підтримки колег в складних обставинах. Історії Альберта Ейнштейна, Ніколи Тесли та Марії Склодовської-Кюрі, - впливових науковців які втекли від війни або переслідувань, це яскраві приклади, що доводять: людство потребує міжнародної кооперації і співробітництва. Науковий світ стає щодалі все тіснішим, а спільноті зараз простіше допомагати дослідникам що опинилися у важких умовах, передусім завдяки світовим інвестиціям та міжнародним проєктам. Наприклад, для реагування на глобальні наукові виклики щодня утворюються такі міжнародні об'єднання як *Global Alliance for Genomics and Health* [2], *International Brain Initiative* [3], *Human Genome Project* [4] або *Large Hadron Collider* [5]. Ці та інші подібні проєкти стали надзвичайно успішними, оскільки забезпечили обмін ресурсами, досвідом і даними в світових масштабах. Спільний досвід таких об'єднань широко поширюється, обговорюється та розповсюджується у відкритих публікаціях та на наукових конференціях (очних, гібридних і дистанційних). Онлайн-препринти, журнали та видання вільного доступу вже зробили революцію в поширенні знань через спрощення доступу до інформації. Глобальні дослідницькі мережі виникли в багатьох галузях, а такі ініціативи як *Open Access* та *Open Data* [6] щодалі сприяють подальшому обміну знаннями. На кінець, потрібно також згадати рекомендації ЮНЕСКО про Відкриту Науку (*Open Science*) [7] які є цінним інструментом для підтримки вчених у кризових регіонах. Серед них: фінансування відкритих наукових проєктів, навчання та підтримка вчених через використання наукових інструментів і методик у відкритому доступі, розвиток інфраструктури відкритої науки для забезпечення доступних ресурсів і підтримки, необхідної для продовження дослідницької роботи та досягнення наукового прогресу.

Глобальні наукові мережі швидко розвиваються об'єднуючи роботу вчених і комп'ютерні технології відіграють у цьому процесі ключову роль. Завдяки розповсюдженню високошвидкісного доступу до Інтернету та цифрових інструментів, науковці з усього світу тепер можуть зв'язуватися і співпрацювати в режимі реального часу незалежно від свого місцезнаходження. Використання веб-технологій для наукового співробітництва потребує менше персонального контакту, ніж десять років тому. Особливо це стосується нових наук, таких як наприклад з ряду так званих «Оміків» (геноміки, транскриптоміки, протеоміки) та біоінформатики [8]. Ще не так давно, основною перепоною для Проєкту Геному Людини (*Human Genome Project*) та Проєкту 1000 геномів (*1,000 Genomes*) був збір зразків і молекулярних даних [9], а для міжнародної співпраці, спеціальні лабораторні навички та ресурси для збору зразків і секвенування на місцях були обов'язковими. Сьогодні ж, медичні та інші фенотипні дані можна легко накопичувати та поширювати між дослідниками та установами в цифровому форматі. Доступними стали і численні недорогі методи секвенування ДНК, РНК і білків, і дані отримані в результаті досліджень і високопродуктивного скринінгу, які можуть спільно аналізуватися міжнародними групами, залученими до спільних геномних та біоінформатичних досліджень [10–13]. Через 20 років після публікації геному людини, головною перешкодою з якою зіштовхується міжнародне співробітництво в геномних науках є брак доступу до потужного комп'ютерного обладнання та програмного забезпечення необхідного для аналізу величезних потоків вхідних оцифрованих даних. Нині ця проблема вирішується за допомогою вдосконалених комп'ютерних кластерів, збільшення обсягу цифрових сховищ і використання хмарних обчислень (*cloud computing*). Однак співпраця, яка базується на побудові глобальних мереж, може розвиватися лише за рахунок залучення все більшої кількості міжнародних учасників [14].

Онлайн-платформи та соціальні медіа допомагають створювати та об'єднувати мережі, що виходять за межі державних кордонів. Завдяки цьому дослідники можуть ділитися ідеями, співпрацювати над проєктами, та дискутувати з колегами з різних країн, з різними підходами і різним досвідом. Поява можливостей так званих хмарних (cloud) обчислень, візуалізації даних та використання інструментів штучного інтелекту роблять координацію досліджень ефективнішою, а відкриття — швидшими. Ці новітні технології руйнують кордони та бар'єри та дозволяють вченим об'єднуватися у способи, які були неможливі лише кілька років тому і допомагають прискорити науковий прогрес у всьому світі. Ми віримо, що надання можливостей віддаленої підтримки групам науковців тимчасово відрізаних від наукової спільноти внаслідок війни та ізольованих через політичні, економічні чи екологічні кризи, має важливе значення для світової науки. У даному огляді вивчивши приклади допомоги дослідникам, яка надавалася протягом одного року конфлікту в Україні, ми спробували визначити найефективніші механізми підтримки дослідників у постраждалих регіонах в цілому [1].

Міжнародна реакція наукової спільноти на війну в Україні

Відповідь на війну в Україні, що триває вже понад рік, є лише одним із прикладів реакції наукової спільноти на серйозну кризу. Одразу після вторгнення 24 лютого 2022 року волонтери, стурбовані негативним впливом подій на роботу колег, створили кілька онлайн-ресурсів. Здебільшого це були відкриті електронні таблиці з переліком короткострокових і довгострокових можливостей працевлаштування, стажування та інших видів пропонованої підтримки [15] (**Таблиця 1**). Деякі з цих ресурсів з'явилися спонтанно, створені занепокоєними колегами та товариствами. Зокрема, група співробітників з Університету Орегону опублікувала список лабораторій, які прагнули підтримати і прийняти вимушено переміщених українських вчених. Цей список швидко набув популярності і через 40 днів після створення містив понад 2000 вакансій в Європі та США (Лабораторії, що підтримують українських науковців, **Таблиця 1**). Подібний список також було створено в Університеті Сема Г'юстона в Техасі (База даних стипендій для України, **Таблиця 1**). Всього через два дні після початку війни з'явилася і найбільш масштабна ініціатива — *#ScienceForUkraine* [16]. Цей онлайн-проєкт об'єднав сотні науковців з різних країн, які на волонтерських засадах побудували центральну базу даних із переліком міжнародних можливостей для українських дослідників. Протягом війни з'явилося також кілька інших ініціатив, спрямованих на підтримку науковців, у тому числі *Ukrainian Scholar Placement Database*, розроблена Університетом Сема Г'юстона (**Таблиця 1**), з переліком грантових програм, доступних для українців.

Діяльність груп, що виникали, не обмежувалася лише дослідницькими можливостями: інші спільноти, такі як *Saving Ukrainian Cultural Heritage Online* (SUCHO, **Таблиця 1**), одночасно зайнялися веб-архівуванням, оцифруванням та збереженням спадщини закладів культури України (див. повний список у **Таблиці 1**). Лунали також заклики до підтримки тих студентів, які вже отримували освіту за кордоном, але більше не могли продовжувати навчання [17].

Таблиця 1. Посилання на ресурси для українських науковців, які було згадано у цій статті. Цей список не є вичерпним, оскільки інформація про нові можливості публікується щодня. Для отримання більш повної картини, будь ласка, пошукайте бази даних, такі як *#ScienceforUkraine,* а також інші ресурси, що згадуються в таблиці.

Ресурс	Опис	Розташування	Посилання
#ScienceforUkraine	Громадська група, що збирає та розповсюджує інформацію про можливості підтримки	Міжнародне	*https://scienceforukraine.eu*
Council for At-Risk Academics (CARA)	Співробітництво громадських організацій університетів Великої Британії для полегшення страждань і захисту освіти та науки	Великобританія	*https://www.cara.ngo*
ERA4Ukraine	Ініціатива ЄС для підтримки українських науковців шляхом надання інформації про всі актуальні заходи на європейському та національному рівнях	ЄС	*https://euraxess.ec.europa.eu/ukraine*
European Federation of Academies of Sciences and Humanities (ALLEA)	Європейський фонд для переміщених науковців	ЄС	*https://allea.org/european-fund-for-displaced-scientists*
ERC4Ukraine	Ініціатива Європейської Дослідницької Ради (*European Research Council* або ERC), що забезпечує науковців і допоміжний персонал з числа біженців тимчасовою роботою	ЄС	*https://erc.europa.eu/apply-grant/erc-ukraine*
The Guild	Європейська ініціатива з підтримки дослідницьких університетів	ЄС	*https://www.the-guild.eu/resources/the-guild-s-universities-supporting-researchers-ac.html*
IIE Scholar Rescue Fund	Міжнародна програма, що займається збором коштів та надає стипендії для біженців та переміщених науковців	ЄС	*https://www.scholarrescuefund.org*
Labs Supporting Ukrainian Scientists	Онлайн-база даних для допомоги у пошуку вакансій для переміщених з України науковців в Університеті Орегону	США	*https://tinyurl.com/yanb37ck*
Le Collège de France	Державній фонд з можливостями для переміщених науковців	Франція	*https://www.college-de-france.fr/*
OEG Connect	Відкритий освітній ресурс, створений для зв'язку, обміну та співпраці, щоби зробити навчання доступним	Міжнародне	*https://connect.oeglobal.org/*
Philipp Schwartz Initiative of the Humboldt Foundation	Програма для науковців, які зазнають суттєвої загрози власній безпеці у своїй країні, завдяки якій вони можуть продовжити свою роботу в німецьких університетах	Німеччина	*https://www.humboldt-foundation.de*
MSCA4Ukraine	Програма в структурі фонду Марії Склодовської-Кюрі, що забезпечує стипендіями дослідників з України	ЄС	*https://sareurope.eu/msca4ukraine/*
Saving Ukrainian Cultural Heritage Online (SUCHO)	Група волонтерів, що займаються веб-архівуванням, оцифруванням та збереженням спадщини українських закладів культури (не підтримує науковців)	Міжнародний	*https://www.sucho.org*
Scholars at Risk (SAR)	Міжнародна мережа захисту науковців під загрозою та підтримки академічної свободи	Міжнародне	*https://www.scholarsatrisk.org*
Scientists and Engineers in Exile or Displaced (SEED) program	Спільна програма національних академій для надання грантів	США та Польща	*https://www.nationalacademies.org/our-work/scientists-and-engineers-in-exile-or-displaced-seed-program*

**Ресурс**	**Опис**	**Розташування**	***Посилання***
Shevchenko Emergency Fund	Фонд для підтримки науковців від Наукового товариства імені Шевченка — найбільшої та найстарішої української наукової організації за кордоном	США	*https://shevchenko.org*
Swiss National Science Foundation	Державна програма з можливостями підтримки переміщених науковців	Швейцарія	*https://www.snf.ch/en*
The Association for Slavic, East European & Eurasian Studies	База даних ресурсів для підтримки українських науковців	США	*https://www.aseees.org/resources/help-displaced-scholars-ukraine*
The UK-Ukraine Twinning Initiative	Модель міжінститутської співпраці для розвитку міцного співробітництва між українськими та британськими університетами, що фінансується грантами *UKRI Research England*	Великобританія та Україна	*https://www.twinningukraine.com/*
Ukrainian Global University Initiative	Консорціум провідних українських освітніх установ та організацій для забезпечення українських науковців можливостями для якісного навчання та досліджень	Україна	*https://uglobal.university*
Ukrainian Scholar Placement Database	Онлайн-база даних для переміщених з України науковців на базі Університету Сема Г'юстона	США	*https://tinyurl.com/2f8csafa*

Мережі співробітництва, що існували ще до війни, також були використані для швидкого запуску та посилення підтримки науковців в Україні. Група таких ініціатив виникла на базі довоєнних структур, зокрема національних товариств та академій. Наприклад, Відділ Управління політики та глобальних справ Національних Академії Наук (NAS) США, який раніше займався науковою дипломатією між різними націями у співпраці з Польською академією наук (PAS) створили ініціативу «Вчені та інженери у вигнанні чи переміщені особи», для того щоб негайно підтримати кілька сотень українських науковців короткостроковими стипендіями на навчання за межами України (PAS-NAS SEED, **Таблиця 1**). Наукове Товариство Шевченка (НТШ, найбільша та найстаріша українська наукова організація за кордоном, заснована у 1873 році) почало створювати можливості для переміщених вчених за підтримки своїх членів із США та Канади. Більшість національних академій ЄС зробили заяви на підтримку України, а за кілька тижнів Європейська федерація академій наук і гуманітарних наук (*ALLEA*, **Таблиця 1**) завдяки пожертві від *Breakthrough Prize Foundation* почала приймати переміщених через війну науковців. Після того як мільйони українських біженців перетнули кордон ЄС і оселилися в різних європейських країнах, було створено ще більше можливостей національними організаціями, такими як Швейцарський національний науковий фонд, Польська академія наук (*PAS*) і *Le Collège de France*. Європейська комісія відкрила портал Європейського дослідницького простору для України, а *Horizon Europe* запропонувала безкоштовну участь у своїй додатковій програмі досліджень і навчання Євратом, аби підтримати українську наукову спільноту. Консорціум *MSCA4Ukraine*, що фінансується в рамках європейської програми імені Марії Склодовської-Кюрі теж почав надавати стипендії сотням переміщених дослідників з України.

Однак національні академії були лише одним із багатьох джерел підтримки. Найактивнішу допомогу почали надавати інтернаціональні дослідницькі мережі та професійні міжнародні наукові асоціації такі як *Scholars at Risk* (SAR, **Таблиця 1**), створена ще в 1999 році для захисту науковців, які опинилися під загрозою, а також для сприяння академічній свободі в усьому світі, та інші (*IIE Scholar Rescue Fund,* Ініціатива Філіпа Шварца Фонду Гумбольдта (*Philipp Schwartz Initiative of the Humboldt Foundation*) в Німеччині, Рада науковців групи ризику (*Council for At-Risk Academics*) у Великобританії тощо (**Таблиця 1)**. За підтримки національних урядів, приватних донорів, філантропів, а також великих корпоративних спонсорів вони швидко організували свої зусилля та почали створювати можливості для вимушено переміщених українців. Побратимська Ініціатива (*Twinning Initiative*) у Великобританії, заснована на безпосередньому співробітництва (моделі «інститут-інститут»), запустила грантову програму *UK-Ukraine Research and Innovation* від *UKRI Research England*, щоби дати можливість партнерам розвивати пряму дослідницьку та інноваційну співпрацю. Через відносно короткий період часу окремі університети, науково-дослідні інститути та інші академічні організації з усього світу почали самостійно пропонувати стипендії, оплачувані посади та інші види підтримки. Українські науковці з великою вдячністю зустріли волонтерські ініціативи та незмінну підтримку від низки університетів, академічних і фінансових установ, товариств, а також видавництв у всьому світі [18]. Ця допомога є вирішальною для відновлення та мінімізації наслідків війни для української академічної та наукової спільноти, а також відбудови наукових установ України. Мережі співробітництва, створені на початку війни, заклали основу для подальшої інтеграції українських дослідників у світову наукову спільноту. Вони продовжують створюють фундамент майбутнього успіху для більш формальних організаційних ініціатив за участю України, що зараз розробляються (наприклад, *Ukrainian Global University Initiative*, *Horizon Europe* тощо).

Незважаючи на всі зусилля, лише частина науковців змогли фізично переселитися за кордон і скористатися можливостями, отриманими завдяки міжнародній підтримці, але решта, по різним причинам, залишилися в країні. Причин цьому багато. По-перше, згідно з правилами воєнного стану в Україні, військовозобов'язані чоловіки віком від 18 до 60 років не можуть покидати країну. По-друге, чимало науковців добровільно вступили до лав Територіальної оборони або були призвані до Збройних Сил [19]. Багато хто просто не захотів покидати свої сім'ї та домівки заради короткочасних дослідницьких можливостей або змушенич був піклуватися про дітей чи родичів похилого віку, яких неможливо було взяти з собою за кордон. Навіть серед тих, хто мав можливість

виїхати, багатьох зупинили бюрократичні перепони при отриманні довгострокових віз.

Науковці, що залишилися в Україні, опинилися в несприятливих і небезпечних умовах. Багато університетів, особливо на сході країни, було знищено, закрито або перенесено в безпечніші регіони. Бюджети державних навчальних закладів скоротили на користь військових потреб. Хоча базові зарплати для викладачів переважно збереглися, вони і до того традиційно були дуже низькими. Основний прибуток активних науковців в країні зазвичай доповнювався доходами від грантових досліджень і понаднормової роботи, але ці можливості стали ще більш обмеженими [20]. Національні дослідницькі програми постраждали і далі страждають від проблем, пов'язаних з війною, а наукове фінансування було здебільшого припинене або заморожене. Проте, незважаючи на жахливу ситуацію, багато науковців в Україні продовжують академічну та дослідницьку діяльність.

Українська криза призвела до різних проблем і цілого комплексу дій у відповідь, які ґрунтувалися на багатьох факторах: історичних, культурних, економічних та політичних. У першу чергу, наукова спільнота відреагувала наданням можливостей для переселенців, які залишали Україну. Ця реакція пояснювалася тим що ряд недавніх криз викликаних економічною нестабільністю або війною, vв першу чергу призвели до появу великої кількості переміщених осіб і вченими з кризових регіонів у пошуках притулку. Наприклад, внаслідок політичної і економічної кризи, близько 2000 висококваліфікованих науковців з Венесуели, які написали третину всіх публікацій та представляли 15% всієї наукової спільноти країни, виїхали за кордон [21]. Нещодавно, ситуація пов'язана з відкликанням армії США з Афганістану зробила багатьох місцевих дослідників біженцями, а ті, хто залишився, втратили фінансування та опинилися під загрозою переслідувань [22]. Громадянська війна в Сирії, що триває з 2011 року та призвела до численних гуманітарних, економічних і політичних викликів, також привела до появи наукових біженців - студентів та дослідників, які потребують допомоги за межами своєї країни [23]. На початку війни, міжнародна наукова спільнота очікувала, що війна в Україні матиме подібні наслідки, тож негайно почала готуватися до напливу дослідників-біженців, яким потрібне було б житло та робота за межами країни.

Аналіз можливостей для українських науковців у базі даних *#ScienceForUkraine*

Щоби розуміти, які типи можливостей пропонувалися українським науковцям протягом першого року війни, ми дослідили та систематизували записи на веб-сторінці *#ScienceForUkraine* опубліковані напротязі одного року після вторгнення: з лютого 2022 до лютого 2023 року. Ця важлива ініціатива прагне допомогти українському науковому співтовариству та запобігти будь-яким перешкодам, з якими воно могло зіштовхнутися [16]. Хоча ця база даних може бути не повною, а деякі записи могли були видалені з неї (згідно з особистим спілкуванням авторів), *#ScienceForUkraine* все ще залишається найбільшою з публічно доступних і придатних для дослідження розподілу типів пропонованих можливостей.

Згідно нашого аналізу, в базі *#ScienceForUkraine* переважають пропозиції переїзду для роботи і навчання за межами України і помітно брак дистанційних можливостей, що не потребують виїзду за кордон (**Таблиця 2, Рисунок 1**). Волонтери ініціативи домагалися підвищення обізнаності наукових спільнот щодо ситуації, публікації нових можливостей для студентів та науковців по всьому світі, а також працювали з різноманітними європейськими грантовими установами, закликаючи академічну спільноту до допомоги. Ми використали стандартну класифікацію цієї бази даних: «оплачувані посади», «навчальні», «грантові програми» та «партнерства» (**Рисунок 1А**). Більшість можливостей становили «оплачувані посади». В схожих пропорціях розподілилися «навчальні» та «грантові програми» (**Таблиця 2, Рисунок 1А**).

Загалом, було опубліковано приблизно однакову кількість можливостей для «дослідників» та «студентів» всіх рівнів. Лише незначна кількість з них призначалася для «експертів» (**Рисунок 1Б**). Їх складно розподілити за окремими підкатегоріями, проте загалом вони охопили дослідницькі та аспірантські стипендії, а також робочі місця для науковців. Не дивно, що більшість з них припала на Європу (1293), а Північна Америка (США та Канада) посіла друге місце (181). Німеччина запропонувала найбільше оплачуваних посад, а за нею — Франція, Польща, Канада та США (**Рисунок 1В**). Німеччина також лідирує по кількості запропонованих освітніх можливостей і грантових програм (**Рисунок 1Г,Д**).

Загалом видн, що більшість ініціатив налаштовувалися на швидкий приплив біженців з України і переважно ґрунтувався на готовності українських науковців до переїзду за кордон. Огляд бази *#ScienceForUkraine* свідчить, що для більшості пропозицій передбачався виїзд, і лише незначна кількість можливостей призначалися для тих хто не міг залишити країну під час війни: 52 з 1871 доступних пропозицій були згадані як дистанційні а більшість передбачала можливість переїзду. Не дивлячись на критичну значущість ініціатив, які пропонують тимчасові чи постійні посади, вони були прийнятними лише для тої частини науковців, які вже виїхали раніше або мали таку можливість згодом. В результаті, дуже часто такі пропозиції могли лишитися невикористаними, що могло привести до розчарування як тих, хто їх пропонував, так і тих, хто не зміг ними скористатися.

Ефективні та дієві механізми для підтримки науковців та студентів з України

Ми сподіваємося використати досвід підтримки науковців в Україні після вторгнення, щоби розпочати дискусію щодо ефективності механізмів підтримки науковців і студентів в кризових регіонах в цілому. Ми вважаємо, що існує необхідність у обговоренні дієвості різних коротко- і довго-строкових стратегій допомоги науковцям під час активних фаз конфліктів та у періоди післявоєнного відновлення. Зокрема ми хочемо звернути увагу міжнародної спільноти на створення масштабованих механізмів, які можна пропонувати тисячам науковців, які покинули свої країни, але також тим, хто з різних причин залишився в країні де відбувається криза. Має сенс доповняти можливості, які передбачають переїзд такими, що можуть бути використані науковцями, які не можуть виїхати - потрібен широкий вибір дистанційних вакансій, можливостей залучення до співпраці з міжнародною науковою спільнотою, а також ефективні можливості для дистанційного навчання [1]. Така комбінована стратегія допоможе науці пережити важкі часи, зберегти темпи розвитку і зарадить з майбутньою інтеграцією до міжнародного наукового співтовариства. Ця стратегія також стане прикладом для інших спільнот у скритних та кризових ситуаціях. Таким чином, ми використовуємо досвід України як приклад власного досвіду співпраці, але наведені тут рекомендації можуть бути використані для підтримки подібних ініціатив в інших країнах.

Описані нами ефективні механізми підтримки можна поділити на дві групи (**Таблиця 3**). До першої відносяться прямі механізми, тобто ті, що передбачають встановлення зв'язків з конкретними українськими науковцями чи групами (**Таблиця 3, A-D).** До другої — опосередковані, які не передбачають таких контактів (**Таблиця 3, E-H**)

Дистанційна наукова співпраця передбачає обмін даними, написання спільних публікацій або ж обмін досвідом у визначеній сфері (**Таблиця 3, Група 1**). Ці стратегії можуть допомогти підтримати дослідників, які лишаються вдома, а також надати можливості для групової роботи та співпраці. Завдяки швидкому розвитку комп'ютерних технологій, дослідники з різних галузей можуть бути залучені до міжінституційних і міжнародних досліджень та вносити свій вклад віддалено: ця стратегія вже має доведену ефективність [24]. Окремим дослідникам найпростіше знайти та запросити колег зі своєї галузі, які працюють в країні, що зазнала кризи або агресії, долучитися до існуючого дослідження, або щоб започаткувати новий науковий проєкт який може призвести до публікації в міжнародних наукових журналах (**Таблиця 3, Група 1, А**). Такій підхід обіцяє швидкий індивідуальний ефект, а подання спільних рецензованих публікацій англійською мовою підвищує міжнародну інтеграцію науки, а також забезпечує сприяння розвитку та зміцнення наукового процесу на місцях.

Таблиця 2. Географія можливостей для українських науковців у базі даних *#ScienceForUkraine*

Тип	Всього Доступно	Європа	Північна Америка	Азія	Південна Америка	Океанія	Африка
Оплачувані посади	1,456 (83%)	1,293	181	44	11	12	2
Навчальні програми	170 (9%)	145	12	8	0	2	0
Грантові програми	67 (4%)	53	8	1	1	0	0
Партнерства	6 (<1%)	1	2	0	1	1	0
Інша допомога	77 (4%)	68	5	0	0	0	0
Тип не визначено	5 (<5%)	3	0	1	1	0	0
Разом		1,563	208	54	14	15	2

Якщо немає можливості або зацікавленості у безпосередній науковій співпраці, залишаються інші варіанти підтримки. Наприклад, при наявності коштів їх можна пожертвувати безпосередньо дослідним установам, щоби підтримати їх роботу у межах країни (**Таблиця 3, Група 1, Б**). Отримувачами коштів можуть бути наукові організації, державні та приватні університети, дослідницькі інститути та громадські організації, що підтримують науку. Якщо ж гроші відсутні, обмін іншими доступними ресурсами також може суттєво допомогти. Наприклад, надання доступу до наукових журналів, баз наукових даних, онлайн інструментів чи програмного забезпечення може бути вкрай необхідним для продовження дослідницької діяльності в кризових регіонах. Також, допомогти може обмін новинами та інформацією щодо можливостей пов'язаних з тематикою досліджень науковців у скрутних обставинах у соцмережах та на інших онлайн-платформах, таких як *#ScienceForUkraine* тощо (**Таблиця 3, Група 1, В**). Це може допомогти у пошуку потенційних співавторів, наставників та студентів, збільшити обізнаність про їх роботу та спрямувати до потенційних можливостей фінансування.

Світову спільноту потрібно заохочувати до активнішої реєстрації та участі у подіях, які проводяться в Україні (**Таблиця 3, Група 1, Г**). Деякі наукові організації та університети в Україні прямо під час війни організовували вебінари, онлайн-конференції та воркшопи, до дистанційної участі в яких запрошували іноземних колег. Наприклад, невдовзі після вторгнення Інститут молекулярної біології та генетики НАН України організував «Всеукраїнську конференцію з молекулярної та клітинної біології (з міжнародною участю)» (липень 2022).

Завдяки онлайн-конференціям з міжнародною участю, можна дізнатися про актуальні дослідження та познайомитися з потенційними співавторами в країні, забезпечити необхідну підтримку та посприяти зміцненню місцевої науки. Такі події мають безліч переваг. Зокрема, вони доступні для ширшого кола учасників і дозволяють зменшити витрати на проїзд. Разом з цим, є і декілька мінусів. Зокрема втрачається можливість неформального спілкування, з'являються складнощі з концентрацією уваги під час презентацій і потенційні технічні проблеми [25]. Цих проблем можна уникнути на конференціях гібридного формату, де це має бути враховано, щоби збільшити позитивний вплив на науковців під загрозою, що можуть брати участь тільки віртуально.

Дослідникам у кризових регіонах можна допомагати не тільки прямо, але опосередковано, завдяки модифікації політики наукових видавництв, фондів і закладів науки (**Таблиця 3, Група 2, Ґ-Є**). До прикладу, наукові журнали можуть підтримати українських науковців скасувавши для них плату за публікації та допомагаючи з перекладом і редагуванням. Запрошення ж українських колег до редакційних рад сприяє інтеграції (**Таблиця 3, Група 2, Ґ**). Деякі журнали вже зробили спеціальні збірки робіт українських вчених [26]. редакції також могли би додати підтримку багатомовності — публікувати версії статей рідними мовами авторів [27]. *PubMed Central* вже має політику, що дозволяє прийом до публікації неангломовних статей або статей з неангломовними частинами через договори між видавцем та *NLM* [28].

Організації та спільноти, що проводять наукові конференції, можуть скасовувати внески для науковців, які беруть віртуальну участь віддалено з кризового регіону. Таким чином в них можуть приймати участь ті дослідники, що не можуть покинути свої країни. Багато конференцій та конгресів вже проводяться у гібридному режимі: вони дозволяють як особисту так і дистанційну участь. Це дозволяє позбутися адміністративних перепон і добре підходить для науковців, які не можуть виїжджати за кордон [29]. Наукові товариства і асоціації також можуть допомогти через скасування реєстраційних внесків для науковців і дослідників з кризових регіонів, щоби ті могли брати участь у міжнародному науковому житті не покидаючи своїх країн (**Таблиця 3, Група 2, Д**). Під час написання цієї статті автори звернулися до організаторів деяких конференцій щодо спрощення доступу для науковців з України на цей рік. В результаті *The European*

Society of Human Genetics (ESHG) вирішили скасувати членський внесок 2023 для всіх українських генетиків, які хотіли долучитися до *ESHG* але залишилися в Україні, а також ж для тих, які були змушені залишити країну через війну. *The Society of Molecular Biology and Evolution (SMBE)* розробили гібридну схему заохочення науковців, які не можуть відвідати конференцію особисто: окремі учасники мали можливість подавати заявки, а організатори розглядали можливості скасування реєстраційного внеску в індивідуальному порядку (за особисту чи онлайн-участь) для науковців, які не мають коштів для оплати. *The International Society of Computational Biology (ISCB)* запропонував найменший можливий внесок за онлайн-участь в конференції *ISMB/ECCB 2023* для дослідників, які залишилися в Україні чи були змушені виїхати через війну.

Дистанційне навчання (**Таблиця 3, Група 2, E**) — це ще один важливий інструмент для залучення допомоги від наукової спільноти [30]. В Україні є групи, що прагнуть брати участь та бути частиною наукових співробітництва яка передбачає невелику додаткову підготовку. Дійсно, через розвинену освітню систему Україна має велику кількість студентів-інформатиків. Як теперішні студенти так і нещодавні випускники вузів добре підходять для дистанційної роботи й обчислювальних досліджень, однак після завершення ВНЗ часто не продовжують наукову кар'єру, бо йдуть працювати на добре оплачувані аутсорсингові роботи в різних галузях, чому сприяють фінансові стимули, пропоновані рекрутинговими компаніями. В новій реальності, створеній вторгненням, ці талановиті люди стикаються з обмеженими можливостями в галузі науки. У той самій час, оскільки продовження навчання

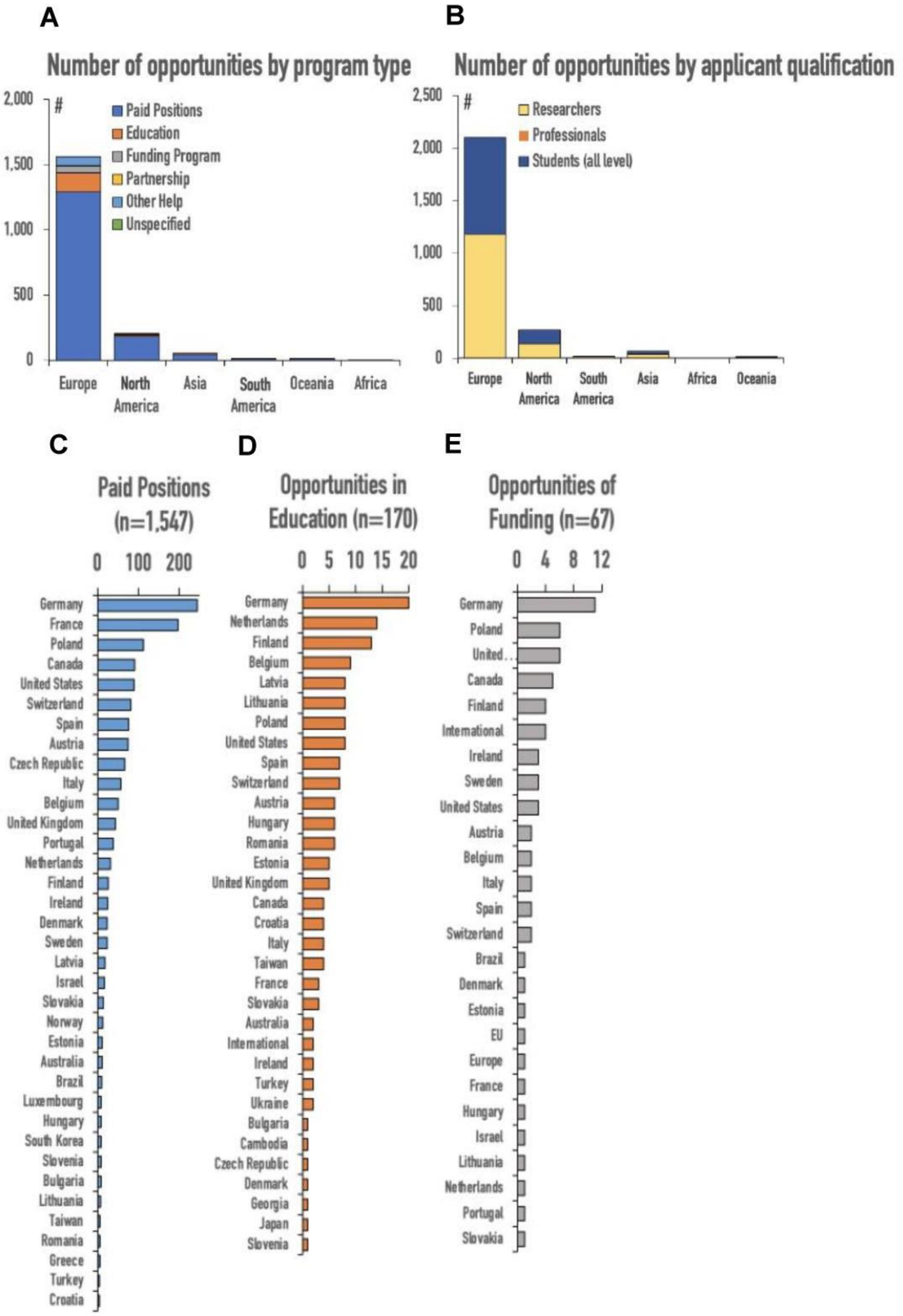

Рисунок 1. Географія можливостей для українських науковців в базі даних *#ScienceForUkraine* на протязі одного року після початку конфлікту (лютий 2022 — лютий 2023). **(А).** Кількість можливостей за типом програми. Більшість з 1871 пропозицій можуть бути класифіковані як оплачувані. Зокрема це дослідницькі та докторантські стипендії, а також декілька позицій для наукової роботи. Більшість з них припадають на Європу (1293), Північну Америку та дистанційні варіанти (181). Навчальні можливості та фінансування розподілені у схожих пропорціях. **(Б).** Кількість можливостей за кваліфікацією кандидатів. Переважно розподілені приблизно порівну між дослідниками та студентами всіх рівнів, при чому досить небагато з них призначені для експертів. **(В, Г і Д).** Розподіл за типами можливостей (перерахованих в Рис. 1.А) за країнами. Загальна кількість можливостей на категорію вказано в дужках. Більшість з них припадає на Німеччину.

Таблиця 3. Механізми підтримки науковців і студентів з кризових регіонів. **Група 1 (А-Г)** включає заходи спрямовані на застосування усталених контактів з науковцями чи дослідницькими групами в Україні. **Група 2 (Ґ-Є** включає непряму допомогу українським вченим — без безпосереднього контакту з науковцем чи групою дослідників.

	#	Механізм	Опис
Група 1	А	Безпосередня дистанційна наукова співпраця	Науковець або дослідник може віддалено співпрацювати безпосередньо з українським представником в рамках спільного наукового проєкту.
	Б	Перерахунок коштів та доступ до ресурсів	Кошти можуть перераховуватися безпосередньо на рахунки закладів для підтримки їх роботи.
	В	Інформування про наукові можливості	Інформування колег про можливості, що можуть бути втрачені або пропущені через обмежений доступ до баз наукових даних або через мовний бар'єр.
	Г	Участь у онлайн-заходах у межах країни	Участь у вебінарах, онлайн-конференціях та воркшопах місцевих організаторів в кранах що переживають кризу.
Група 2	Ґ	Заохочення до публікацій у наукових журналах	Заохочення журналів до підтримки дослідників шляхом скасування плати за публікації, допомоги з перекладом і редагуванням, включно зі статтями, опублікованими рідною мовою авторів.
	Д	Заохочення до участі у наукових конференціях	Заохочення організаторів наукових конференцій надавати знижки чи безкоштовний доступ до реєстрацій і членства.
	Е	Створення можливостей для дистанційного навчання	Організація та заохочення інших до організації онлайн-навчання, для забезпечення конкурентоспроможності кандидатів для різноманітних посад, пропонованих міжнародною науковою спільнотою.
	Є	Заохочення надання грантів та започаткування нових грантових можливості для міжнародної співпраці	Заохочення грантових агенцій, некомерційних організацій, державних фондів до започаткування нових грантових можливостей для міжнародної співпраці, що надає пріоритети на спільні дослідження, наукові проєкти та програми.

дає відстрочку від мобілізації, багато з них почали обирати академічні кар'єри. Це створило пул потенційних дослідників, які не можуть залишити країну, мають необхідні навички та прагнуть дистанційної співпраці у наукових проєктах. Навчання цієї окремої групи може сприяти створенню біоінформатичної спільноти, яку легко залучати до міжнародної співпраці [30]. Разом з тим, навчання передбачає фінансування, тож ми закликаємо грантові агенції започатковувати ініціативи для навчання науковців-біоінформатиків в Україні. Це дозволить залучати їх до великих міжнародних консорціумів дистанційно, а також у започаткувати співпрацю зі світовою спільнотою. Брак мовних навичок також може створювати додаткові бар'єри, оскільки не всі науковці мають достатній рівень англійської.

Необхідне фінансування для заохочення та освіти науковців, яким вони необхідні для міжнародної співпраці (**Таблиця 3, Група 2, Є**). Найбільш придатний до масштабування та гнучкий механізм — дистанційне навчання та платні дистанційні роботи, а також залучення відповідного фінансування для їх забезпечення. Онлайн-бази даних якийсь час вирішували деякі з цих проблем, однак багато з них залишаються та перешкоджають їхній ефективності. До прикладу, багато з розміщених вакансій не обов'язково спрямованні на рекрутинг українських науковців, які перебувають у скрутних обставинах, натомість є частиною маркетингу для загальнодоступних позицій: українці змушені конкурувати з іншими кандидатами, що не постраждали від війни. Навіть якщо студенти та дослідники зацікавлені в цих можливостях, їм доводиться зорієнтуватися в специфіці систем освіти відповідних країн. Через комбінацію політичних, економічних і культурних причин багато українських науковців, яким ці можливості були б корисні, не можуть за них змагатися, оскільки їм бракує необхідних навичок, щоб знайти роботу поза межами країни. Також часто вони просто не відповідають вимогам доступних вакансій. Якщо посаду не було створено спеціально для такого випадку, конкурентоспроможність потребує додаткового навчання. Для вирішення цих проблем ми пропонуємо створення платформи, що зможе з'єднати українських науковців та інформатиків з лабораторіями та кафедрами, які хочуть запропонувати дистанційне наставництво та працевлаштування [31].

Протягом пандемії COVID-19 ми побачили, що дистанційне навчання — це не тільки реально, а й ефективно, особливо якщо викладачі креативні, а студенти — вмотивовані. Викладачі, які створюють онлайн-ресурси, можуть об'єднатися з колегами, які перебувають у кризових регіонах для спільного викладання курсів за допомогою онлайн технологій. Навчальні сеанси можуть проводитися як синхронно, так і асинхронно (із записом на випадок відсутності енергопостачання чи Інтернету). Різні викладачі можуть викладати різні частини курсу. Студенти з різних країн можуть би навіть робити спільні проєкти, а робота могла би бути розділена відповідно до наявних ресурсів: поки одні займалися би лабораторною частиною, інші могли би фокусуватися на аналізі даних. Разом вони могли би генерувати звіти чи наукові статті, започатковуючи нове покоління міжнародної співпраці. Для цього також потрібні відповідні навчальні матеріали, а ініціативи, такі як *OER* (*Open Education Resources*) могли би їх надати. Історично, *OER* зосереджувалися на молодших студентах і учнях, але зараз ця спільнота працює над розширенням можливостей (**Таблиця 1, *OEG Connect***).

Світовій науковій спільноті та міжнародним агентствам необхідно терміново розробити та реалізувати скоординований план допомоги, націлений на науковців, які перебувають у небезпеці та залишаються в Україні. Деякі з авторів цієї статті вже створили міждисциплінарну групу дослідників з України, США, Великої Британії, Німеччини та інших країн, яких непокоїть нестабільна ситуація в науці в державах під час війни. Її учасники обговорюють необхідність створення ефективних і дієвих механізмів для підтримки дослідників та студентів і займаються поширенням цієї інформації. Уряди та приватний сектор мають започаткувати нові гранти для міжнародного співробітництва, зокрема для спільних досліджень та академічних проєктів і програм [31]. Серед ранніх прикладів таких зусиль можна навести *Action Steps for Rebuilding Ukraine's Science, Research, and Innovation* від Акдемії Наук Украни і Польської Академії Наук [32], які також згадують необхідність створення дистанційних можливостей, однак не наводять варіантів їх впровадження. Серед міжнародних агентств можна навести *United States Agency for International Development (USAID)* або *The United Nations Educational, Scientific and Cultural Organization (UNESCO)*. Вони надають гуманітарну допомогу та підтримують соціальний і економічний розвиток, а також міжнародне співробітництво. Однак, незважаючи на всесвітню присутність, а також на

велику кількість окремих лабораторій та кафедр, які прагнуть навчати та залучати науковців до своїх актуальних проєктів, їм

Ефективні механізми дистанційної підтримки вчених у кризових умовах

Оскільки наука в світі стає все більш взаємопов'язаною, вчені в країнах, уражених війною, політичними протиріччями, стихійними лихами, екологічними кризами чи економічними труднощами можуть продовжувати функціонувати у складі міжнародної спільноти та брати участь у дослідницькому процесі дистанційно. Завдяки новим можливостям вони можуть продовжувати спільну роботу незважаючи на географічні кордони, не обмежуючись визначеними дисциплінами, і мають змогу допомагати міжнародним зусиллям у боротьбі з глобальними викликами.

Уроки, винесені з поточної кризи в Україні, спричиненої непровокованим російським вторгненням, можуть бути застосовані в інших подібних ситуаціях. Використовуючи Україну як приклад, ми визначили декілька можливих шляхів допомоги науковим спільнотам, які переживають складні часи. Звісно, це не обмежується лише Україною, а може бути застосовано в інших ситуаціях. На жаль, існує занадто багато подібних прикладів, деякі з яких виникли в результаті війни в Україні, а інші — через переслідування в Венесуелі, Афганістані та Сирії, а також природні катастрофи: від ураганів Марія і Фіона в Пуерто-Рико і до останніх землетрусів в Туреччині та Сирії. У цій статті ми навели кілька ефективних і реальних шляхів підтримки вчених та студентів у країнах, які знаходяться у складних умовах або страждають внаслідок військової, політичної, економічної чи екологічної кризи (**Таблиця 2**). Список можливостей не є вичерпним і призначений почати, а не завершити дискусію з цього приводу.

Створення ресурсів, подібних до *#ScinceForUkraine* — було швидким і вдалим прикладом, що може бути застосовано та швидко адаптовано у випадку політичних криз чи війни, а також до територій стихійних лих. Багато з можливостей, перерахованих у цій та подібних базах даних не були спеціально створені для найму українців. Ці платформи використовують для залучення української аудиторія для закриття існуючих вакансій. Та навіть враховуючи це, ефект був позитивний, оскільки науковці в пошуку можливостей одразу отримують безліч варіантів. Однак, найкращим рішенням було би започаткування нових дослідницьких грантів для міжнародної співпраці, зокрема спільних досліджень та академічних проєктів і програм.

Зараз вкрай необхідно розпочати обговорення дієвого довготермінового плану для перебудови науки та створення дослідницьких можливостей. Потрібно створити програми та механізми, які дозволять науковцям, що не можуть покинути свої країни, опановувати сучасні навички дистанційно, а потім ділитися ними у своїх спільнотах на місці. Для дослідників, які лишаються в країні, перехід до обчислювальних досліджень на основі відкритих даних може бути гарним рішенням, оскільки багато з потрібних навичок можна набути та оцінити дистанційно з будь-якого місця. Систематичне навчання, необхідне для здобуття сучасних аналітичних навичок, можна реалізувати шляхом розробки планів, які передбачатимуть співпрацю з провідними институтами світу. Дистанційний формат роботи може застосовуватися не тільки для обчислювальних досліджень на основі відкритих даних, знайомих для читачів цього журналу, але й для гуманітарних і соціальних наук. Необхідно створювати дистанційні механізми, щоби спеціалісти світового рівня могли навчати та тренувати студентів і докторантів [32]. Залучення керівників університетів для створення дистанційних заходів є важливим, однак це досі не загальна практика. Фінансові агенції мають не вистачає коштів та механізмів для підтримки та залучення науковців з кризових регіонів.

забезпечити відповідні гранти для підтримки цих напрямків. Інтеграція нових галузей досліджень, які не покладаються на фізичну інфраструктуру, а натомість ґрунтуються на обчислювальних дослідженнях, можуть пришвидшити відбудову української наукового прогресу в порівнянні з фізичною інфраструктурою.

Доступність даних

Можливості для українських науковців за 1 рік з моменту початку конфлікту (лютий 2022 - лютий 2023) є публічно доступними в базі даних #ScienceForUkraine.

Конфлікти інтересів

Автори заявляють, що у них немає конфлікту інтересів.

Фінансування

Всі автори волонтерили свій час. Робота над цією статтею не була фінансово підтримана.

Додатковий Файл

S1 Легенда: Український переклад фінального рукопису.

Внески Авторів

Валтер Вольфсбергер (Концептуалізація [рівний], Кураторство даних [рівний], Формальний аналіз [лідер], Адміністрування проекту [підтримка], Валідація [підтримка], Візуалізація [рівний], Написання - оригінальний проект [рівний], Написання - рецензування та редагування [рівний]), Карішма Чугані (Концептуалізація [підтримка], Кураторство даних [рівний], Формальний аналіз [підтримка], Візуалізація [підтримка], Написання - оригінальний проект [рівний], Написання - рецензування та редагування [підтримка]), Христина Щубелка (Кураторство даних [рівний], Дослідження [підтримка], Написання - оригінальний проект [підтримка]), Аліна Фролова (Дослідження [підтримка], Ресурси [підтримка], Написання - оригінальний проект [підтримка]), Юрій Салига (Концептуалізація [рівний], Дослідження [підтримка], Написання - оригінальний проект [підтримка], Написання - рецензування та редагування [підтримка]), Оксана Зленко (Дослідження [підтримка], Написання - оригінальний проект [підтримка], Написання - рецензування та редагування [підтримка]), Михайло Арич (Дослідження [підтримка], Написання - оригінальний проект [підтримка], Написання - рецензування та редагування [підтримка]), Дмитро Дзюба (Дослідження [підтримка], Написання - оригінальний проект [підтримка], Написання - рецензування та редагування [підтримка]), Андрій Пархоменко (Дослідження [підтримка], Написання - оригінальний проект [підтримка], Написання - рецензування та редагування [підтримка]), Володимир Смоланка (Дослідження [підтримка], Написання - оригінальний проект [підтримка], Написання - рецензування та редагування [підтримка]), Зейнеп Н. Гюмюш (Дослідження [підтримка], Написання - оригінальний проект [підтримка], Письмо - рецензування та редагування [підтримка]), Ефе Сезгін (Дослідження [підтримка], Написання - оригінальний проект [підтримка], Написання - рецензування та редагування [підтримка]), Алондра Діас-Ламейро (Дослідження

[підтримка], Написання - оригінальний проект [підтримка], Письмо - рецензування та редагування [підтримка]), Віктор Р. Товт (Дослідження [підтримка], Написання - оригінальний проект [підтримка], Написання - рецензування та редагування [підтримка]), Мері Мачі (Ресурси [підтримка], Написання - оригінальний проект [підтримка], Письмо - рецензування та редагування [підтримка]), Ерік Бортц (Дослідження [підтримка], Написання - оригінальний проект [підтримка], Написання - рецензування та редагування [підтримка]), Федір Кондрашов (Дослідження [підтримка], Написання - оригінальний проект [підтримка], Написання - рецензування та редагування [підтримка]), Патріція Мортон (Методологія [підтримка], Написання - оригінальний проект [підтримка], Письмо - рецензування та редагування [підтримка]), Павел П. Лабай (Дослідження [підтримка], Написання - оригінальний проект [підтримка], Написання - рецензування та редагування [підтримка]), Вероніка Ромеро (Дослідження [підтримка], Письмо - оригінальний проект [підтримка], Написання - рецензування та редагування [підтримка]), Якуб Главка (Дослідження [підтримка], Написання - оригінальний проект [підтримка], Написання - рецензування та редагування [підтримка]), Сергей Мангул (Концептуалізація [рівний], Кураторство даних [рівний], Формальний аналіз [рівний], Дослідження [рівний], Методологія [рівний], Адміністрування проекту [рівний], Ресурси [рівний], Керівництво [рівний], Візуалізація [рівний], Написання - оригінальний проект [рівний], Написання - рецензування та редагування [рівний]) Тарас Олексик (Концептуалізація [рівний], Кураторство даних [рівний], Формальний аналіз [рівний], Дослідження [рівний], Методологія [рівний], Адміністрування проекту [рівний], Ресурси [рівний], Керівництво [рівний], Візуалізація [рівний], Написання - оригінальний проект [рівний], Написання - рецензування та редагування [рівний])

Подяки

Нашу статтю присвячено волелюбним людям по всьому світі та українському народу, що бореться за нашу свободу. Ми особливо вдячні Аніті Бандровській (Anita Bandrowski), Олександрі Іващенко (Oleksandra V. Ivashchenko) та Саніті Рейнсоун (Sanita Reinsone) за корисні відгуки, цінну критику та пропозиції, надані під час підготовки цього рукопису.

Список літератури

Chhugani K, Frolova A, Salyha Y, et al. Remote opportunities for scholars in Ukraine. Science 2022;378:1285–6.

THE GLOBAL ALLIANCE FOR GENOMICS AND HEALTH. A fed- erated ecosystem for sharing genomic, clinical data. Science 2016;352:1278–80.

Yuste R, Bargmann C. Toward a global brain initiative. Cell 2017;168:956–9.

Gibbs RA. The Human Genome Project changed everything. Nat Rev Genet 2020;21:575–6.

Horyn L. The Large Hadron Collider. In A Search for Displaced Lep- tons in the ATLAS Detector. Springer Nature Switzerland AG; 2022:21–29.https://doi.org/10.1007/978-3-030-91672-5_3

Miedema F. Open Science: The Very Idea. Dordrecht: Springer Netherlands; 2022.

UNESCO. UNESCO Recommendation on Open Science. Paris: UN- ESCO Headquarters (Paris)–UNESCO Archives (Paris); 2021.

Cirillo D, Valencia A. Big data analytics for personalized medicine. Curr Opin Biotechnol 2019;58:161–7.

Li J, Chen H, Wang Y, et al. Next-generation analytics for omics data. Cancer Cell 2021;39:3–6.

Hosmani PS, Shippy T, Miller S, et al. A quick guide for student-driven community genome annotation. PLoS Comput Biol 2019;15:e1006682.

Ryder EF, Morgan WR, Sierk M, et al. Incubators: building community networks and developing open educational resources to integrate bioinformatics into life science education. Biochem Mol Biol Educ 2020;48:381–90.

Cantelli G, Cochrane G, Brooksbank C, et al. The European Bioinfor- matics Institute: empowering cooperation in response to a global health crisis. Nucleic Acids Res 2021;49:D29–37.

Oleksyk TK, Wolfsberger WW, Shchubelka K, et al. The Pioneer Advantage: filling the blank spots on the map of genome diversity in Europe. Gigascience 2022;11:giac081https://doi.org/10.1093/gigascience/giac081.

Fedoroff Nv. Science diplomacy in the 21st century. Cell 2009;136:9– 11.

Maryl M, Ivashchenko Ov, Reinfelds M, et al. Addressing the needs of Ukrainian scholars at risk. Nat Hum Behav 2022;6: 746–7.

Rose M, Reinsone S, Andriushchenko M, et al.. 3 Months since Russia's invasion in Ukraine. SSRN J. February 26–May 31, 2022.https://doi.org/10.2139/ssrn.4139263

Heyman A. OU's Ukrainian community ask administra- tion for financial assistance. Oakland Post. 2022. https://oaklandpostonline.com/41529/campus/ous- ukrainian- community- ask- administration- for- financial- assistance/. Accessed 1 March 2023.

Klimash A. Ukraine's chemists persevere through a year of war. Chemistry World. 2023. https://www.chemistryworld.com/opinion/ukraines- chemists- persevere- through- a- year- of - war/40170 35.article. A ccessed 11 April 2023.

Gaind N. How three Ukrainian scientists are surviving Russia's brutal war. Nature 2022;605:414–6.

Maryl M, Jaroszewicz M, Degtyarova I et al., Beyond re- silience: professional challenges, preferences, and plans of Ukrainian researchers abroad. *Zenodo*. 2022. https://doi.org/10.5281/zenodo.7380509 Accessed 11 April 2023.

Casey N. Hungry Venezuelans flee in boats to escape economic collapse. NY Times. 2016. https://www.nytimes.com/2016/11/25/world/americas/hungry- venezuelans- f lee- in- boats- to- escape - economic- collapse.html?smid=url- share. A ccessed 1 March 2023.

Mallapaty S. Afghanistan's terrified scientists predict huge research losses. Nature 2021;597:15–16.

Stoepler TM, Creswell JE, Anthis NJ, et al. The role of science diplomacy in international crises: Syria as a case study. Sci Dipl 2017;6. https://www.sciencediplomacy.org/article/2017/role - science-diplomacy-in-international-crises-syria-case-study. A ccessed 1 March 2023.

Hunter P. Remote working in research. EMBO Rep 2019;20 https://doi.org/10.15252/embr.201847435.

Levitis E, van Praag CDG, Gau R, et al. Centering in- clusivity in the design of online conferences— an OHBM–open science perspective. Gigascience 2021;10.giab051https://doi.org/10.1093/gigascience/giab051.

Cherninskyi AO. Global excellence in cellular neuropathology: Ukraine. *Front Cell Neurosci*. 2023. https://www.frontiersin.org/re search- topics/46571/global- excellence- in- cellular- neuropatho logy-ukraine. Accessed 1 March 2023.

Majeske AJ, Mercado Capote AJ, Komissarov A, et al. The first com- plete mitochondrial genome of *Diadema antillarum* (Diadema- toida, Diadematidae). GigaByte 2022;2022:1–12.

PMC. PubMed Central Tagging Guidelines, NCBI. 2023. https://www.ncbi.nlm.nih.gov/pmc/pmcdoc/tagging- guidelines/article/style.html. Accessed 12 April 2023.

Wu J, Rajesh A, Huang Y-N, et al. Virtual meetings promise to elim- inate geographical and administrative barriers and increase ac- cessibility, diversity and inclusivity. Nat Biotechnol 2022;40:133–7.

Mangul S, Martin LS, Langmead B, et al. How bioinformatics and open data can boost basic science in countries and universities with limited resources. Nat Biotechnol 2019;37:324–6.
International Science Council. Amplifying the voices of at-risk, dis- placed and refugee scientists. 2021.
National Academies of Sciences Engineering and Medicine. Action steps for rebuilding Ukraine's science, research, and innovation. 2022. https://www.nationalacademies.org/news/2022/06/action-steps- for- rebuilding- ukraines- science- research- and-innovatio n. Accessed 1 March 2023.
