# Supplementary material for: Scientists without borders: lessons from Ukraine
Source: Gigascience. 2023 Jul 27;12:giad045. doi: 10.1093/gigascience/giad045 (PMC10372202; doi:10.1093/gigascience/giad045)
Supplement: giad045_Response_to_Reviewer_Comments_Original_Submission [file giad045_response_to_reviewer_comments_original_submission.pdf]

Author's Response To Reviewer Comments

Close

We include a point-by-point response to the reviewers' comments, detailing the modifications we have made

Reviewer 1:

Page 9
Polish Academy of Sciences quickly created the Scientists and
Engineers in Exile or Displaced initiative to support several hundred Ukrainian scholars with
short term stipends for studies outside of Ukraine (PAS-NAS SEED, Table 1).

Corrected: Polish Academy of Sciences quickly created the Scientists and Engineers in Exile or Displaced initiative to support several hundred Ukrainian scholars with
short-term stipends for studies outside of Ukraine (PAS-NAS SEED, Table 1).

Page 15
At the same time, there several issues, including the loss of informal networking opportunities, the difficulty of maintaining attention during online presentations, and the potential for technical problems [25].

Corrected: At the same time, there are several issues, including the loss of informal networking opportunities, the difficulty of maintaining attention during online presentations, and the potential for technical problems [25].

Page 16
Particularly, because these positions are not really geared toward Ukrainian scholars, candidates must compete with other applicants that are not disadvantaged by the war.

Corrected: Particularly, because many of these positions are general advertisements for positions available, and not specifically geared toward Ukrainian scholars at risk, candidates must compete with other applicants that are not disadvantaged by the war.

Page 17
we suggest that a platform needs to be developed that could match scientists and computer experts with laboratories and faculty

Corrected: we suggest that a platform needs to be developed that could match scientists and computer experts with laboratories and faculties willing to offer remote mentorships or employment.

Page 17
work could be divided based on availability of resources, some students could do lab work, while others might focus on data analysis

Corrected: work could be divided based on the availability of resources, some students could do lab work, while others might focus on data analysis

Page 17
created an interdisciplinary group consisting of researchers
from countries, including Ukraine, the United States, UK, Germany, and others

Corrected: created an interdisciplinary group consisting of researchers
from countries, including Ukraine, the United States, the UK, Germany, and others

Page 17
Another example of a decision making authority is the United States Agency for International Development (USAID)

Corrected: Another example of a decision-making authority is the United States Agency for International Development (USAID)

Close
