# Supplementary material for: Scientists without borders: lessons from Ukraine
Source: Gigascience. 2023 Jul 27;12:giad045. doi: 10.1093/gigascience/giad045 (PMC10372202; doi:10.1093/gigascience/giad045)
Supplement: giad045_Response_to_Reviewer_Comments_Revision_1 [file giad045_response_to_reviewer_comments_revision_1.pdf]

Author's Response To Reviewer Comments

Close

Dear Dr. Nogoy

Thank you for accepting our manuscript.

These sections have been added directly into the new draft of the manuscript.

Declarations

Data Availability: Opportunities for Ukrainian scientists in one year since the start of the conflict (February 2022-February 2023) are publicly available in the #ScienceForUkraine database.

Competing interests: The authors declare that they have no competing interests.

Funding: All authors volunteered their time. No funding supported work on this article.

New Software: There was no new software application associated with this article.

Author contributions: TKO, SM, KC, WWW and YS came up with the conceptual idea; WWW and KC performed the data curation; WWW and KC performed the Formal analysis; TKO, SM KC and WWW developed methodology; TKO and SM coordinated and supervised the collaboration; WWW and TKO visualized the data; All authors contributed to the original draft, review and editing. All authors read and approved the final manuscript.

I hope you will find these answers satisfactory

Taras Oleksyk

Close
